# Comparative Interactome Analysis Reveals Architectural Principles Governing K^+^ Channel Function in Cancer

**DOI:** 10.3390/ijms27135862

**Published:** 2026-06-29

**Authors:** Soha Sadeghi, Jesusa Capera, Giulia Battistello, Veronica Carpanese, Antonio Felipe, Ildikò Szabò, Vanessa Checchetto

**Affiliations:** 1 Department of Biology, University of Padova, 35131 Padova, Italy or sadeghi.soha@gmail.com (S.S.);; 2 Molecular Physiology Laboratory, Department de Bioquımica i Biomedicina Molecular, Institut de Biomedicina (IBUB), Universitat de Barcelona, 08028 Barcelona, Spain; 3 Nuffield Department of Orthopaedics Rheumatology & Musculoskeletal Sciences, The Kennedy Institute of Rheumatology, University of Oxford, Oxford OX1 2JD, UK

**Keywords:** potassium channels, protein–protein interactions, interactome, cancer signalling

## Abstract

Potassium (K^+^) channels have been frequently linked to cancer progression; however, their contribution varies across tumour types and experimental models. This heterogeneity indicates that gene-level characteristics such as expression, co-expression, or mutational status are inadequate for explaining channel involvement in oncogenic signalling. Here, we performed a cross-study comparison of experimentally validated K^+^ channel interactomes, we show that K^+^ channel regulation is highly context-dependent and does not exhibit conserved pan-cancer signatures. By directly comparing proximity-labeling and affinity-purification datasets across different K^+^ channel families, we identify a limited number of recurrent organizational architectures rather than universal signalling modules. KCa3.1 (encoded by *KCNN4*), Kir2.1 (*KCNJ2*), and TASK-1 (*KCNK3*) assemble signalling-permissive interactomes integrating adhesion complexes, junctional scaffolds, vesicular trafficking pathways, and receptor-associated signalling nodes. In contrast, Kv11.1 (encoded by *KCNH2*) displays an interactome predominantly enriched for proteostasis and endoplasmic reticulum–associated components, indicating a proteostasis-centered organizational profile with comparatively limited signalling integration. Kv1.3 (encoded by *KCNA3*), instead, consistently associates with mitochondrial and metabolism-linked proteins and functionally connects metabolic state to downstream transcriptional regulators, rather than regulating its own transcription. Higher-order intersection and pathway-specific analyses indicate that functional convergence across the above channels emerges from shared architectural principles rather than extensive molecular overlap. In conclusion, this study identifies interactome architecture as a central organizational level for understanding K^+^ channel function in cancer. The integration of pan-cancer gene-level analyses with systematic comparison of interaction architectures offers a coherent framework for interpreting the functional heterogeneity observed across channels, families, and tumor contexts. This perspective suggests that therapeutic strategies may benefit from targeting channel-centered network architectures rather than isolated channels alone, highlighting ion channels as structural components of broader signalling systems rather than solely bioelectrical regulators.

## 1. Introduction

Tumorigenesis and progression of cancer are driven by the progressive alterations in signalling networks, metabolic state, and cellular organization that control proliferation, invasion, and therapy resistance. At the same time, as the investigation of these processes is typically undertaken through the utilization of transcriptomic or pathway-centric approaches, mounting evidence suggests that malignant phenotypes can also emerge from altered cellular organization at the level of protein–protein interactions (PPIs).

In this context, ion channels have attracted increasing interest in cancer biology due to their ability to integrate bioelectric, biochemical, and mechanical signals [1,2,3,4,5]. Among these, K^+^ channels represent the largest and most diverse family, and numerous studies have linked dysregulated expression or activity of multiple K^+^ channel subtypes to key hallmarks of malignancy, including tumor growth, migration, metabolic adaptation, and therapy resistance across a broad range of cancers [6,7,8].

Traditionally, the contribution of K^+^ channels to tumor biology has been interpreted as a direct consequence of their ion-conducting function, through effects on membrane polarization and thereby setting driving force Ca^2+^ flux, adjusting cellular volume homeostasis, and signal-dependent transcriptional regulation [9]. Nonetheless, accumulating evidence indicates that K^+^ channels can also exert non-conductive functions, engaging in physical and functional interactions with signalling proteins, scaffolds, trafficking regulators, and cytoskeletal components, thereby directly contributing to the spatial organization of oncogenic signalling networks [10,11,12,13,14,15,16,17,18,19,20,21]. Recent advances in proximity proteomics and targeted biochemical approaches have revealed that the environments of K^+^ channel-associated proteins are characterized by high levels of structural complexity and compartmentalization. These environments extend across the plasma membrane, the mitochondria, and the endomembrane system [15,16,17,18,19,20,21].

Although substantial progress has been achieved in reconstructing individual K^+^ channel interactomes using proximity proteomics and affinity-based approaches, these datasets were generated across distinct biological systems and experimental platforms, including BioID, TurboID, APEX2, AP-MS, and co-immunoprecipitation methodologies [13,14,15,16,17,18,19,20,21]. While this methodological diversity expands the range of detectable channel-associated interactions and biological contexts, it complicates direct comparison across channel families. Consequently, it remains unclear whether cancer-relevant channel functions arise primarily from channel-specific molecular interactions or from recurrent higher-order organizational principles emerging across independently reconstructed interactomes. Addressing this question is essential for defining the functional relevance of K^+^ channels beyond descriptive interactome catalogues.

To address this knowledge gap, a comparative interactome-based framework for K^+^ channels is proposed, relying exclusively on PPI and proximity labeling datasets that are supported by experiments. In contrast to classical co-immunoprecipitation (co-IP), PPIs defined by proximity labeling can also capture weak and transient interactions in the natural environment of the channel without extracting them from the membrane with detergents. Therefore, we focus only on the cancer-relevant K^+^ channels whose intact cell interactomes have recently been defined, especially the voltage-gated Shaker channel Kv1.3, the leak channel TASK1, the intermediate conductance calcium-activated K^+^ channels KCa3.1, the inward rectifying Kir2.1, and the voltage-gated Kv11.1, also named hERG, and the inward rectifying Kir1.1, also named ROMK [15,16,17,18,19,20,21,22,23]. In particular, Kv1.3 is overexpressed in several cancer types [24,25,26] and genetic proof demonstrated its crucial contribution to tumor progression in melanoma [21], lung cancer [27], as silencing of the channel drastically reduced tumor growth in both cancer types. In addition to the plasma membrane (PM), Kv1.3 is also functional in the mitochondrial inner membrane (IMM) [28,29,30], where it regulates mitochondrial function [31] and its specific inhibition by mitochondria-targeted Kv1.3 inhibitors triggers apoptosis in various cancer types [32,33,34]. Similarly to Kv1.3, KCa3.1 has a dual localization to PM and IMM [35,36,37] and plays a crucial role in cancer cell proliferation, migration and metastatic spread [38,39,40,41,42]. Another widely studied channel in the context of cancer is hERG [43], whose inhibition led to apoptosis in glioblastoma cells [44]. Instead in non-small cell lung cancer (NSCLC), activation of this channel, known to interact with integrins [45], led to a reduction in tumor growth [46]. TASK-1 is a two-pore leak channel, whose low expression correlated with poor prognosis in lung adenocarcinoma [47]. Specific inhibition of TASK-1 instead was reported to reduce breast cancer cell proliferation [48]; consistently, its genetic abrogation induced apoptosis and reduced proliferation [49]. Regarding Kir channels, blockade of Kir2.1 in breast cancer cells can overcome chemoresistance [50] and its high expression was proposed to be a reliable prognostic marker in medulloblastoma [51]. Silencing Kir2.1 reduced the invasiveness and metastatic spread as well as the Epithelial–Mesenchymal Transition (EMT) of gastric cancer cells [52]. Kir1.1 is a biomarker of Renal Cell Carcinoma (RCC) [53] and its re-expression in these cells decreased proliferation and invasion [23].

Collectively, these observations indicate that K^+^ channels can influence tumor behavior through multiple mechanisms and across diverse biological contexts. However, the same channel may exert distinct or even opposing effects depending on the tumor type, suggesting that channel-associated interaction networks are strongly context-dependent.

These observations raise a central question: whether K^+^ channel-associated cancer functions emerge from conserved molecular interactors shared across channels or from partially convergent higher-order organizational principles involving membrane trafficking, spatial compartmentalization, junctional organization, and signaling architecture. The objective of this study was therefore to systematically compare experimentally supported K^+^ channel interactomes to determine whether functional convergence across K^+^ channels emerges primarily from shared molecular interactors or from partially convergent higher-order interactome architectures.

## 2. Results and Discussion

### 2.1. Pan-Cancer Gene-Level Analyses Reveal Heterogeneous and Context-Dependent K^+^ Channel Regulation

Before investigating interactome organization, we first asked whether cancer-associated K^+^ channel functions could simply be explained at the gene-expression or mutational level across tumor types. If a conserved pan-cancer K^+^ channel program existed, one would expect coherent expression, co-expression, or mutational patterns across malignancies.

**Figure 1:**
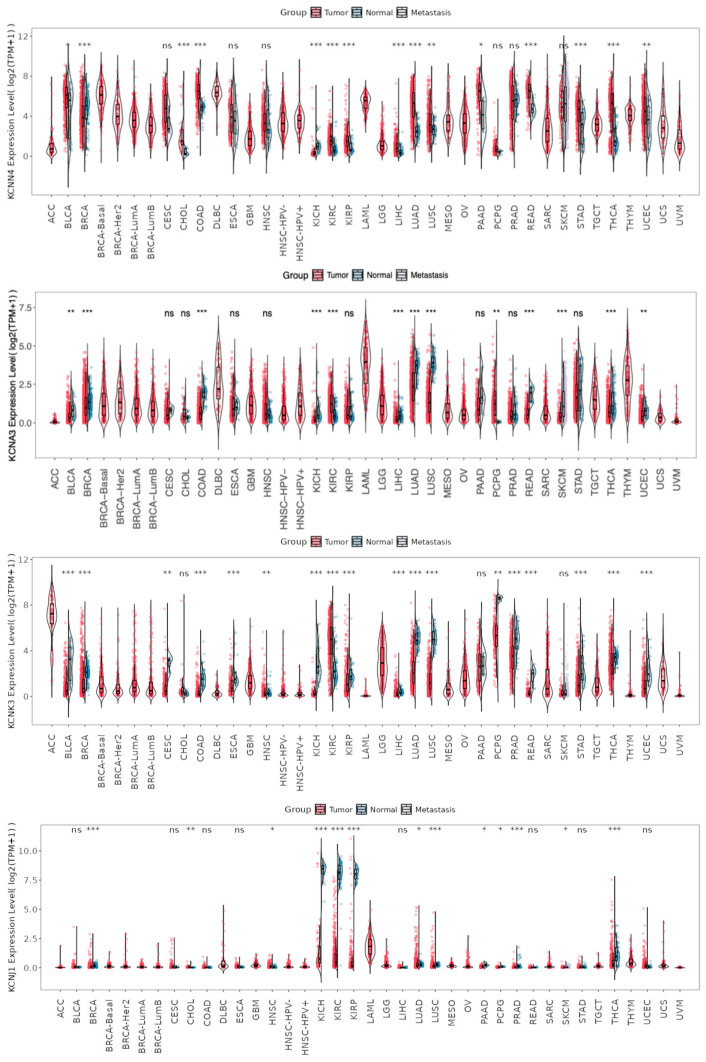

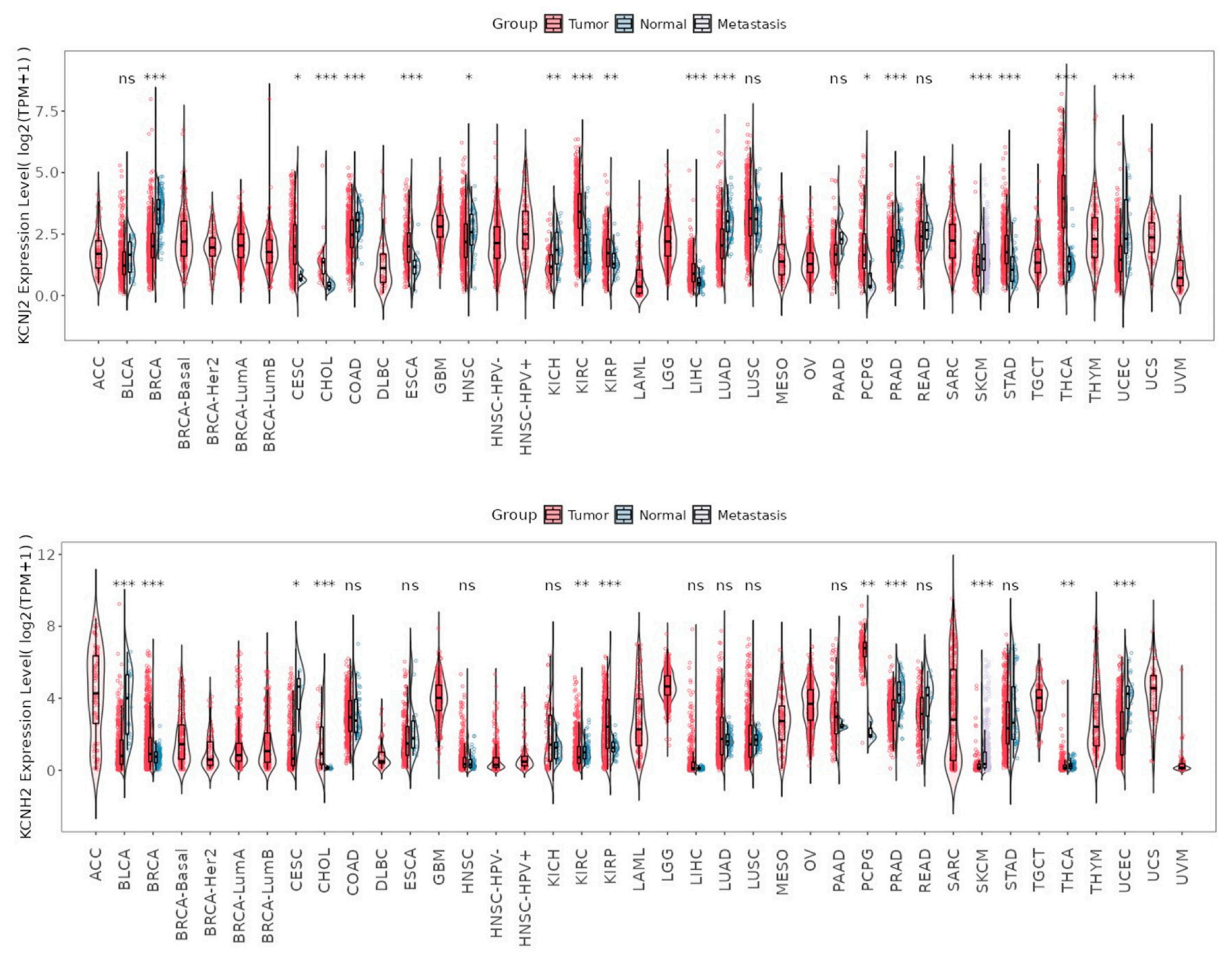
The expression landscape of KCa3.1, Kv1.3, TASK-1, Kir2.1 and hERG across human cancers. Violin plots showing the mRNA expression levels (log2(TPM + 1)) of KCa3.1 (*KCNN4*), Kv1.3 (*KCNA3*), TASK-1 (*KCNK3*), Kir2.1 (*KCNJ2*) and hERG (*KCNH2*) in tumour, normal, and metastatic samples across multiple TCGA cancer types. The significance of differences between groups is indicated using labels (ns, not significant; * *p* < 0.05; ** *p* < 0.01; *** *p* < 0.001).

**Figure 2:**
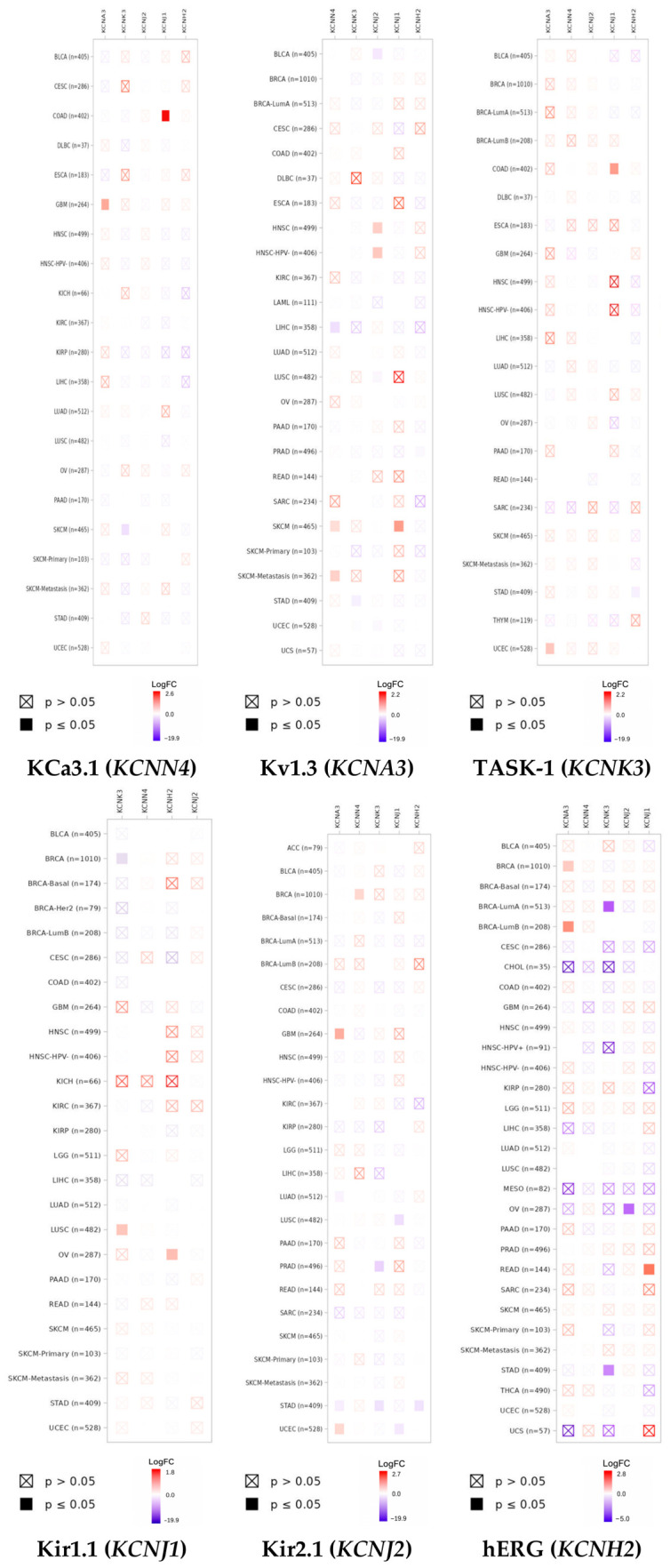
Co-expression patterns of cancer-associated K^+^ channel genes across TCGA tumour types. Heatmaps show Spearman correlation coefficients between channel pairs across tumour cohorts. Red indicates positive correlation and blue indicates negative correlation. Crossed cells indicate non-significant correlations (*p* > 0.05), whereas filled circles denote statistically significant correlations (*p* < 0.05).

**Figure 3:**
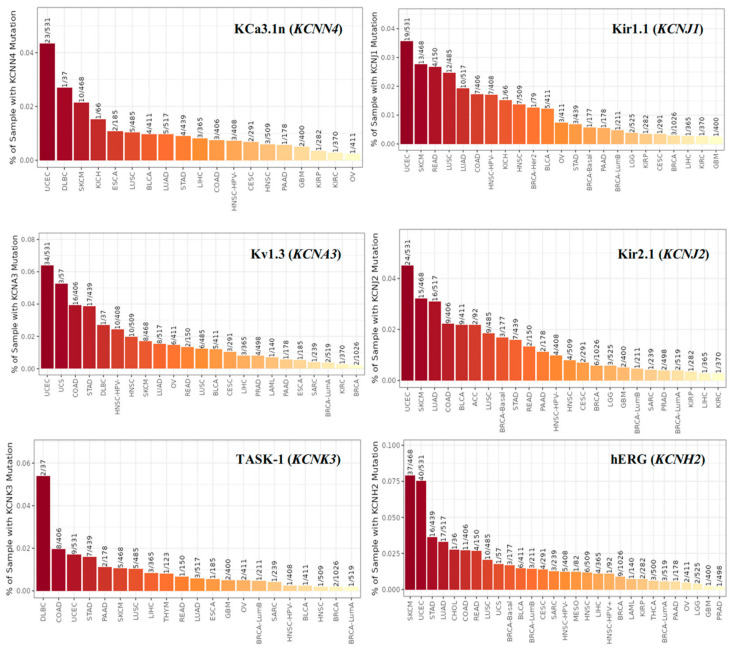
Somatic mutation frequencies of cancer-associated K^+^ channel genes across TCGA tumour types. Somatic mutation frequencies were assessed using the TIMER3 Gene_Mutation module across TCGA cancer cohorts. Bar plots show the proportion of tumour samples harbouring mutations in KCa3.1 (KCNN4), Kir1.1 (KCNJ1), Kv1.3 (KCNA3), Kir2.1 (KCNJ2), TASK-1 (KCNK3), and Kv11.1/hERG (KCNH2). Tumour types are ordered according to decreasing mutation frequency for each gene. Mutation frequencies are displayed as proportions of mutated samples, as reported by the TIMER3 platform. The analyses reveal substantial variability in mutation prevalence across tumour types and channel families, indicating heterogeneous patterns of genetic alteration among K^+^ channel genes in human cancers.

Comparative analyses of tumor-normal transcriptomic datasets revealed marked heterogeneity in expression patterns of the six channels examined in this study across cancer types (Figure 1). Additional co-expression and mutational analyses further supported the absence of a unified pan-cancer K^+^ channel program and are provided in Figure 2 and Figure 3.

Collectively, these findings argue against the existence of a unified pan-cancer K^+^ channel program at the gene level. Instead, any functional convergence observed across channels is more likely to emerge from higher-order spatial and organizational properties rather than from conserved expression or mutational patterns alone. These observations led us to investigate whether protein interaction architectures could provide a more informative framework for understanding novel functions of cancer-associated K^+^ channels.

### 2.2. Comparative Interactome Analysis Defines Distinct Organizational Paradigms Across K^+^ Channel Families

**Figure 4:**
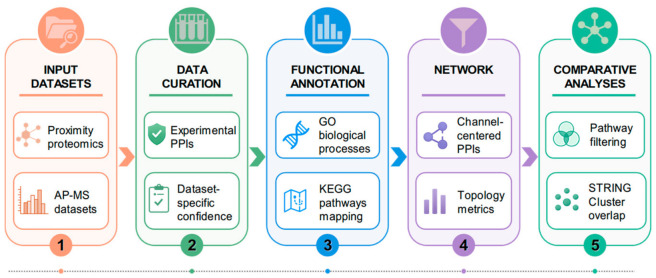
Integrated analysis of K^+^ channel interactomes. Integrated workflow for interactome reconstruction and comparative analysis. Proximity proteomics and AP-MS datasets were curated to retain only experimentally supported PPI using dataset-specific confidence thresholds. Channel-centred interactomes were reconstructed, functionally annotated, and analysed using network topology metrics and pathway-based approaches, enabling pairwise and multi-channel intersection analyses and identification of convergent, cancer-relevant interaction modules.

To facilitate direct comparison across the examined K^+^ channels, we assembled published experiment-based proximity-labelling, affinity-purification and co-immunoprecipitation datasets and harmonized them through a unified network analysis pipeline [16,17,18,19,20,21] (Figure 4). In addition, we report here for the first time the complete Kv1.3 interactome obtained by co-immunoprecipitation coupled to mass spectrometry, generated in the context of our previous work on the mitochondrial routing of Kv1.3 and originally used to identify the molecular machinery involved in its mitochondrial trafficking [22]. This dataset includes the full list of Kv1.3-associated proteins and expands substantially upon the subset of interactors discussed in the original study. This comparative framework enabled systematic analysis of higher-order interactome architecture, including network topology, compartmentalization, modularity, and integration with signaling and trafficking systems across channels profiled in distinct biological and experimental contexts.

To avoid overinterpretation of interactome data, we distinguished three levels of evidence throughout the analysis. First, experimentally validated functional interactions refer to cases in which the original studies directly demonstrated an effect of a given interactor or module on channel activity, localization, signaling output, or cellular phenotype. Second, proximity-derived associations refer to proteins identified by BioID, TurboID, or APEX-based proximity labeling, which indicate spatial proximity to the channel but do not necessarily imply direct physical binding or functional regulation. Third, architecture-level inferences refer to higher-order conclusions derived from network topology, subcellular localization, pathway enrichment, and overlap analyses across datasets. These inferences are therefore interpreted as organizational frameworks rather than direct evidence of individual functional interactions.

**Figure 5:**
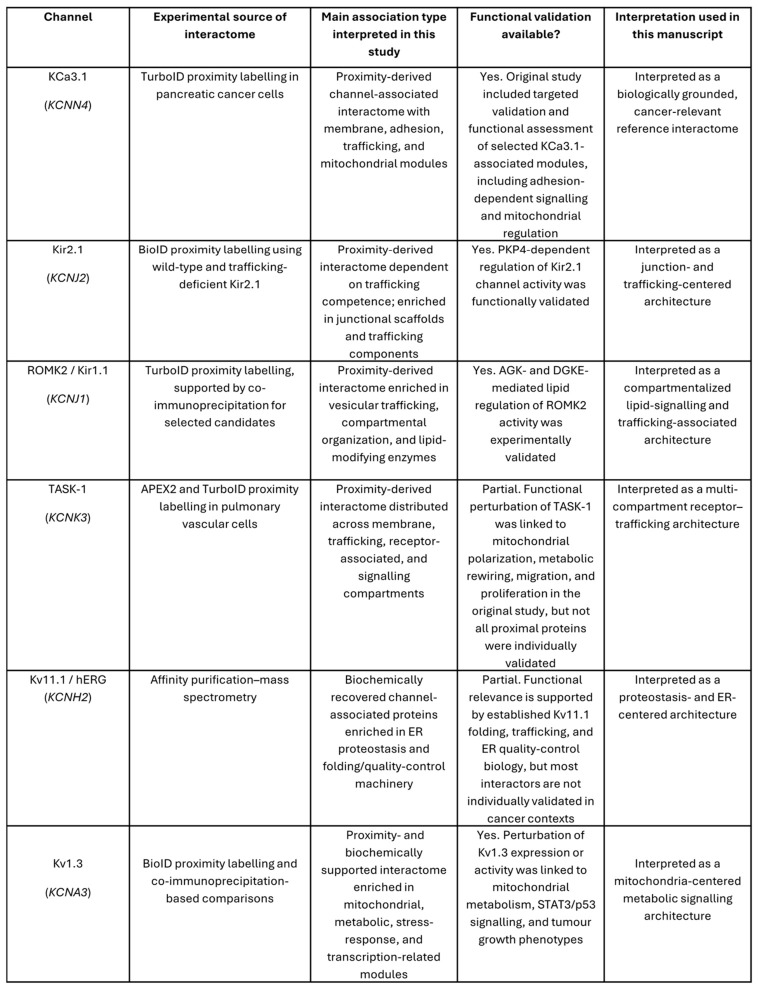
Conceptual framework and evidence supporting the comparative analysis of K^+^ channel interactomes. Summary of the potassium channel interactomes included in this study, reporting the experimental approach used for interactome identification, the predominant architectural features emerging from network analyses, the degree of functional validation available in the original studies, and the interpretative framework adopted in the present work. KCa3.1 (KCNN4) was selected as the reference interactome because it was generated directly in cancer cells and supported by extensive functional validation. Comparative analysis revealed distinct but recurrent organizational architectures, including membrane-proximal signalling and adhesion networks (KCa3.1), junction- and trafficking-centered platforms (Kir2.1), compartmentalized lipid-signalling and trafficking modules (ROMK2/Kir1.1), multi-compartment receptor-trafficking architectures (TASK-1), proteostasis- and ER-centered networks (Kv11.1/hERG), and mitochondria-centered metabolic signalling architectures (Kv1.3). Together, these interactomes support the concept that functional convergence among K^+^ channels is better explained by shared architectural principles than by extensive overlap of individual interactors.

This classification emphasizes that proximity-labelling hits were not interpreted as direct functional interactions unless supported by functional validation in the original studies. For shared interactomes, functional interpretation was assigned at the module or architecture level only when supported by coherent network topology, subcellular localization bias, and pathway organization. The interpretative framework adopted throughout this study is summarized in Figure 5.

**Figure 6:**
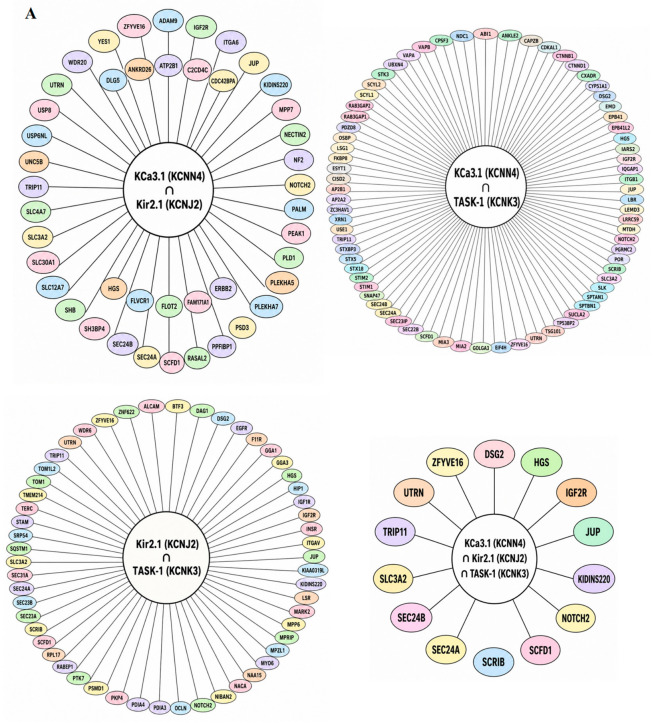

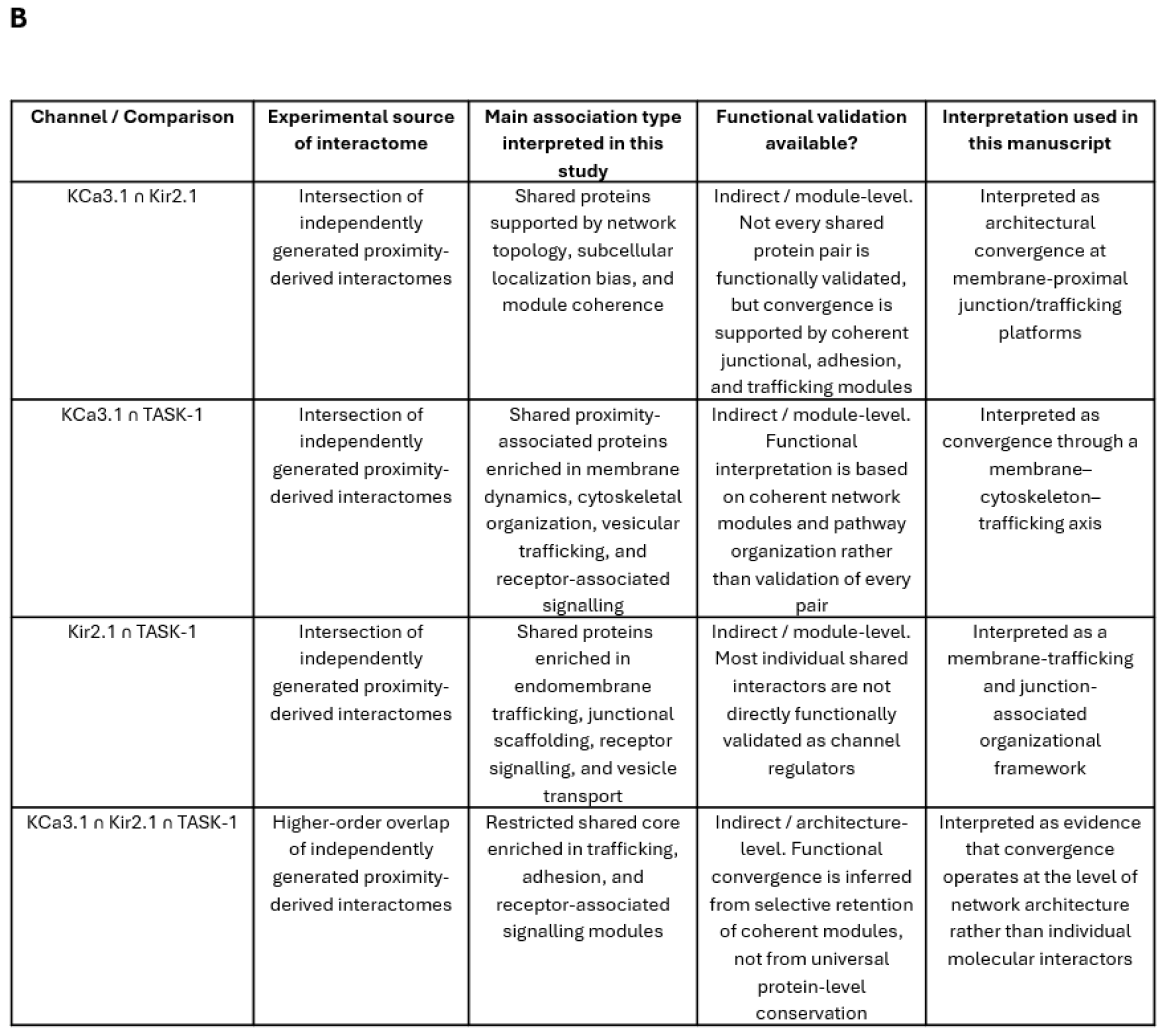
Comparative analysis of shared interactome architectures among KCa3.1, Kir2.1, and TASK-1. (**A**) Network representation of the pairwise and higher-order intersections among the proximity-derived interactomes of KCa3.1 (KCNN4), Kir2.1 (KCNJ2), and TASK-1 (KCNK3). The upper left panel shows proteins shared between KCa3.1 and Kir2.1, the upper right panel proteins shared between KCa3.1 and TASK-1, and the lower left panel proteins shared between Kir2.1 and TASK-1. The lower right panel highlights the restricted core of proteins common to all three channel interactomes. Nodes represent shared proteins identified in the respective overlap analyses. Colors are used only to facilitate visualization and do not indicate functional categories or quantitative parameters. (**B**) Summary of the pairwise and higher-order interactome intersections analyzed in this study. For each overlap, the figure reports the origin of the compared datasets, the predominant functional and topological features emerging from the shared protein networks, the level of functional validation available, and the interpretative framework adopted in the present manuscript. Although the intersecting interactomes contain relatively limited numbers of common proteins, network reconstruction and enrichment analyses revealed recurrent modules associated with membrane trafficking, junctional organization, cytoskeletal dynamics, adhesion complexes, and receptor-associated signalling. The KCa3.1–Kir2.1 overlap highlights convergence within membrane-proximal junctional and trafficking platforms, whereas the KCa3.1–TASK-1 intersection emphasizes a membrane–cytoskeleton–trafficking axis. The Kir2.1–TASK-1 overlap identifies an integrated framework linking endomembrane trafficking, junctional scaffolding, and receptor signalling. The higher-order intersection among KCa3.1, Kir2.1, and TASK-1 reveals a restricted but coherent shared core, supporting the concept that functional convergence among K^+^ channels is primarily encoded at the level of network architecture and module organization rather than through extensive conservation of individual molecular interactors.

Across datasets, a limited number of recurring organizational paradigms were observed, ranging from membrane-proximal, signalling-integrated scaffolds to organelle-centered bottlenecks and trafficking-restricted networks (Figure 5 and Figure 6). In the following sections, these paradigms are illustrated, with emphasis on their potential relevance to cancer-associated signalling.

For the comparative analysis, KCa3.1 (*KCNN4*) was selected as a reference framework (Figure 5; Table 1). In contrast to the majority of available K^+^ channel interactomes, which have been defined upon expression of the channels in heterologous systems such as HEK293-cells, the KCa3.1 interactome was elucidated directly in a cancer cell [17]. Using TurboID-based proximity labeling in KPC pancreatic ductal adenocarcinoma (PDAC) cells harboring KRAS and p53 mutations, 283 KCa3.1-proximal proteins were identified and some of the most interesting hits were subsequently subjected to targeted validation and functional assessment [17]. This dataset therefore provides a biologically grounded and disease-relevant view of channel-centered signaling organization.

Functional annotation revealed that the KCa3.1 interactome is not primarily enriched in ion transport machinery but rather in cell adhesion complexes, actin cytoskeleton regulators, small GTPase signalling pathways, and membrane trafficking components, defining a predominantly, membrane-associated signalling architecture [17]. Subcellular mapping further indicated preferential localization of KCa3.1-associated proteins at cell–cell junctions, focal adhesions, membrane rafts, and leading-edge structures, consistent with established roles of KCa3.1 in cytoskeletal remodelling, cell migration, and invasive behaviour in cancer cells [17].

The KCa3.1 interactome also illustrates the distinction between proximity-derived associations and experimentally supported functional interactions introduced above. While TurboID labeling identified proteins located within the spatial environment of the channel, several modules and interactors were subsequently linked to functional signaling outputs through targeted experimental analyses [17].

Network topology analysis revealed a distributed and modular organization suggesting that KCa3.1 functions as a Ca^2+^-anchored signalling scaffold coordinating multiple peripheral modules. Interactions with STIM1 and integrin β4 (ITGB4) functionally connect this architecture to Ca^2+^ entry and adhesion-dependent signalling pathways implicated in pancreatic cancer progression and aggressiveness. Concurrently, a subset of KCa3.1-associated proteins, spatially segregated and localized to mitochondria, supports the presence of an additional compartmentalized interactome component linked to metabolic fitness and oxidative stress. This finding is in accordance with previous reports of mitochondrial KCa3.1 localization and function [17,36].

Collectively, these characteristics establish KCa3.1 as a biologically and experimentally grounded reference interactome, thereby providing a robust organizational baseline for identifying convergent and divergent signalling architectures across distinct the examined K^+^ channel belonging to different families (Table 1). Notably, this experimentally validated dataset provides a biologically grounded reference framework against which independently reconstructed channel-centered interactomes can be comparatively evaluated.

### 2.3. Architectural Convergence and Divergence Between KCa3.1, Kir2.1 and Kir1.1

The interactome of the inward rectifier channel Kir2.1 (*KCNJ2*) was examined as a representative example of a junction- and trafficking-centered organizational paradigm. The Kir2.1 interactome, identified by BioID-based proximity labelling using both wild-type Kir2.1 and the trafficking-deficient Andersen-Tawil mutant Kir2.1Δ314-315 [18], comprised 218 high-confidence interactors, whose composition was strongly dependent on trafficking competence. These proteins therefore represent channel-associated proximity networks rather than exclusively direct physical binding partners.

Consistent with the interpretative framework introduced above, the Kir2.1 dataset primarily defines a proximity-derived interactome rather than a comprehensive map of experimentally validated functional interactions. Nevertheless, several organizational modules identified within this network were subsequently linked to experimentally supported functional outputs. In particular, functional analysis of the junctional scaffold protein PKP4 demonstrated that this architectural organization modulates Kir2.1 channel activity [18].

Network reconstitution revealed a junction- and scaffold-centered architecture, enriched for adhesion complexes, PDZ-domain-containing proteins, and COPII-mediated trafficking components. In contrast, loss of membrane targeting shifted the interactome toward intracellular trafficking and quality-control pathways, indicating that membrane localization strongly influences the structural organization of the Kir2.1-associated protein environment.

**Figure 7:**
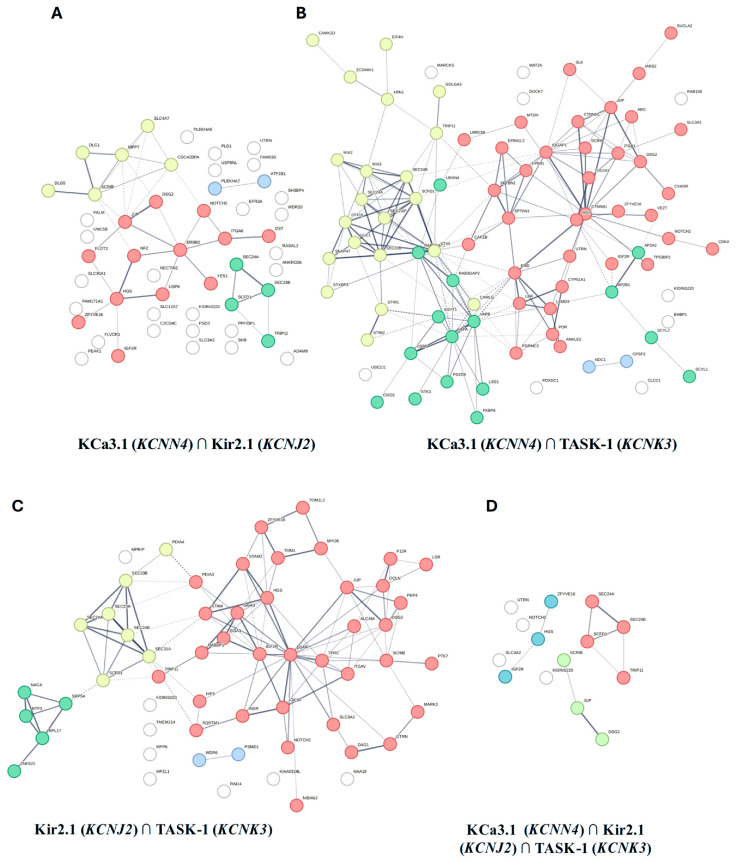
Pathway-level refinement and modular organisation of shared potassium channel interactomes. STRING-based interaction networks combined with pathway enrichment analyses were used to resolve the functional architecture of shared interactomes across potassium channel families. Networks derived from pairwise intersections of KCa3.1 (*KCNN4*)—Kir2.1 (*KCNJ2*) (**A**), KCa3.1 (*KCNN4*)—TASK-1 (*KCNK3*) (**B**), and Kir2.1 (*KCNJ2*)—TASK-1 (*KCNK3*) (**C**) reveal recurrent, highly interconnected modules despite differences in channel identity. Across all pairwise combinations, shared interactors preferentially organise into discrete clusters associated with junctional organisation, endocytic sorting, and receptor-associated signalling, rather than forming diffuse or functionally mixed assemblies. Although channel-specific peripheral nodes are retained, the core modular architecture is conserved across combinations. (**D**) The triple-channel intersection (KCa3.1 (*KCNN4*)—Kir2.1 (*KCNJ2*)—TASK-1 (*KCNK3*)) exhibits a marked reduction in network size and complexity, with selective retention of a restricted subset of highly connected hubs embedded within junction- and receptor-associated signalling modules. Peripheral nodes and broadly acting cellular maintenance components are progressively lost, indicating increased functional stringency with higher-order overlap. Together, these analyses demonstrate that convergence across potassium channel interactomes is hierarchically structured and emerges through selective reuse of spatially organised signalling and trafficking modules, providing a mechanistic framework for interpreting higher-order overlap. Node colours indicate dominant functional module assignment based on STRING clustering and pathway enrichment analyses: Red—receptor-associated and growth factor signalling components (e.g., EGFR/IGF receptor-associated proteins, integrins, Notch-related signalling nodes); Yellow—junctional scaffolds and polarity-related proteins (e.g., adherens and desmosomal junction components, polarity complexes); Green—endocytic and vesicle-mediated trafficking machinery (e.g., COPII components, vesicle tethering and trafficking regulators); Light blue—endosomal sorting and receptor trafficking adaptors (e.g., ESCRT-related proteins and early endosomal signalling adaptors); Grey/white—peripheral or weakly connected nodes (proteins not assigned to dominant functional modules or with low network connectivity). Edges represent STRING-derived protein–protein associations. Solid lines indicate established/retained associations among proteins in the network, whereas dashed lines indicate weaker or putative associations. The thickness and opacity of the solid lines are proportional to the combined STRING confidence score, with thicker/darker lines representing stronger evidence for the association.

Intersection analysis between KCa3.1 and Kir2.1 interactomes identified a densely interconnected shared network predominantly organized near the plasma membrane and associated with sites of cell–cell contact (Figure 7A). Crucially, overlap in Venn or intersection analyses was not interpreted as direct evidence of functional interaction between individual proteins; rather, shared protein sets were evaluated as architecture-level signals whose biological relevance required support from network connectivity, localization bias, and enrichment of functionally related modules. Convergence in this context is not inferred from pathway enrichment alone, but from the recurrent combination of network topology, subcellular localization patterns, and modular organization observed across independently reconstructed interactomes.

This integrative framework allows higher-order organizational similarities to be identified even in the absence of extensive overlap at the level of individual molecular interactors. Shared interactors form a densely connected architecture enriched in adhesion complexes and junctional scaffolds, supporting a module-level interpretation of local architectural convergence within this channel pair (Figure 7A).

This convergence should not be interpreted as evidence for a universal pan-cancer signaling module shared across all K^+^ channels. Rather, it reflects a local and context-dependent architectural convergence between this specific channel pair. The application of modular analysis within this network identified three major organizational modules. A dominant module was enriched in canonical determinants of junctional organization and polarity, including the SCRIB–DLG scaffolds, MPP7, NF2, and JUP, consistent with a structural role at adherens junctions. A second module comprised adhesion- and receptor-associated proteins (ERBB2, ITGA6, NECTIN2, NOTCH2), positioning oncogenic signalling nodes within a junctional membrane context. A third module was associated with components of endocytosis and vesicular trafficking (HGS, USP8, PSD3, ZFYVE16, IGF2R), suggesting functional coupling between junctional architecture and intracellular transport pathways.

**Figure 8:**
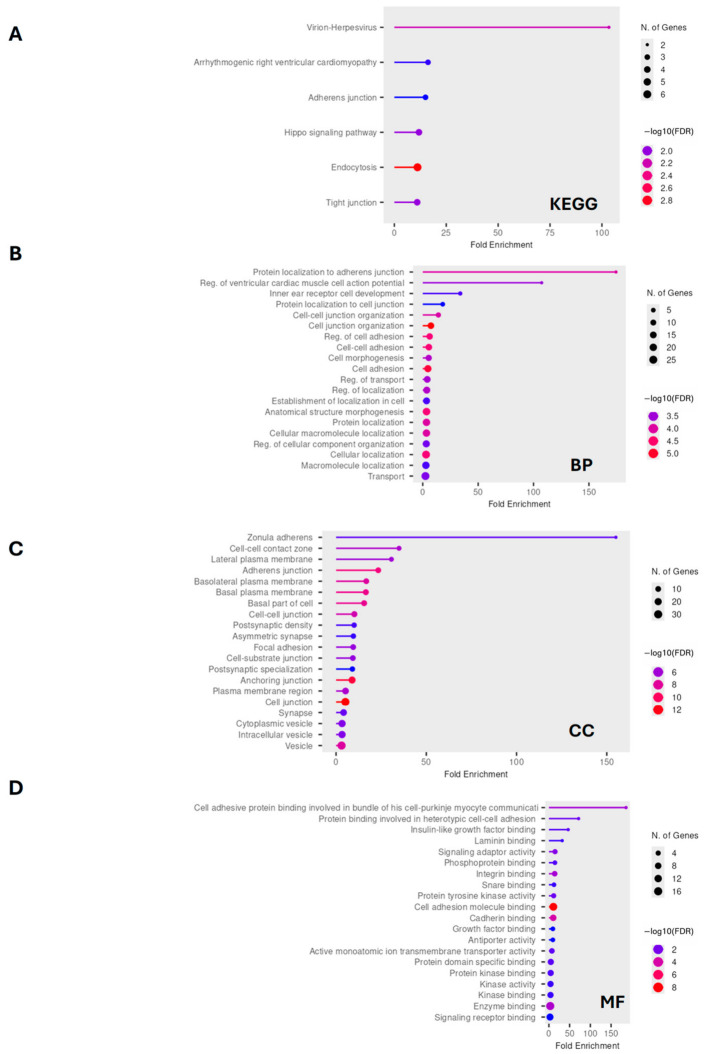
Functional enrichment analysis of the shared KCa3.1-Kir2.1 interactome highlights a conserved membrane-proximal, junction-associated architecture. (**A**) KEGG pathway enrichment analysis of proteins shared between the KCa3.1 and Kir2.1 interactomes. Dot plots show significantly enriched pathways ranked by fold enrichment. Point size reflects the number of genes associated with each pathway, while color indicates statistical significance expressed as −log10(FDR). Endocytosis emerges as the most significantly enriched pathway, followed by adherens junctions, tight junctions, and Hippo signaling, indicating preferential involvement of the shared interactome in membrane trafficking and contact-dependent signaling pathways. (**B**) Gene Ontology (GO) Biological Process enrichment analysis of the shared interactome. Enriched terms are predominantly associated with protein localization to cell–cell junctions, organization of adherens junctions, regulation of cell adhesion, and control of cell morphology, supporting a model in which shared interactors function in the spatial organization of membrane-associated signaling platforms. (**C**) GO Cellular Component enrichment analysis. Shared interactors display a strong localization bias toward junctional and membrane-associated compartments, including zonula adherens, lateral plasma membrane, focal adhesions, and cell–substrate junctions, indicating that the overlap between KCa3.1 and Kir2.1 is spatially constrained to specialized membrane domains. (**D**) GO Molecular Function enrichment analysis. The most significantly enriched categories include protein binding, cadherin binding, integrin binding, kinase binding, and adaptor/scaffold activity. This enrichment profile indicates that the shared interactome is dominated by organizational and scaffolding proteins rather than catalytic enzymes, consistent with a role in higher-order signal integration rather than direct ion transport. Abbreviations: KEGG, Kyoto Encyclopedia of Genes and Genomes; GO-BP, Gene Ontology Biological Process; GO-CC, Gene Ontology Cellular Component; GO-MF, Gene Ontology Molecular Function; FDR, false discovery rate.

Functional enrichment analyses provided additional support for this organizational interpretation. The most significantly enriched Kyoto Encyclopedia of Genes and Genomes (KEGG) pathways included endocytosis, adherens junctions, tight junctions, and the Hippo signalling pathway, highlighting a tight integration between membrane trafficking and junction-associated signalling platforms (Figure 8A; Supplementary Figure S1A–D). For clarity, a glossary of abbreviations, pathway terminology, Gene Ontology categories, and selected protein symbols used throughout the supplementary analyses is provided in Supplementary Table S1. Gene Ontology (GO) analysis further reinforced this interpretation. Biological Process (BP) enrichment revealed over-representation of terms associated with cell–cell junction organization, protein localization to adherens junctions, and regulation of cell adhesion and morphology (Figure 8B; Supplementary Table S2). Cellular component (CC) analysis indicated a strong localization bias towards membrane-associated and junction-associated compartments, including the zonula adherens, lateral plasma membrane and focal adhesions (Figure 8C; Supplementary Table S3). At the molecular function (MF) level, the most enriched terms included binding activities and signalling adaptor functions, supporting the interpretation that many shared interactors primarily act as scaffolds or organizational nodes rather than catalytic effectors (Figure 8D; Supplementary Table S4).

These enrichment analyses should be interpreted as higher-order architectural and organizational relationships rather than direct evidence of functional regulation for every individual interactor identified within the overlap network. Collectively, the analyses support a model in which the shared KCa3.1-Kir2.1 interactome contributes to the spatial organization of membrane-associated signalling platforms integrating junctional architecture, membrane trafficking, and oncogenic signalling processes.

To assess whether a junction-centered interactome that was clearly observed for Kir2.1 represents a general feature of inward rectifier K^+^ channels, we examined the interactome of ROMK2 (Kir1.1/KCNJ1), defined using TurboID-based proximity labelling [19]. In contrast to Kir2.1, which displays a pronounced enrichment in junction-associated scaffolds and membrane-proximal signalling modules in our analyses, ROMK2 has been reported to localize across multiple membrane compartments, including the plasma membrane, ER, vesicular structures, and mitochondria [19]. This broader subcellular distribution suggests a distinct organizational framework compared to the more junction-focused architecture observed for Kir2.1.

Consistent with this distributed localization, proximity labelling identified a ROMK2-associated proxisome enriched in proteins involved in vesicular trafficking, membrane remodelling, and intracellular transport, with comparatively limited representation of junctional scaffolds, polarity complexes, or adhesion-associated proteins. Network reconstruction revealed associations with clathrin-dependent endocytosis, vesicle-associated cytoskeletal components, endoplasmic reticulum transport machinery, and chaperone systems. Together, these observations are consistent with an interactome architecture shaped predominantly by trafficking dynamics and compartmental organization rather than by stable membrane-proximal scaffolding [19]. A distinguishing feature of the ROMK2 interactome was the presence of lipid-modifying enzymes, most prominently acylglycerol kinase (AGK) and diacylglycerol kinase ε (DGKE), whose interactions were validated experimentally. Functional assays demonstrated that the lipid products generated by AGK- and DGKE-mediated reactions directly modulate ROMK2 channel activity, identifying local lipid synthesis as a central functional regulatory axis within this interactome [19].

Collectively, these results delineate for ROMK2 (Kir1.1) an interactome lacking a coherent membrane-proximal signalling core instead revealing an organized compartment-restricted, lipid-regulated logic. In sharp contrast to Kir2.1, whose interactome is structured around junctional complexes and membrane stability, ROMK2 emerges as a channel whose signalling functions are encoded primarily through compartmentalization rather than junctional integration.

Consistent with this architectural divergence, TCGA analyses revealed heterogeneous expression and mutational profiles among inward rectifier K^+^ channels across tumor types, with limited correlation between Kir2.1 (KCNJ2) and other Kir family members (Figure 1, Figure 2 and Figure 3, Supplementary Figure S2). These observations further support the notion that distinct channel-specific regulatory architectures, rather than conserved gene-level programs, underlie the functional diversity of inward rectifier K^+^ channels in cancer.

### 2.4. Architectural Convergence and Divergence Between KCa3.1 and TASK1

To extend the comparative analysis to two-pore domain K^+^ channels, we examined TASK-1 (*KCNK3*), whose proximity interactome was defined using complementary APEX2- and TurboID-based approaches in pulmonary vascular cells [16]. Across diverse platforms, KCNK3-associated proteins were found to be distributed across multiple compartments [16]. In accordance with this organization, the KCNK3 interactome is enriched in regulators of membrane routing, recycling, and degradation, including ER-to-Golgi transport, endocytosis, and vesicle-mediated trafficking components, and incorporates multiple signalling nodes such as Src family kinases, ROCK1/2, EGFR/IGF1R-associated modules, and PI3K/AKT-linked pathways [16]. From a functional perspective, the perturbation of TASK-1 in vascular cells has been associated with altered mitochondrial polarization, metabolic rewiring, and impaired migratory and proliferative responses, particularly in endothelial contexts [16]. Collectively, these observations suggested that TASK-1 does not define a single dominant scaffold or a discrete organelle-centered hub, but rather operates within a multi-compartment, membrane-proximal interactome that bridges plasma membrane microdomains. This positions TASK-1 as an intermediate “bridge architecture” within the broader landscape of K^+^ channel interactomes [16].

We next asked whether similar organizational principles are shared with the Ca^2+^-activated K^+^ channel KCa3.1. This investigation involved the analysis of the intersection of their associated protein networks. PPI analysis of the KCa3.1 against TASK-1 (*KCNN4* ∩ *KCNK3*) shared gene set revealed the emergence of a highly connected and non-random network architecture, as visualized by STRING analysis. The resulting network is characterized by a dense core of interacting proteins and multiple highly connected hub nodes, including HGS, EPB41, EPB41L2, IQGAP1, CTNNB1, CTNND1, and spectrin family members. These are known regulators of cytoskeletal organization, membrane stability, and signal integration at the plasma membrane (Figure 7B). The prevalence of these hubs suggests that the overlap between KCa3.1 and TASK-1 is organized around membrane-associated signalling, trafficking, and structural coordination rather than around shared ion-conducting functions. Most of the common nodes are adaptor, scaffolding, or membrane-organizing proteins known to coordinate cytoskeletal dynamics, membrane stability, and receptor-associated signalling. Although these observations support the existence of a common architectural framework, the functional relevance of individual interactions remains variable and is experimentally established only for a subset of the identified proteins. Therefore, the shared network should be interpreted primarily as a map of organizational convergence rather than as direct evidence of functional coupling between all overlapping interactors.

To determine whether the shared proteins were organized into biologically interpretable modules, we performed STRING-based clustering analysis of the KCa3.1-TASK-1 overlap. This approach was applied to distinguish broad architectural associations from more coherent functional groupings and to identify whether the shared interactome was dominated by specific cellular processes. The analysis revealed multiple interconnected but functionally distinct modules (Figure 7B; Table 2). STRING-based k-means clustering grouped the shared proteins into coherent functional clusters associated with trafficking, cytoskeletal organization, membrane dynamics, and receptor-associated signalling. The protein composition and functional annotation of each cluster are summarized in Table 2.

One prominent cluster is enriched in proteins involved in vesicular trafficking and membrane transport, including components of the COPII and SNARE machineries (e.g., SEC23/24, SEC22B, USE1, SNAP47), as well as regulators of endosomal sorting and recycling such as TSG101 and RAB3GAP2.

**Figure 9:**
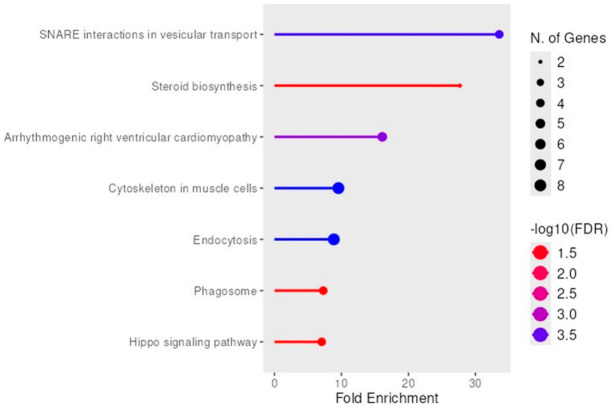
KEGG pathway enrichment of the shared KCa3.1-TASK-1 interactome identifies vesicle trafficking and membrane dynamics as dominant functional axes. KEGG enrichment dot plot of proteins shared between KCa3.1 and TASk-1 interactomes. SNARE-mediated vesicular transport represents the most significantly enriched category, followed by endocytosis, cytoskeleton-associated pathways, and Hippo signaling. Dot size corresponds to gene count, while color denotes −log10(FDR). These results indicate that functional convergence between KCa3.1 and TASK-1 is primarily embedded within a membrane-cytoskeleton-trafficking axis rather than ion conduction-specific pathways.

A second cluster, derived from the same KCa3.1-TASK-1 overlap, is represented by proteins implicated in cytoskeletal dynamics, cell–cell junctions, and membrane anchoring, including catenins, integrins, spectrins, and dystrophin-associated components, underscoring a tight coupling between ion channel function and the structural integrity of membrane microdomains. Notably, an additional module within this KCa3.1-TASK-1 intersection comprises proteins linked to Ca^2+^-dependent signalling and actin remodelling, including STIM1, STIM2, CAMLG and CAPZB. These features are consistent with coordinated Ca^2+^ sensing and membrane-associated cytoskeletal regulation within the shared KCa3.1-TASK-1 architectural framework. These modules are consistent with the KEGG enrichment profile observed for the shared KCa3.1-TASK-1 interactome (Figure 9).

The present architectural framework was further supported by pathway-level analyses of the KCa3.1-TASK-1 shared interactome. KEGG pathway enrichment analysis identified SNARE-mediated vesicular transport as the most significantly enriched category, followed by endocytosis and cytoskeleton-associated pathways, with additional representation of Hippo signalling (Figure 9, Supplementary Tables S5–S7). The mapping of shared interactors onto KEGG pathway diagrams was found to be consistent, with extensive coverage of endocytic and trafficking modules revealed. These modules included early endosomes, Endosomal Sorting Complexes Required for Transport (ESCRT) complexes, recycling compartments, and vesicle fusion machinery (Supplementary Figure S3A). Concurrently, Hippo signalling pathway mapping demonstrated the convergence of junctional scaffolds and signalling intermediates at adherens junctions, thereby substantiating a role for the shared interactome in the spatial integration of contact-dependent growth control pathways (Supplementary Figure S3B). Furthermore, pathway reconstruction of cytoskeletal organization modules revealed dense representation of spectrin-, dystrophin-, and actin-associated complexes linking membrane adhesion sites to intracellular force transmission systems (Supplementary Figure S3C).

Collectively, these analyses indicate that the functional overlap between KCa3.1 and TASK-1 is embedded within an integrated membrane-cytoskeleton-trafficking axis (Figure 9; Supplementary Figure S3A–C; Supplementary Tables S5–S7). This convergence is observed primarily at the level of membrane organization, trafficking, and scaffolding architecture. While several shared proteins have documented roles in signalling pathways, the present analysis does not imply that all detected associations correspond to direct functional interactions. Rather, the data support the existence of a common organizational framework that may facilitate context-dependent communication between signalling, trafficking, and cytoskeletal systems.

### 2.5. Kir2.1 ∩ TASK-1: Modular and Pathway-Level Organization

To further refine the functional organization, the Kir2.1-TASK-1 interactome was further analysed using STRING-based network clustering and enrichment analyses. Visualization of the intersecting PPI network revealed a densely connected architecture composed of multiple interlinked modules corresponding to vesicular trafficking, junctional scaffolding, cytoskeletal organization, and receptor-associated signalling (Figure 7C). Notably, the shared network is not dominated by a single signalling pathway. Instead, it is organized around proteins that coordinate membrane dynamics and the spatial distribution of signalling complexes, suggesting that convergence between Kir2.1 and TASK-1 occurs primarily at the level of cellular organization rather than through a common downstream signalling program.

Network partitioning revealed a significant endomembrane trafficking module, which was found to be enriched in COPII coat components (SEC23A/B, SEC24A/B, SEC31A), ER-Golgi tethering factors (SCFD1, TRIP11), and endosomal sorting machinery (HGS, STAM, GGA1-3, ZFYVE16). This module was found to be structurally connected to a junctional and adhesion-associated module, comprising JUP, DSG2, ALCAM, SCRIB, and integrin-linked receptors.

**Figure 10:**
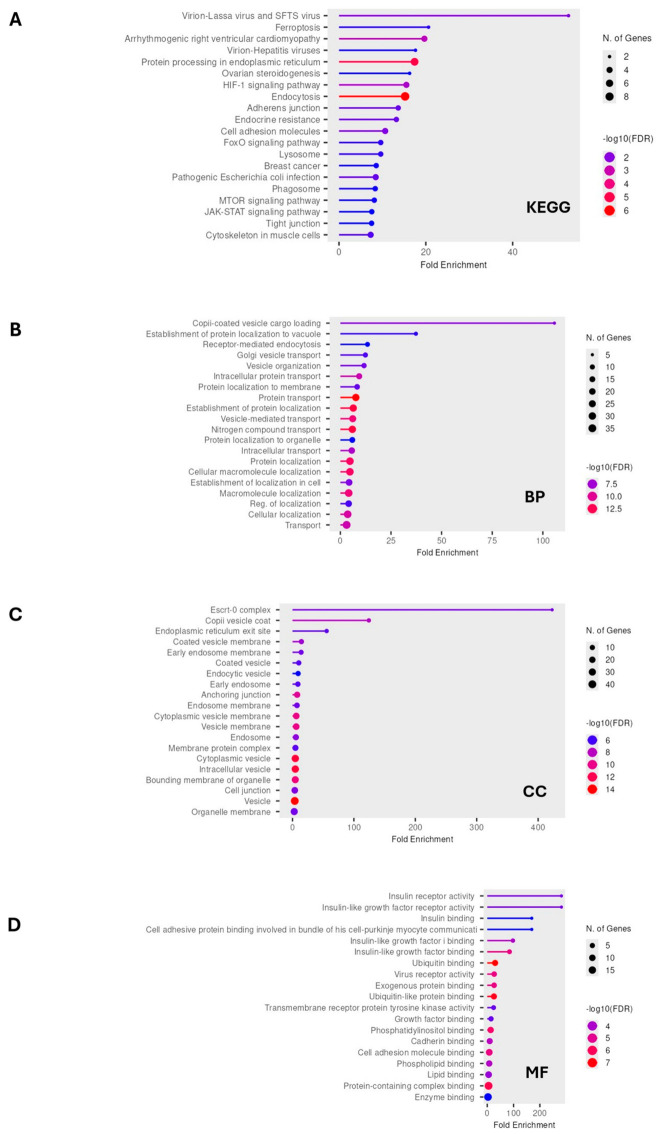
Gene Ontology and KEGG enrichment dot plots quantitatively define the architectural bias of the Kir2.1-TASK-1 shared interactome. (**A**) KEGG pathway enrichment analysis highlights vesicle-mediated transport, endocytosis, cytoskeleton-associated pathways, and receptor-linked signaling as dominant functional categories. (**B**) GO Biological Process enrichment emphasizes COPII-coated vesicle transport, receptor-mediated endocytosis, intracellular protein trafficking, and establishment of protein localization. (**C**) GO Cellular Component enrichment maps shared interactors to ESCRT complexes, COPII vesicles, endosomal membranes, anchoring junctions, and membrane protein complexes. (**D**) GO Molecular Function enrichment identifies growth factor receptor binding, cadherin binding, ubiquitin binding, and adaptor/scaffold activity as predominant features. Together, these analyses demonstrate that the Kir2.1-TASK-1 shared interactome is organized around scaffolding and signal integration rather than enzymatic function.

This finding is corroborated by functional enrichment analyses, which further support the presence of a modular architecture. KEGG pathway enrichment identified endocytosis and vesicle-mediated transport as the most significantly enriched functional categories, with additional representation of adherens junctions and receptor-associated signalling pathways, including endocrine resistance, breast cancer, and mTOR signalling (Figure 10A–D; Supplementary Tables S8–S15). Consistent with this interpretation, enrichment of receptor-associated signalling pathways should not be interpreted as evidence that Kir2.1 and TASK-1 directly regulate all these pathways. Rather, these enrichments reflect the presence of shared adaptor proteins, trafficking regulators, and membrane-associated scaffolds that are known to participate in multiple signalling contexts. Consequently, pathway enrichment is used here to infer architectural organization rather than direct mechanistic coupling.

These pathway-level trends were further supported by Gene Ontology and KEGG enrichment dot plot analyses, which quantitatively highlighted vesicle-mediated transport, endomembrane compartments, and receptor-associated binding functions as dominant features of the Kir2.1-TASK-1shared interactome (Figure 10A–D, Supplementary Figure S4A–I, Supplementary Tables S8–S15). Gene Ontology biological process enrichment is dominated by terms related to COPII-coated vesicle cargo loading, receptor-mediated endocytosis, vesicle organization, intracellular protein transport, and establishment of protein localization (Figure 10B; Supplementary Tables S10 and S11).

Gene Ontology Cellular Component enrichment analysis was performed using the STRING database. The input dataset consists of proteins shared between Kir2.1 and TASK-1 interactomes. Statistical significance was assessed using false discovery rate (FDR)-adjusted *p*-values. Only enriched terms with FDR < 0.05 are reported. Shared interactors localize to ESCRT complexes, vesicular membranes, and anchoring junctions, indicating integration of trafficking and adhesion-related structures.

As illustrated in Figure 10C and Supplementary Tables S12 and S13, cellular component enrichment consistently mapped shared interactors to ESCRT complexes, COPII vesicle coats, endosomal and vesicular membranes, anchoring junctions, and membrane protein complexes.

At the molecular function level, the most significantly enriched categories included insulin and IGF receptor activity, growth factor binding, cadherin and cell adhesion molecule binding, ubiquitin binding, and adaptor-related protein interactions. These findings indicate that the shared interactome is primarily organized around scaffolding and signal integration rather than intrinsic enzymatic activity (Figure 10D; Supplementary Tables S14 and S15). Taken together, the enrichment analyses consistently indicate that the Kir2.1–TASK-1 overlap is centered on proteins involved in membrane trafficking, cargo sorting, junctional stability, and receptor-associated scaffolding (Figure 10D; Supplementary Tables S14 and S15). These categories are compatible with a role in organizing signalling-competent membrane domains rather than directly mediating specific oncogenic pathways. Accordingly, the shared interactome appears to define a structural platform capable of integrating receptor signalling, membrane turnover, and cell–cell communication in a context-dependent manner.

Collectively, these analyses indicate that the Kir2.1-TASK-1 shared interactome defines a structurally integrated membrane-cytoskeleton-trafficking axis that supports spatial coordination of receptor signalling and junctional organization (Figure 7C; Figure 10A–D). Notably, the majority of shared proteins belong to trafficking, scaffolding, and adhesion-related systems rather than to channel-specific signalling pathways. Therefore, the observed convergence is best interpreted as architectural rather than strictly functional. While some shared nodes may participate directly in signalling events, the current data primarily support the existence of a common organizational framework that facilitates communication between membrane trafficking, adhesion complexes, and receptor-associated signalling modules.

### 2.6. Architectural Convergence and Divergence Between KCa3.1, Kir2.1, and TASK-1

Network reconstruction and clustering, based on STRING-derived interaction datasets (Figure 7D), highlight a shared interactome core enriched in proteins involved in vesicular trafficking, endomembrane organization, and junctional complexes (Figure 6).

Despite this convergence, the internal topology of the shared network is not functionally symmetric. Clustering analysis (Table 3, Supplementary Tables S16 and S17) identifies modules associated with ER-Golgi trafficking (SEC24A/B, SCFD1, TRIP11), cell polarity and adhesion (SCRIB, JUP, DSG2), and receptor-mediated signalling and endocytosis (NOTCH2, IGF2R, HGS, ZFYVE16). The emergence of these modules suggests that the most conserved elements across the three channel interactomes correspond to processes required for membrane protein positioning, trafficking, and spatial organization. Notably, canonical oncogenic pathways do not dominate the triple-overlap network. Instead, the shared architecture is enriched for proteins that coordinate where signalling occurs within the cell rather than proteins that directly execute signalling responses.

These clusters are observed across pairwise intersections; however, their size, connectivity, and peripheral composition differ among channel combinations. The triple intersection (Figure 7D) contains a reduced subset of shared nodes relative to pairwise overlaps. These observations describe the structural organization of shared interactomes without assigning functional hierarchy or channel-specific dominance. Taken together, the data suggest that convergence among 3 independent channels, namely KCa3.1, Kir2.1, and TASK-1, occurs primarily through common architectural principles, including membrane trafficking, junctional organization, and scaffold-mediated compartmentalization. At the same time, the reduction in network size observed in the triple intersection indicates that channel-specific interactors remain the dominant contributors to biological specialization. Thus, architectural convergence coexists with functional divergence, providing a framework for understanding how distinct K^+^ channels may participate in related cellular processes while retaining unique regulatory roles. The major organizational principles emerging from the pairwise and triple-overlap analyses are summarized in Figure 6.

### 2.7. Kv11.1 (hERG) Exhibits a Proteostasis-Centered ER-Biased Architecture

To extend the comparative interactome analysis to voltage-gated K^+^ channels with prominent trafficking liabilities, the interactome of Kv11.1 (*KCNH2*/hERG) was examined. Kv11.1 serves as a paradigmatic example of a proteostasis-sensitive ion channel, whose folding efficiency, ER retention, and ER-associated degradation (ERAD) represent dominant regulatory checkpoints governing channel abundance and function [12]. The Kv11.1 interactome was characterized using an affinity purification-mass spectrometry approach in HEK293 cells, with the inclusion of Kv11.1 serving as a comparative outlier to test the hypothesis that K^+^ channel interactomes necessarily converge on signalling-competent, cancer-relevant architectures (Table 1, Figure 5). This analysis identified 572 Kv11.1-associated proteins, providing a broad overview of the molecular environment associated with Kv11.1 [54]. While this dataset represents one of the largest experimentally derived Kv11.1 interactomes reported to date, its interpretation must consider the strong influence of channel biosynthesis, folding, and trafficking processes on the proteins recovered.

As reported in the original AP-MS study [12], the Kv11.1 interactome is enriched in proteins involved in ER proteostasis, including molecular chaperones, co-chaperones, and components of the ER-associated degradation (ERAD) pathway. Consistent with extensive prior work on Kv11.1 trafficking deficiency, these interactions highlight a regulatory architecture heavily influenced by folding surveillance, quality control, and ER exit dynamics. Many of these interactions are directly linked to the biogenesis and maturation of Kv11.1 itself, supporting their likely functional relevance rather than representing generic proximity associations. Compared with the junction-centered and signalling-integrated architectures observed for Kir2.1, KCa3.1, and TASK-1, the Kv11.1 interactome shows comparatively limited representation of adhesion complexes, receptor-associated signalling scaffolds and cytoskeletal signalling modules. This distinction suggests that not all K^+^ channels converge on common membrane-associated signalling architectures. Instead, interactome organization appears to be strongly influenced by channel-specific regulatory constraints.

Rather than functioning as a broadly integrated membrane-proximal signalling hub, Kv11.1 appears to operate within an ER-biased, proteostasis-weighted organizational framework in which channel abundance and localization are tightly regulated by quality control checkpoints (Table 1, Figure 5). Although Kv11.1 has been widely implicated in cancer cell proliferation and survival through its conductive properties [4,43,46,55], its interactome architecture suggests that its regulation is more strongly shaped by folding and trafficking constraints than by stable integration into membrane-proximal signalling platforms.

### 2.8. Kv1.3 Defines a Mitochondria-Centered Signalling Bottleneck

In contrast to Kv11.1, whose interactome is constrained to ER proteostasis components, the voltage-gated K^+^ channel Kv1.3 is associated with an interactome strongly enriched in mitochondrial proteins and mitochondrial-related regulatory processes. The Kv1.3 interactome was identified using BioID-based proximity labelling in intact HEK293 cells, yielding a high-confidence network comprising 527 proximal proteins [21]. Network analysis uncovered a dual architectural arrangement, consisting of a peripheral module associated with adhesion, membrane trafficking, and cytoskeletal dynamics, and a densely interconnected mitochondrial-centered subnetwork enriched in metabolic, redox, and transcriptional regulators [21].

Functional validation in melanoma and pancreatic cancer models demonstrated that perturbation of Kv1.3 expression or activity remodels mitochondrial metabolism, STAT3- and p53-dependent signalling pathways and tumor growth, thereby establishing a direct link between interactome architecture and oncogenic phenotypes [21]. These findings indicate that the functional relevance of Kv1.3 is encoded not only by its electrophysiological properties but also by its capacity to organize signalling networks. The complete Kv1.3 co-immunoprecipitation dataset originally generated by Capera et al. [22] and used here for comparative interactome analyses is provided in Supplementary Table S18.

**Figure 11:**
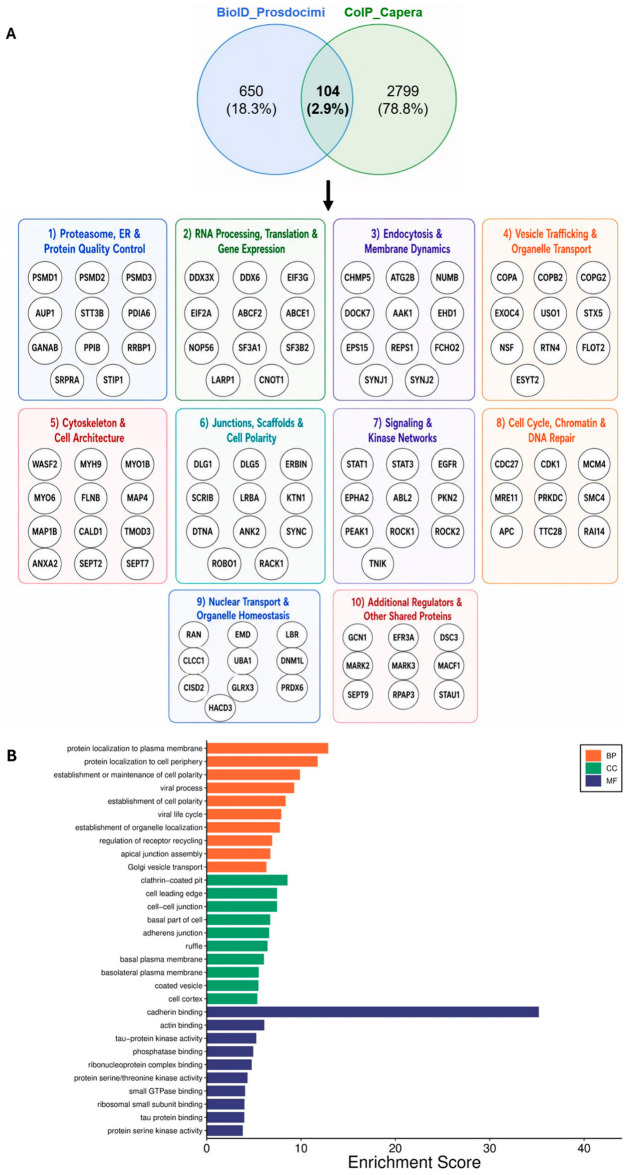

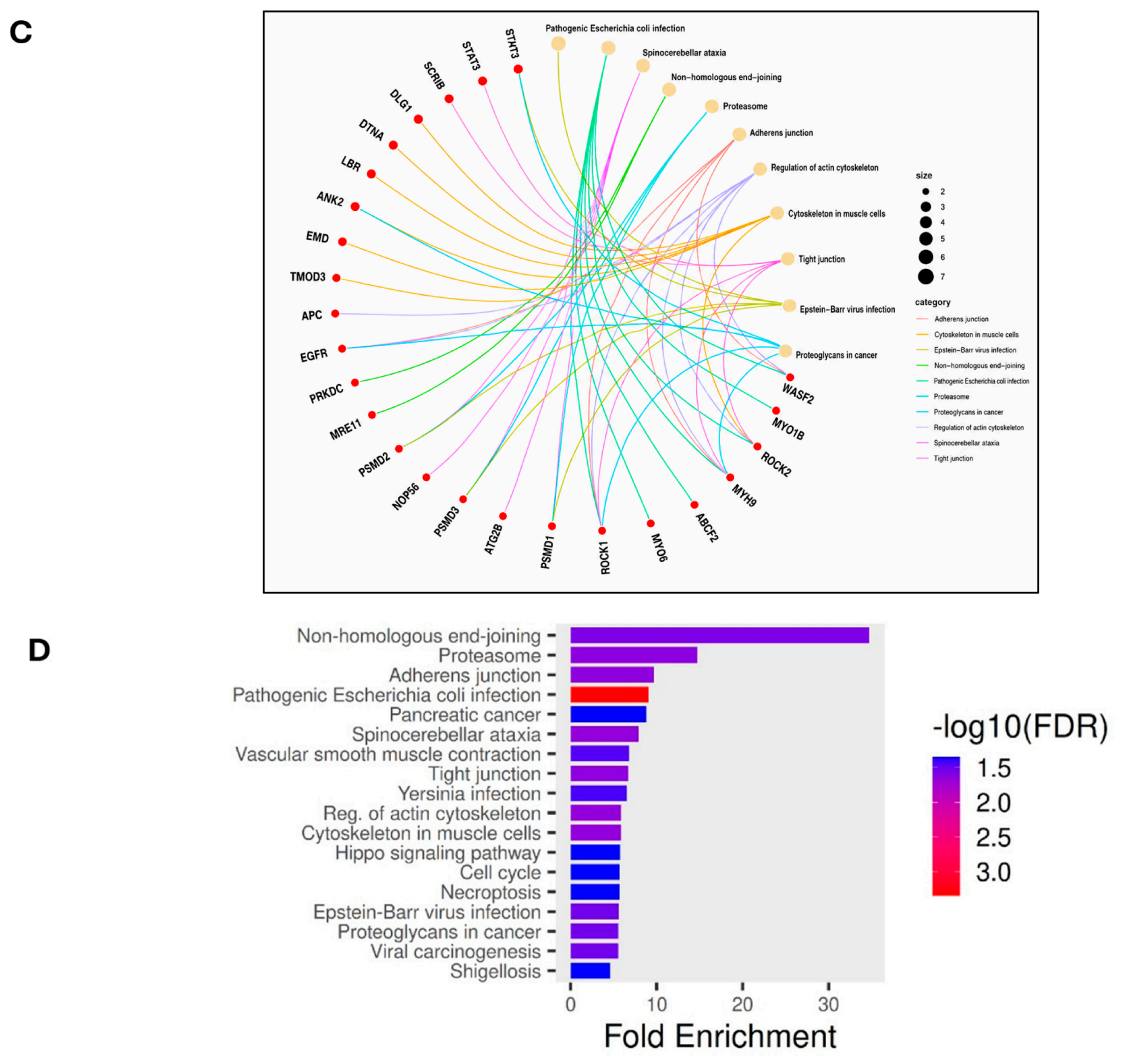
Functional characterization of the 104 proteins shared between the Kv1.3 BioID and Co-IP interactomes. (**A**) Venn diagram showing the overlap between proteins identified in the Kv1.3 BioID dataset reported by Prosdocimi et al. and the Kv1.3 co-immunoprecipitation (Co-IP) dataset generated by Capera et al. A total of 104 proteins were common to both datasets and were classified into ten functional categories: (1) Proteasome, ER and Protein Quality Control, (2) RNA Processing, Translation and Gene Expression, (3) Endocytosis and Membrane Dynamics, (4) Vesicle Trafficking and Organelle Transport, (5) Cytoskeleton and Cell Architecture, (6) Junctions, Scaffolds and Cell Polarity, (7) Signalling and Kinase Networks, (8) Cell Cycle, Chromatin and DNA Repair, (9) Nuclear Transport and Organelle Homeostasis, and (10) Additional Regulators and Other Shared Proteins. Functional categories were assigned based on Gene Ontology (GO) annotations, literature-supported functional classification, and manual biological curation of the shared protein set. Protein names associated with each category are shown within the corresponding panels. (**B**) Gene Ontology (GO) enrichment analysis of the 104 common proteins, categorized into Biological Process (BP), Cellular Component (CC), and Molecular Function (MF). Bars represent significantly enriched GO terms ranked according to their enrichment score. The most enriched terms were associated with protein localization to the plasma membrane, establishment of cell polarity, vesicle-mediated transport, cell junction organization, basolateral membrane components, and cadherin- and actin-binding functions. (**C**) Cnetplot showing the relationships between proteins and significantly enriched KEGG pathways. Red nodes represent proteins, whereas beige nodes represent enriched pathways. Pathway node size is proportional to the number of mapped proteins, and colored edges indicate protein–pathway associations. The network highlights the coordinated involvement of proteins in pathways related to cell-junction organization, regulation of the actin cytoskeleton, proteasome function, DNA repair through non-homologous end-joining (NHEJ), and cell-cycle regulation. Several proteins, including ROCK1, ROCK2, MYH9, WASF2, EGFR, DLG1, and SCRIB, were mapped to multiple pathways, suggesting potential roles as functional links between intracellular signaling, cell-junction organization, and cytoskeletal dynamics. (**D**) KEGG pathway enrichment analysis of the 104 common proteins. Bar length indicates fold enrichment, while the color scale represents statistical significance expressed as −log10(FDR). The non-homologous end-joining pathway showed the highest enrichment, followed by the proteasome and adherens junction pathways. Additional significantly enriched pathways included tight junctions, regulation of the actin cytoskeleton, cytoskeletal organization, Hippo signaling, and cell-cycle-related processes, indicating a prominent role of the common proteins in DNA repair, protein degradation, cellular architecture, signal transduction, and cell-cycle control. Data processing, annotation handling, and visualization for panel A were performed using Python v3.13.5 with pandas v2.2.3 and matplotlib v3.10.8. GO enrichment analysis and visualization for panel B were performed in R v4.6.0 using ggplot2 v4.0.3. Functional enrichment analysis and category–gene network visualization for panel C were performed in R v4.6.0 using clusterProfiler v4.20.0, enrichplot v1.32.0 (cnetplot function), and ggplot2 v4.0.3. KEGG pathway enrichment analysis and visualization for panel D were generated using ShinyGO v0.85.1 [56].

**Figure 12:**
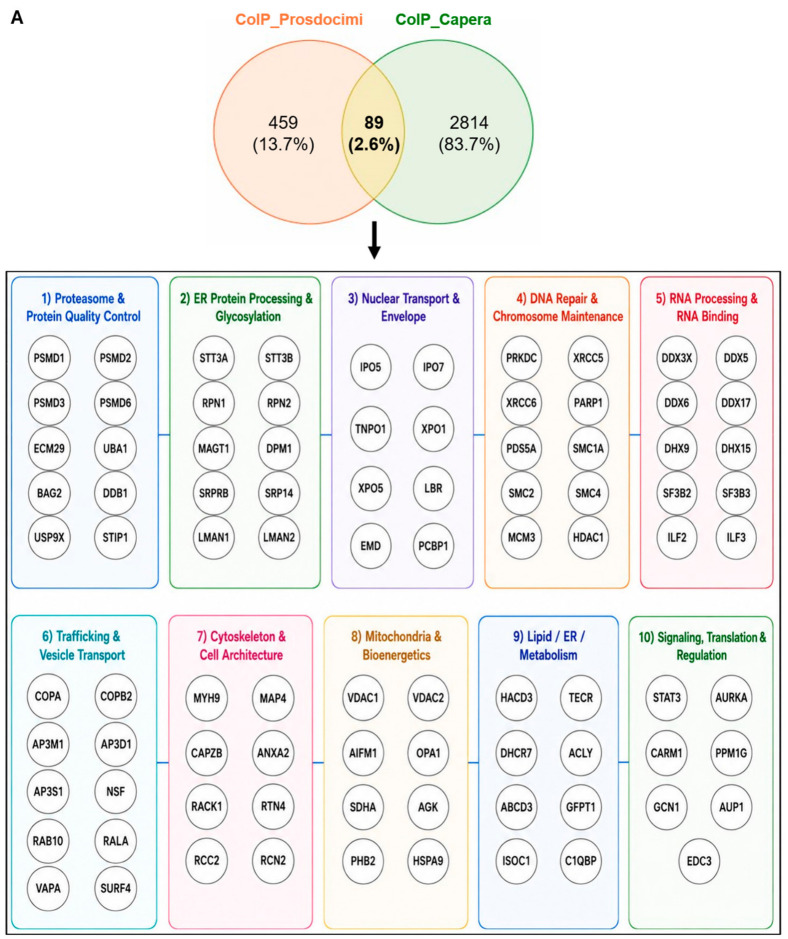

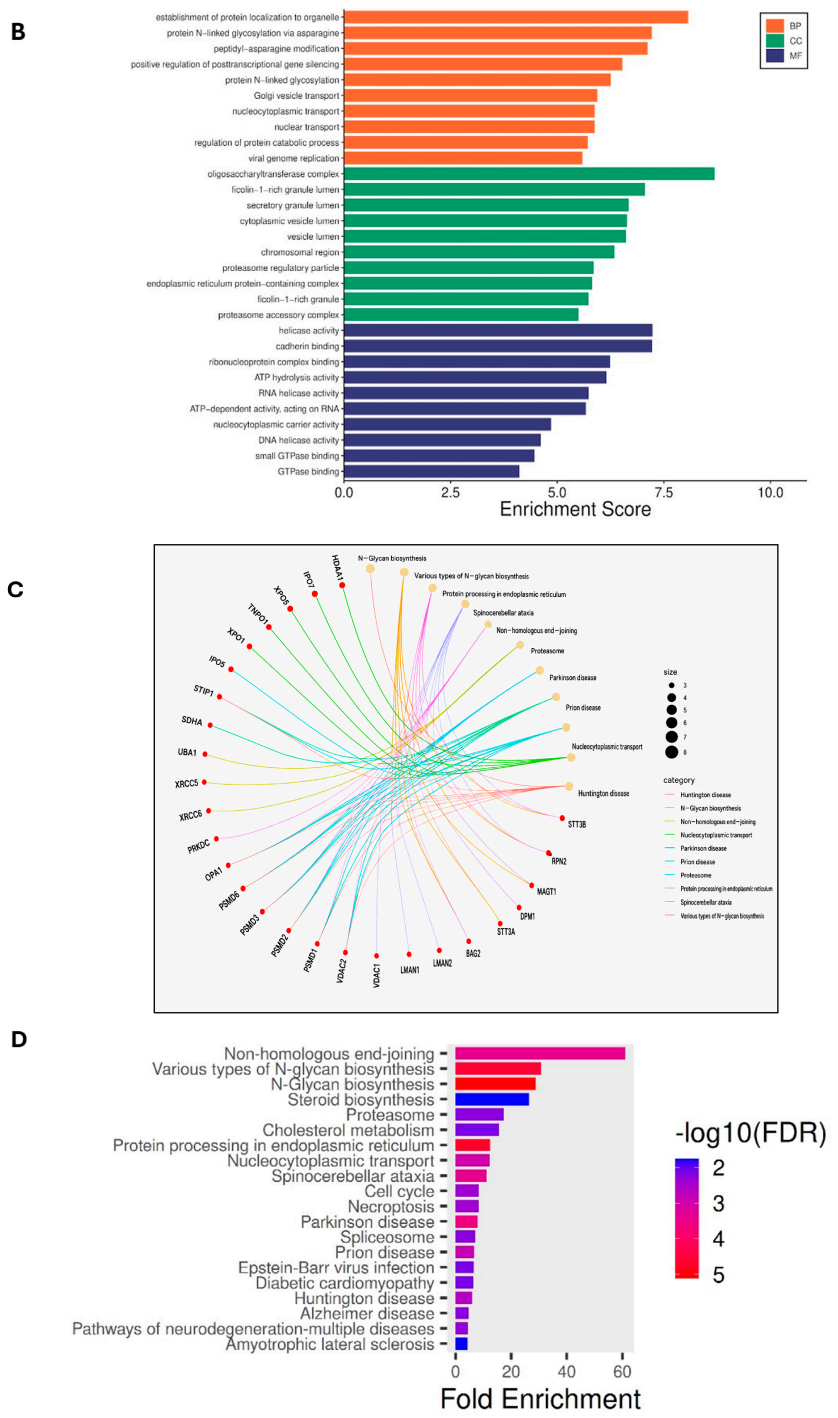
Functional characterization of the 89 proteins shared between Co-IP datasets. (**A**) Venn diagram showing the overlap between proteins identified in the Prosdocimi and Capera co-immunoprecipitation (Co-IP) datasets. A total of 89 proteins were common to both datasets and were classified into ten functional categories: (1) Proteasome and Protein Quality Control, (2) ER Protein Processing and Glycosylation, (3) Nuclear Transport and Envelope, (4) DNA Repair and Chromosome Maintenance, (5) RNA Processing and RNA Binding, (6) Trafficking and Vesicle Transport, (7) Cytoskeleton and Cell Architecture, (8) Mitochondria and Bioenergetics, (9) Lipid/ER Metabolism, and (10) Signaling, Translation and Regulation. Functional categories were assigned based on KEGG pathway enrichment analysis, Gene Ontology (GO) annotations, and curated biological interpretation of the shared protein set. Protein names associated with each category are shown within the corresponding panels. The connecting lines indicate shared membership within the functional classification scheme and do not represent direct protein–protein interactions. (**B**) Gene Ontology (GO) enrichment analysis of the 89 common proteins, categorized into Biological Process (BP), Cellular Component (CC), and Molecular Function (MF). Bars represent significantly enriched GO terms ranked according to their enrichment score. The most enriched biological processes were associated with protein localization to organelles, N-linked glycosylation, post-translational protein modification, Golgi vesicle transport, nucleocytoplasmic transport, and regulation of protein catabolism. Cellular component enrichment highlighted oligosaccharyltransferase complexes, secretory and cytoplasmic vesicle lumens, proteasome-associated complexes, and endoplasmic reticulum protein-containing compartments, whereas molecular function analysis revealed enrichment of helicase activity, cadherin binding, ATP hydrolysis, RNA helicase activity, nucleocytoplasmic carrier activity, and small GTPase binding. (**C**) Cnetplot showing the relationships between proteins and significantly enriched KEGG pathways. Red nodes represent proteins, whereas beige nodes represent enriched pathways. Pathway node size is proportional to the number of mapped proteins, and colored edges indicate protein–pathway associations. The network highlights the involvement of the shared proteins in N-glycan biosynthesis, protein processing in the endoplasmic reticulum, nucleocytoplasmic transport, proteasome function, and DNA repair through non-homologous end-joining (NHEJ). Several proteins were mapped to multiple pathways, including STT3A, STT3B, RPN1, RPN2, and MAGT1, which connected N-glycan biosynthesis with endoplasmic-reticulum protein-processing pathways, while PSMD1, PSMD2, PSMD3, and PSMD6 contributed to proteasome-related processes. The network provides a detailed overview of the proteins underlying pathway enrichment and identifies proteins shared across functionally related cellular processes. (**D**) KEGG pathway enrichment analysis of the 89 common proteins. Bar length indicates fold enrichment, while the color scale represents statistical significance expressed as −log10(FDR). Non-homologous end-joining displayed the highest fold enrichment and was represented by XRCC5, XRCC6, and PRKDC, indicating enrichment of DNA double-strand break repair mechanisms. Strong enrichment was also observed for pathways related to N-glycan biosynthesis, supported by STT3A, STT3B, RPN1, RPN2, MAGT1, DPM1, LMAN1, LMAN2, and BAG2, highlighting processes involved in protein glycosylation, folding, maturation, and trafficking within the endoplasmic reticulum. Additional enriched pathways included steroid biosynthesis, cholesterol metabolism, proteasome function, protein processing in the endoplasmic reticulum, and nucleocytoplasmic transport, the latter involving IPO5, IPO7, TNPO1, XPO1, and XPO5. Overall, the enrichment analysis indicates a prominent involvement of the conserved Kv1.3 interactome in protein maturation, intracellular trafficking, protein quality control, and DNA repair. Data processing, annotation handling, and visualization for panel A were performed using Python v3.13.5 with pandas v2.2.3 and matplotlib v3.10.8. GO enrichment analysis and visualization for panel B were performed in R v4.6.0 using ggplot2 v4.0.3. Functional enrichment analysis and category–gene network visualization for panel C were performed in R v4.6.0 using clusterProfiler v4.20.0, enrichplot v1.32.0 (cnetplot function), and ggplot2 v4.0.3. KEGG pathway enrichment analysis and visualization for panel D were generated using ShinyGO v0.85.1 [56].

Intersection of the BioID interactome reported by Prosdocimi et al. [21] with the filtered co-immunoprecipitation dataset generated in the same study yielded a core set of shared proteins, defining a technique-independent Kv1.3 interactome that remains strongly enriched in mitochondrial components involved in proteostasis, RNA metabolism, and transcriptionally relevant signalling nodes [21] (Figure 11A, Supplementary Table S20). Functional enrichment analysis revealed significant overrepresentation of proteins involved in cell-junction organization, cytoskeletal regulation, vesicle trafficking, proteasome function, and DNA repair pathways (Figure 11B–D). Extension of this comparison to an independent co-IP dataset we generated (see also [22]), revealed substantial convergence despite distinct experimental strategies, with 89 proteins shared between co-IP datasets and 104 BioID-derived interactors independently recovered (Figure 11 and Figure 12, Supplementary Tables S19 and S20). Notably, this conserved core includes key mitochondrial and signalling components such as VDAC1/2, OPA1, PHB2, AIFM1, HSPA9, PRKDC, and STAT3, indicating that the bottleneck architecture inferred from proximity labelling is preserved when only stable biochemical interactions identified by Co-IP are considered. The recovery of these proteins across orthogonal experimental approaches increases confidence that the mitochondrial module does not arise solely from method-specific proximity effects. Nevertheless, the persistence of an interaction across datasets should not be interpreted as proof of direct functional regulation, and further experimental validation will be required to establish the mechanistic contribution of individual interactors.

The robustness of the proposed Kv1.3-centered mitochondrial bottleneck is supported by this convergence across orthogonal experimental strategies (Figure 11 and Figure 12, Supplementary Tables S19 and S20). At the same time, the intersecting datasets reveal that the conserved Kv1.3 interactome extends beyond mitochondrial metabolism to include vesicle trafficking and endomembrane organization, cytoskeletal dynamics, RNA metabolism and ubiquitin-proteasome system components. This breadth indicates that the mitochondrial module is embedded within a broader cellular infrastructure rather than representing an isolated signalling island. Accordingly, the proposed mitochondrial bottleneck should be interpreted as a dominant organizational tendency within the Kv1.3 interactome rather than as an exclusive functional compartment. Collectively, the overlap analyses summarized in Figure 11 and Figure 12 support the existence of a conserved Kv1.3-centered mitochondrial signaling architecture that is reproducibly recovered across orthogonal experimental approaches.

The comparison of datasets from independent studies [21,22] and a new co-IP analysis revealed 89 common proteins (Figure 12A, Supplementary Table S19). Functional enrichment analysis of these shared proteins highlighted pathways involved in protein maturation, N-glycan biosynthesis, endoplasmic-reticulum protein processing, nucleocytoplasmic transport, proteasome function, and DNA repair (Figure 12B–D). Many of these proteins are core mitochondrial, metabolic, or stress-response factors (VDAC1/2, SDHA, ACLY, HSPA9, PRKDC, and PARP1), supporting the existence of a conserved Kv1.3-associated mitochondrial environment across distinct cellular contexts (Figure 12A, Supplementary Table S19). At the same time, the presence of abundant housekeeping proteins and multifunctional chaperones within these intersections highlights an intrinsic limitation of in-depth interactomic approaches, in which highly connected and abundant proteins may contribute to apparent convergence without necessarily encoding channel-specific regulatory functions (Figure 12B–D).

### 2.9. Critical Considerations in the Cross-Study Comparison of Kv1.3 Interactomes

**Figure 13:**
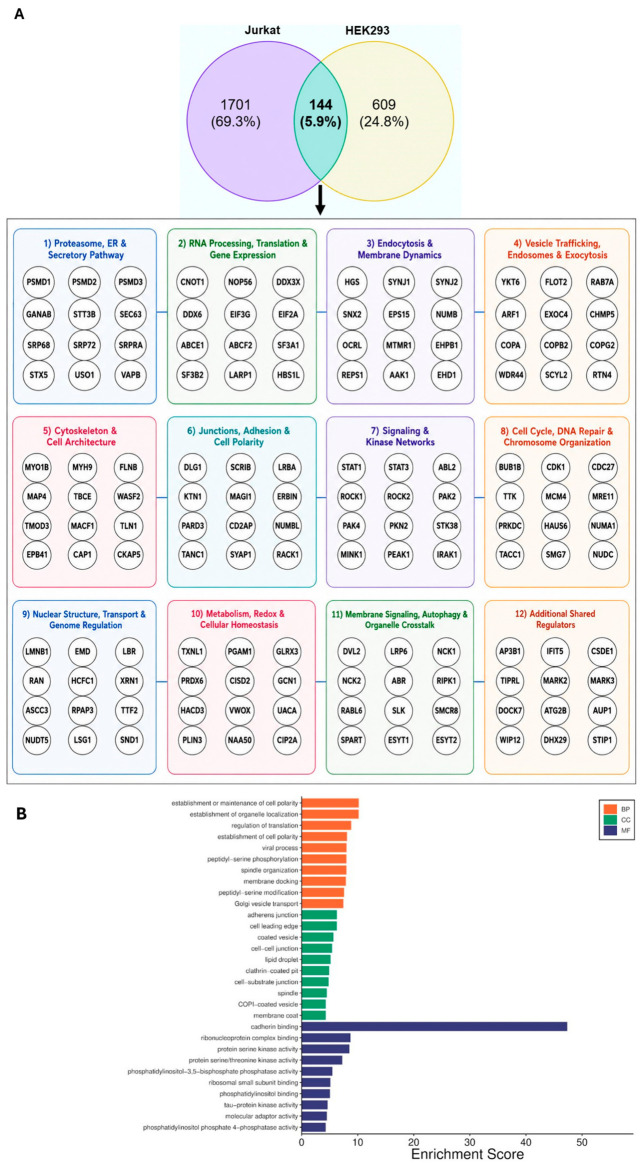

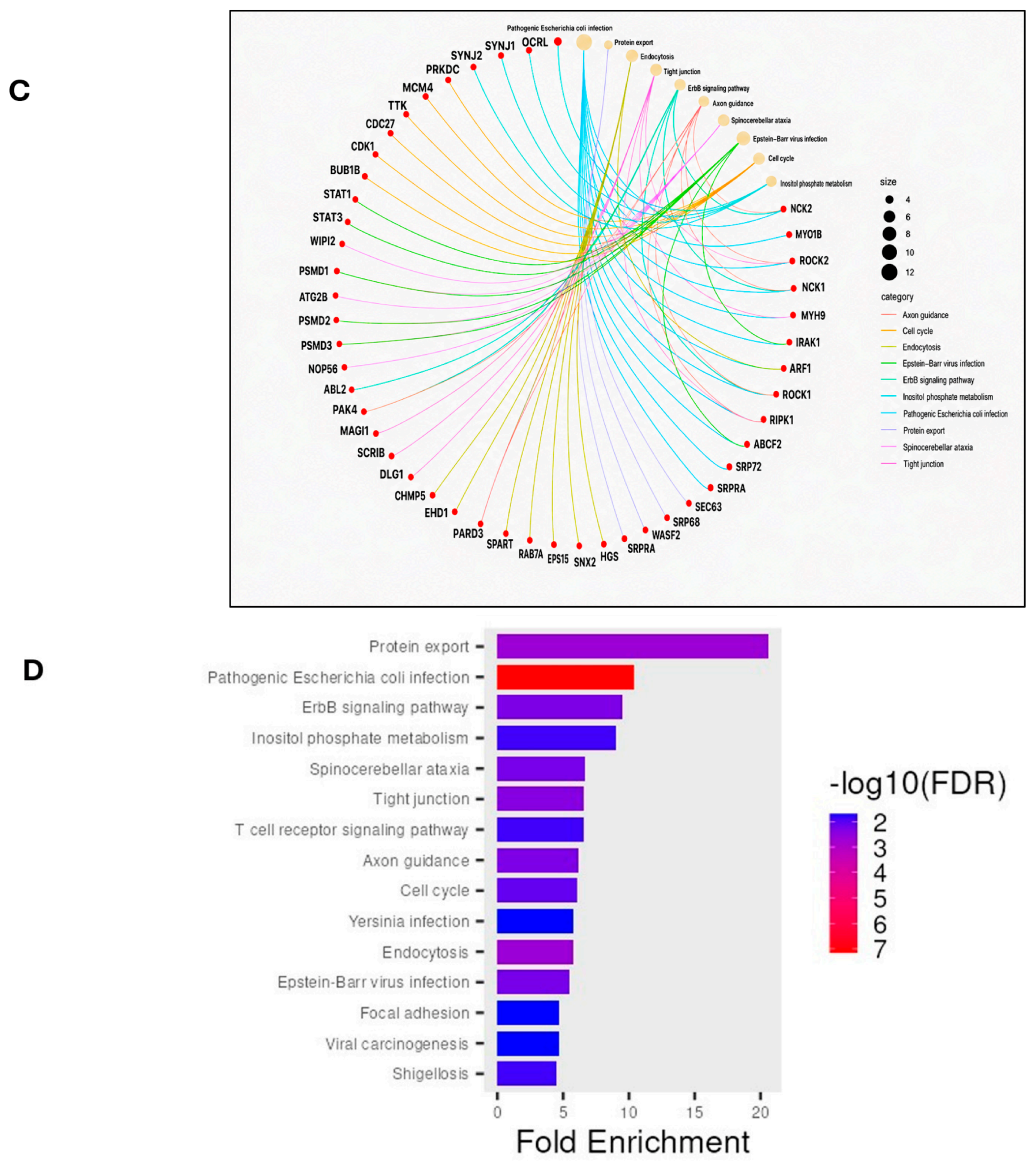
Functional characterization of proteins shared between Jurkat and HEK293 datasets. (**A**) Venn diagram showing the overlap between proteins identified in Jurkat and HEK293 cells. A total of 144 proteins were common to both datasets and were grouped into twelve functional categories: (1) Proteasome, ER and Secretory Pathway, (2) RNA Processing, Translation and Gene Expression, (3) Endocytosis and Membrane Dynamics, (4) Vesicle Trafficking, Endosomes and Exocytosis, (5) Cytoskeleton and Cell Architecture, (6) Junctions, Adhesion and Cell Polarity, (7) Signaling and Kinase Networks, (8) Cell Cycle, DNA Repair and Chromosome Organization, (9) Nuclear Structure, Transport and Genome Regulation, (10) Metabolism, Redox and Cellular Homeostasis, (11) Membrane Signaling, Autophagy and Organelle Crosstalk, and (12) Additional Shared Regulators. Functional categories were defined based on KEGG pathway enrichment analysis, Gene Ontology (GO) annotations, and curated biological interpretation of the shared protein set. Protein names assigned to each category are displayed within the corresponding panels. The connecting lines indicate shared membership within the functional classification framework and do not represent direct protein–protein interactions. (**B**) Gene Ontology (GO) enrichment analysis of the common proteins, categorized into Biological Process (BP), Cellular Component (CC), and Molecular Function (MF). Bars represent significantly enriched GO terms ranked according to their enrichment score. The most enriched biological processes were associated with the establishment and maintenance of cell polarity, organelle localization, regulation of translation, protein phosphorylation, spindle organization, membrane docking, and Golgi vesicle transport. Cellular component enrichment highlighted adherens junctions, cell–cell junctions, coated vesicles, clathrin-coated pits, cell–substrate junctions, and COPII-coated vesicles, whereas molecular function analysis revealed a strong enrichment for cadherin binding, kinase-related activities, phosphatidylinositol binding, molecular adaptor activity, and small ribosomal subunit binding. (**C**) Cnetplot showing the relationships between proteins and significantly enriched KEGG pathways. Red nodes represent proteins, whereas beige nodes represent enriched pathways. Pathway node size is proportional to the number of mapped proteins, and colored edges indicate protein–pathway associations. The network highlights the involvement of the shared proteins in pathways related to protein trafficking, endocytosis, cell-junction organization, cytoskeletal regulation, intracellular signaling, and cell-cycle control. Several proteins were mapped to multiple pathways, including NCK1, NCK2, PAK2, ROCK1, and ROCK2, which connected signaling pathways with cytoskeletal and junction-associated processes. The cnetplot provides a detailed overview of the proteins underlying the enrichment patterns and identifies proteins that may function as integrators of signaling events and structural cellular organization. (**D**) KEGG pathway enrichment analysis of the shared proteins. Bar length indicates fold enrichment, while the color scale represents statistical significance expressed as −log10(FDR). Protein export displayed the highest fold enrichment, indicating a marked overrepresentation of proteins involved in intracellular protein transport. Additional significantly enriched pathways included ErbB signaling, inositol phosphate metabolism, tight junctions, endocytosis, focal adhesion, axon guidance, and cell-cycle-related processes, highlighting the contribution of the shared proteins to membrane trafficking, cellular architecture, cytoskeletal organization, and signal transduction. Overall, the enrichment analysis suggests a prominent role of the conserved Kv1.3-associated proteins in coordinating protein trafficking, intracellular signaling, cytoskeletal dynamics, and cell-junction organization. Data processing, annotation handling, and visualization for panel A were performed using Python v3.13.5 with pandas v2.2.3 and matplotlib v3.10.8. GO enrichment analysis and visualization for panel B were performed in R v4.6.0 using ggplot2 v4.0.3. Functional enrichment analysis and category–gene network visualization for panel C were performed in R v4.6.0 using clusterProfiler v4.20.0, enrichplot v1.32.0 (cnetplot function), and ggplot2 v4.0.3. KEGG pathway enrichment analysis and visualization for panel D were generated using ShinyGO v0.85.1 [56].

While the identification of shared Kv1.3-interacting proteins across studies is informative, the biological significance of interactome convergence can only be evaluated in the context of methodological and cellular differences. Therefore, interpretation of the overlap between datasets requires careful consideration of experimental design, cell type, and proximity-labelling strategy. Several methodological differences must be acknowledged when comparing the TurboID-based Jurkat dataset reported by Kour and colleagues [57,58] with our BioID dataset reported by Prosdocimi et al. [21] (Figure 13, Supplementary Table S21). First, the two interactomes were generated in distinct cellular systems. The Jurkat dataset was obtained in human T lymphocytes [57,58], whereas the Prosdocimi study was conducted in HEK293 cells [19]. These cell lines differ substantially in lineage, differentiation state, and basal proteome composition. Therefore, differences in interactome composition may reflect cell-type–specific protein expression rather than differential Kv1.3 binding per se (Figure 13, Supplementary Table S21). Functional enrichment analysis of the proteins shared between the BioID and TurboID datasets revealed significant enrichment of pathways involved in protein trafficking, endocytosis, cell-junction organization, cytoskeletal regulation, intracellular signaling, and cell-cycle control (Figure 13B–D). Several proteins, including NCK1, NCK2, PAK2, ROCK1, and ROCK2, were associated with multiple enriched pathways, suggesting a potential role in coordinating signaling events with cytoskeletal remodeling and cellular organization. Second, the proximity labeling enzymes differ between studies. The Jurkat dataset was generated using TurboID, whereas Prosdocimi et al. employed BioID. These techniques rely on different BirA* enzymes which have distinct labelling kinetics and efficiencies, which can affect the extent and sensitivity of proximity labelling. Consequently, the overall size of the detected interactome differs markedly between the two studies (e.g., 527 total hits reported in Prosdocimi et al. versus a substantially larger AP-enriched set in Jurkat cells) (Figure 13). This difference alone limits direct quantitative comparison. Third, statistical filtering criteria are not identical. Prosdocimi et al. defined hits using a fold change > 2 (log_2_FC > 1) and FDR < 0.05, whereas the Jurkat dataset applied more stringent thresholds (log_2_FC > 2 and *p* < 0.05) but did not apply Jurkat cells expressing only BirA* to subtract proteins from the interactome list that are endogenously biotinylated independently of Kv1.3. Thus, divergent statistical cutoffs and the employed controls directly influence the number of proteins retained as significant interactors and therefore impact overlap analyses.

These comparative analyses indicate that while the Kv1.3 interactome reproducibly converges on a mitochondria-centered core across multiple methodologies, the biological interpretation of this bottleneck must account for the composite nature of the recovered networks (Figure 13, Supplementary Table S21). Rather than defining a single, invariant signalling architecture, Kv1.3 appears to participate in a conserved mitochondrial milieu whose functional output is shaped by cellular context, expression level, and metabolic state, extending its role beyond ion conduction to the coordination of mitochondrial state and nuclear gene expression programs with direct relevance to cancer signalling.

## 3. Materials and Methods

### 3.1. Cell Culture and Transient Transfection

HEK293 cells were cultured in Dulbecco’s Modified Eagle Medium (DMEM) supplemented with 10% fetal bovine serum (FBS) and 100 U/mL penicillin–streptomycin at 37 °C in a humidified atmosphere containing 5% CO_2_. Cells were transiently transfected at approximately 80% confluence, and experiments were performed 24 h after transfection.

### 3.2. High-Confidence Kv1.3 Interactome Identified by Co-IP and LC-MS/MS

The Kv1.3 interactome was defined by co-immunoprecipitation of Kv1.3-GFP from HEK293 cells followed by LC-MS/MS, as previously described [22]. Proteins identified with unique peptides and high-confidence detection criteria are listed in Supplementary Table S18.

### 3.3. Systematic Literature Search and Dataset Collection

A systematic literature survey was performed to identify experimentally supported potassium (K^+^) channel interactome studies. PubMed database was queried using combinations of keywords including potassium channel, interactome, protein–protein interaction, proteomic, proximity labeling, BioID, TurboID, APEX, and affinity purification. A meta-analytic strategy was applied to ensure comprehensive coverage of the available literature. Initial records were screened based on title and abstract, followed by full-text evaluation. Studies were retained if they reported experimentally derived interactome or proximity-based proteomic datasets centered on K^+^ channels. Only studies providing explicit lists of interacting or proximal proteins were considered for downstream analysis. Reviews, purely computational predictions, and studies lacking protein-level interaction data were excluded.

### 3.4. Inclusion Criteria and Interactome Dataset Curation

Interactome datasets were curated based on strict inclusion criteria. Only studies employing experimentally supported approaches—such as BioID, TurboID, APEX-based proximity labeling, co-immunoprecipitation, or affinity purification—mass spectrometry—were retained. Datasets derived from heterogeneous cellular systems and experimental platforms were intentionally included to capture the diversity of channel-associated interaction environments. Importantly, interactomes were not restricted to cancer-derived systems at this stage, allowing unbiased identification of conserved and divergent organizational principles.

Protein identifiers across all datasets were standardized to official gene symbols prior to integration. Matching, aggregation, and identification of shared proteins across studies were performed using custom scripts developed in Python (version 3.13.5). These scripts relied on standard data-processing libraries, including pandas and NumPy, to compare protein lists, remove redundancies, and extract pairwise and higher-order intersections.

To improve curation accuracy and reduce errors arising from heterogeneous nomenclature and annotation, the results of the initial matching step were further evaluated using algorithmic, computational validation procedures. This step served as an auxiliary validation layer and leveraged patterns of functional and structural similarity to enhance the internal consistency of the integrated interactome datasets.

### 3.5. Pan-Cancer Transcriptomic and Genomic Analyses

Gene-level differential expression between tumor and adjacent normal tissues was analyzed using the Gene_DE module of the TIMER3 web server across all available TCGA cancer types. This module performs differential expression analysis on RNA-seq raw counts using the edgeR statistical framework. For each gene of interest, expression distributions in tumor and normal samples were visualized using box plots.

Statistical significance of differential expression was evaluated using edgeR-derived *p*-values. Significance levels were defined as *p* < 0.05, *p* < 0.01, and *p* < 0.001, corresponding to increasing levels of confidence. Where applicable, *p*-values were adjusted for multiple testing using the Benjamini–Hochberg false discovery rate (FDR) correction, and genes with FDR < 0.05 were considered statistically significant. Genes were classified as upregulated or downregulated in tumors relative to normal tissues based on the direction of the estimated log2 fold change.

In addition to gene-level expression analyses, somatic mutation profiles of selected K^+^ channel genes were examined using the Gene_Mutation module of the TIMER3 web server (https://timer.cistrome.org accessed on 15 April 2026). Mutation frequencies across TCGA cancer types were summarized, and mutation-associated expression differences were visualized using a targeted heatmap representation, as shown in Figure 3. This analysis was performed to assess the distribution and relative prevalence of K^+^ channel gene mutations across cancer types, rather than to conduct comprehensive multi-gene mutation signature analyses.

All TIMER analyses were conducted using the most recent TIMER3 release, as described in Cui et al., 2025 [59].

### 3.6. Interactome Data Processing and Functional Classification

Protein lists derived from curated interactome studies were processed exclusively using Python (version 3.12). All steps related to identifier standardization, list comparison, functional categorization, and group assignment were performed programmatically using custom Python scripts.

Protein identifiers originating from heterogeneous sources (e.g., UniProt accessions, gene symbols, or study-specific nomenclature) were first harmonized to official gene symbols. Identifier normalization and validation were implemented using dictionary-based mapping and string-matching procedures to eliminate redundancy and inconsistent naming. Duplicate entries were removed to ensure that each protein was represented uniquely across datasets.

Core data processing steps—including aggregation of interactors across studies, pairwise and multi-set comparison, and assignment of proteins to functional categories—were implemented using standard Python data-processing libraries (pandas, NumPy). Specifically, data processing and interactome curation were performed using Python 3.13.5 with pandas 2.2.3, NumPy 2.5.0, and matplotlib 3.10.8. Functional enrichment analyses were performed in R 4.6.0 using clusterProfiler 4.20.0, enrichplot 1.32.0, and ggplot2 4.0.3. KEGG pathway enrichment visualization was additionally performed using ShinyGO v0.85.1 (Ge SX, Jung D, and Yao R, Bioinformatics 36:2628–2629, 2020). The corresponding software information has been added to the Materials and Methods section. Operations such as list merging, set intersection, and uniqueness filtering were performed using built-in data structures (e.g., set, merge, drop_duplicates) to ensure reproducibility and transparency.

Based on literature-supported functional annotations, proteins were classified into four predefined functional categories: proteostasis, cytoskeleton, vesicle trafficking, and signalling. Functional group assignment was performed algorithmically in Python by matching curated protein lists against category-specific reference sets, followed by manual validation to ensure biological consistency. This categorization enabled systematic comparison of interactome composition across channels and datasets.

Visualization of functional intersections across these four categories was performed separately using R (version 4.4.1).

### 3.7. Network Reconstruction, Intersection, and Enrichment Analyses

Protein–protein interaction (PPI) networks were reconstructed using the STRING database (version 12.0). Curated protein lists corresponding to channel-specific and shared interactomes were uploaded to STRING, and interaction networks were generated based on STRING’s integrated evidence framework (including experimentally supported interactions, curated databases, co-expression, and text mining). To reduce low-confidence associations, a medium-to-high confidence interaction score threshold (≥0.4) was applied. STRING was used exclusively for PPI network reconstruction and visualization of interaction structure, ensuring that the reported network connectivity reflects established interaction evidence rather than externally inferred edges.

Intersection analyses were performed to identify shared and channel-specific interactors across single-channel, pairwise, and higher-order channel combinations. Overlap sets were first defined at the protein list level, and intersection-specific PPI networks were then generated in STRING using only the proteins present in each overlap set. This workflow provides a consistent and evidence-based representation of shared network architecture across channel combinations, while maintaining a clear separation between list-level overlap definition and network-level visualization.

Functional enrichment analyses were performed in R (version 4.4.1) using Gene Ontology (Biological Process, Cellular Component, Molecular Function) and KEGG pathway annotations. Statistical significance of enrichment was evaluated using hypergeometric testing, and *p*-values were adjusted for multiple testing using the Benjamini–Hochberg false discovery rate (FDR) correction. Enriched terms were considered statistically significant at FDR < 0.05, unless otherwise specified.

KEGG pathway mapping and visualization were carried out in R using Pathview, enabling graphical projection of shared interactors onto canonical KEGG pathway diagrams. Enrichment dot plots were generated in R, with dot size representing the number of genes contributing to each term/pathway and color encoding −log10(FDR), as shown in the corresponding Supplementary Figures.

## 4. Conclusions

The present comparative analysis indicates that K^+^ channel function in cancer is not primarily defined by conserved molecular interactors, but rather by recurrent higher-order organizational architectures integrating trafficking, adhesion, metabolic, lipid-signalling, and proteostatic modules. Although the individual interactomes display limited direct protein overlap, they repeatedly converge on spatially organized signalling frameworks that coordinate membrane dynamics, receptor-associated signalling, compartmentalized metabolism, and cellular stress adaptation. Consistent with this framework, our analyses first examined whether the cancer relevance of selected K^+^ channels could be interpreted from gene-level features alone. Across tumour types, transcript abundance, co-expression patterns, and somatic mutation frequencies of the analysed channels displayed substantial heterogeneity and limited coordination, supporting the view that gene-level descriptors are insufficient to rationalize functional convergence across tumour contexts.

To explore an alternative organizational layer, we performed a cross-study comparison of experimentally supported proximity-labelling and affinity-purification interactomes and reported a new co-IP analysis for Kv1.3 [22]. Although the underlying datasets were generated in different cellular systems and using distinct labelling chemistries and analytical thresholds, network reconstruction combined with harmonized pathway and topology analyses indicates that K^+^ channel-associated environments can be described by a restricted set of recurrent architectural patterns rather than by a single conserved pan-cancer module. Within this framework, channels such as KCa3.1, Kir2.1, and TASK-1 are consistently associated with interaction neighbourhoods enriched for membrane-proximal scaffolding, junctional components, cytoskeletal regulators, and vesicular trafficking machinery. Intersection analyses further suggest that overlap between these channels is frequently captured at the level of modular organization (e.g., endocytosis, junctional organization, Hippo-related interfaces) rather than through extensive identity of individual interactors.

These results describe organizational aspects rather than deterministic functional hierarchies. For example, the shared KCa3.1-Kir2.1 network is enriched for junction- and trafficking-associated terms and exhibits a strong membrane/junction localization bias, consistent with a scaffold-like organization of channel-proximal environments. Similarly, the KCa3.1-TASK-1 overlap is strongly represented by vesicle transport and endocytosis modules, compatible with architectural coupling between membrane trafficking and membrane-associated cytoskeletal organization. Collectively, these patterns support the concept that convergence, when detectable, may be encoded by partially shared architectural principles, such as compartmentalization, modularity, scaffold density, and trafficking coupling, rather than by uniform gene programs.

In contrast to these channels, others exhibit interaction landscapes that appear more constrained by compartment-specific regulatory bottlenecks. The Kv11.1 (hERG) interactome shows strong enrichment for ER proteostasis and quality-control machinery, compatible with a regulatory regime dominated by folding surveillance, ER retention, and ERAD-linked pathways. In this context, reduced representation of membrane-proximal scaffolds may reflect limited steady-state residence in signalling-competent compartments in the analysed system, although transient signalling interactions under alternative conditions cannot be excluded. Accordingly, Kv11.1 should be considered a channel whose interactome readout is highly sensitive to trafficking state and proteostasis load, and therefore particularly dependent on cellular context and experimental configuration.

A distinct organizational regime is suggested for Kv1.3, where multiple datasets converge on a mitochondria-enriched core embedded within broader cellular modules. Notably, recent comparative interactome analyses performed in immune-cell systems have similarly shown that Kv1.3 operates within context-dependent protein networks that differ substantially between microglia and T cells, supporting the view that Kv1.3 function is embedded within adaptable organizational architectures rather than fixed sets of molecular interactors [57,58], Cross-method overlap between proximity-labelling and co-immunoprecipitation datasets supports the reproducibility of a subset of mitochondrial and stress-response components, consistent with a structured mitochondrial milieu associated with Kv1.3 in several experimental settings. At the same time, the breadth of the recovered networks, including trafficking, cytoskeletal, RNA metabolism, and proteostasis-related proteins, highlights a known limitation of high-depth interactomics: abundant and highly connected proteins may contribute to apparent convergence without necessarily encoding channel-specific regulation. Consequently, the mitochondrial “bottleneck” is best interpreted as a network-level organizational bias within composite networks rather than as a single invariant pathway.

Recent proximity-labelling studies performed in immune-cell contexts have identified Kv1.3-associated networks enriched at the plasma membrane and immune synapse [57,58], whereas several cancer-derived datasets converge on a mitochondria-enriched Kv1.3 environment [21,22]. These apparent differences likely reflect a combination of biological context, experimental design, proximity-labelling strategy, and statistical filtering. Therefore, the proposed mitochondrial bottleneck should be interpreted as a reproducible but context-dependent organizational tendency rather than as a universal property of the channel.

Accordingly, Kv1.3-associated networks likely reflect a composite organization in which mitochondrial, membrane-proximal, and trafficking-related modules are differentially emphasized depending on cellular context, expression level, metabolic state, and experimental design. This interpretation is consistent with recent evidence demonstrating extensive cell type- and activation state-dependent remodeling of Kv1.3 interactomes across immune systems, reinforcing the concept that channel-associated functions emerge from dynamic network organization rather than invariant interaction repertoires [57,58]. Within this framework, the proposed mitochondrial “bottleneck” represents a reproducible but context-sensitive architectural weighting rather than an exclusive or obligatory signalling configuration. In this perspective, integrating gene-level analyses with comparative interactome architecture may provide a more coherent framework for interpreting the heterogeneous roles of K^+^ channels across tumour contexts and related disease settings.

The present study distinguishes experimentally validated functional interactions from proximity-derived associations and architecture-level inferences. While interactome analyses can identify reproducible organizational principles, mechanistic validation remains essential to establish causal relationships between specific interactors and channel-dependent phenotypes.

In summary, the present study indicates that the functional heterogeneity of cancer-associated K^+^ channels is more effectively understood through interactome architecture than through gene-level features alone. Rather than converging on a single oncogenic programme, distinct channels appear to organize recurrent but context-dependent network architectures that shape signalling, trafficking, adhesion, proteostasis, and metabolic regulation. These findings establish interactome organization as a relevant level of biological interpretation and provide a framework for future mechanistic and therapeutic investigations of K^+^ channel function in cancer.

## Figures and Tables

**Table 1:** Comparative overview of K^+^ channel interactomes included in this study. Summary of the principal characteristics of the interactome datasets analyzed in this work, including channel family, experimental strategy, biological context, major subcellular localization, and dominant interactome architecture. The dominant architectures represent higher-order organizational frameworks inferred from published interactome datasets and subsequent comparative network analyses. Together, these interactomes define distinct organizational paradigms ranging from membrane-associated signalling scaffolds and trafficking-centered networks to proteostasis-dominated and mitochondria-enriched architectures.

Channel (*Gene*)	Family	Experimental Approach	Biological Context	Major Subcellular Localization	Dominant Interactome Architecture	Ref.
Kv11.1 (*KCNH2*/*hERG*)	Voltage-gated K^+^ channel	AP-MS	HEK293 cells	ER, secretory pathway	Proteostasis-centered ER architecture dominated by folding and trafficking control	[12]
TASK-1 (*KCNK3*)	Two-pore domain K^+^ channel	APEX2, TurboID	Pulmonary vascular cells (validation in HEK293 cells)	Caveolae, ER, mitochondria	Multi-compartment bridge architecture integrating trafficking, signalling, and membrane organization	[14]
KCa3.1 (*KCNN4*)	Ca^2+^-activated K^+^ channel	TurboID	PDAC KPC cells	Plasma membrane, focal adhesions, mitochondria	Ca^2+^-anchored peripheral signalling scaffold integrating adhesion and trafficking modules	[15]
Kir2.1 (*KCNJ2*)	Inward rectifier K^+^ channel	BioID	HEK293 Flp-In T-REx cells	Cell–cell junctions, cortical cytoskeleton	Junction-centered, trafficking-sensitive architecture	[16]
Kir1.1 (*ROMK*/*KCNJ1*)	Inward rectifier K^+^ channel	TurboID, Co-IP	HEK293 cells	ER, vesicular compartments, minor mitochondrial localization	Lipid kinase–anchored, compartmentalized architecture regulated by local anionic lipid synthesis	[17]
Kv1.3 (*KCNA3*)	Voltage-gated K^+^ channel	BioID	HEK293 cells	Plasma membrane, mitochondria	Mitochondrial bottleneck linking metabolism to transcriptional regulation	[19]

**Table 2:** STRING-based k-means clustering of the shared interactome between KCa3.1 (*KCNN4*) and TASK-1 (*KCNK3*). Protein–protein interaction network clustering was performed using STRING k-means clustering on proteins shared between the KCa3.1 and TASK-1 interactomes. Clusters represent groups of proteins with coherent interaction patterns and related biological functions. The identified modules highlight convergence in vesicular trafficking, membrane organization, cytoskeletal regulation, and Ca^2+^-associated signalling pathways. Together, these clusters support the existence of a shared membrane-proximal organizational framework integrating trafficking, scaffold-mediated signalling, and cytoskeletal dynamics.

Cluster Number	Cluster Color	Gene Count	Protein Name	Protein Description
1	Red	36	ABI1	Abl interactor 1; May act in negative regulation of cell growth and transformation by interacting with nonreceptor tyrosine kinases ABL1 and/or ABL2. May play a role in regulation of EGF-induced Erk pathway activation. Involved in cytoskeletal reorganization and EGFR signaling. Together with EPS8 participates in transduction of signals from Ras to Rac. In vitro, a trimeric complex of ABI1, EPS8 and SOS1 exhibits Rac specific guanine nucleotide exchange factor (GEF) activity and ABI1 seems to act as an adapter in the complex. Regulates ABL1/c-Abl-mediated phosphorylation of ENAH.
1	Red	36	ANKLE2	Ankyrin repeat and LEM domain-containing protein 2; Involved in mitotic nuclear envelope reassembly by promoting dephosphorylation of BAF/BANF1 during mitotic exit. Coordinates the control of BAF/BANF1 dephosphorylation by inhibiting VRK1 kinase and promoting dephosphorylation of BAF/BANF1 by protein phosphatase 2A (PP2A), thereby facilitating nuclear envelope assembly. It is unclear whether it acts as a real PP2A regulatory subunit or whether it is involved in recruitment of the PP2A complex. Involved in brain development; Belongs to the ANKLE2 family.
1	Red	36	CAPZB	F-actin-capping protein subunit beta; F-actin-capping proteins bind in a Ca^2+^-independent manner to the fast growing ends of actin filaments (barbed end) thereby blocking the exchange of subunits at these ends. Unlike other capping proteins (such as gelsolin and severin), these proteins do not sever actin filaments. Plays a role in the regulation of cell morphology and cytoskeletal organization.
1	Red	36	CDKAL1	Threonylcarbamoyladenosine tRNA methylthiotransferase; Catalyzes the methylthiolation of N6-threonylcarbamoyladenosine (t(6)A), leading to the formation of 2-methylthio-N6-threonylcarbamoyladenosine (ms(2)t(6)A) at position 37 in tRNAs that read codons beginning with adenine. Belongs to the methylthiotransferase family. CDKAL1 subfamily.
1	Red	36	CTNNB1	Catenin beta-1; Key downstream component of the canonical Wnt signaling pathway. In the absence of Wnt, forms a complex with AXIN1, AXIN2, APC, CSNK1A1 and GSK3B that promotes phosphorylation on N-terminal Ser and Thr residues and ubiquitination of CTNNB1 via BTRC and its subsequent degradation by the proteasome. In the presence of Wnt ligand, CTNNB1 is not ubiquitinated and accumulates in the nucleus, where it acts as a coactivator for transcription factors of the TCF/LEF family, leading to activate Wnt responsive genes. Involved in the regulation of cell adhesion.
1	Red	36	CTNND1	Catenin delta-1; Key regulator of cell–cell adhesion that associates with and regulates the cell adhesion properties of both C-, E- and N-cadherins, being critical for their surface stability. Beside cell–cell adhesion, regulates gene transcription through several transcription factors including ZBTB33/Kaiso2 and GLIS2, and the activity of Rho family GTPases and downstream cytoskeletal dynamics. Implicated both in cell transformation by SRC and in ligand-induced receptor signaling through the EGF, PDGF, CSF-1 and ERBB2 receptors. Belongs to the beta-catenin family.
1	Red	36	CXADR	Coxsackievirus and adenovirus receptor; Component of the epithelial apical junction complex that may function as a homophilic cell adhesion molecule and is essential for tight junction integrity. Also involved in transepithelial migration of leukocytes through adhesive interactions with JAML a transmembrane protein of the plasma membrane of leukocytes. The interaction between both receptors also mediates the activation of gamma-delta T-cells, a subpopulation of T-cells residing in epithelia and involved in tissue homeostasis and repair.
1	Red	36	CYP51A1	Lanosterol 14-alpha demethylase; A cytochrome P450 monooxygenase involved in sterol biosynthesis. Catalyzes 14-alpha demethylation of lanosterol and 24,25-dihydrolanosterol likely through sequential oxidative conversion of 14-alpha methyl group to hydroxymethyl, then to carboxylaldehyde, followed by the formation of the delta 14,15 double bond in the sterol core and concomitant release of formic acid.
1	Red	36	DSG2	Desmoglein-2; Component of intercellular desmosome junctions. Involved in the interaction of plaque proteins and intermediate filaments mediating cell–cell adhesion.
1	Red	36	EMD	Emerin; Stabilizes and promotes the formation of a nuclear actin cortical network. Stimulates actin polymerization in vitro by binding and stabilizing the pointed end of growing filaments. Inhibits beta-catenin activity by preventing its accumulation in the nucleus. Acts by influencing the nuclear accumulation of beta-catenin through a CRM1-dependent export pathway. Links centrosomes to the nuclear envelope via a microtubule association. EMD and BAF are cooperative cofactors of HIV-1 infection. Association of EMD with the viral DNA requires the presence of BAF and viral integrase.
1	Red	36	EPB41	Protein 4.1; Protein 4.1 is a major structural element of the erythrocyte membrane skeleton. It plays a key role in regulating membrane physical properties of mechanical stability and deformability by stabilizing spectrin-actin interaction. Recruits DLG1 to membranes. Required for dynein-dynactin complex and NUMA1 recruitment at the mitotic cell cortex during anaphase.
1	Red	36	EPB41L2	Band 4.1-like protein 2; Required for dynein-dynactin complex and NUMA1 recruitment at the mitotic cell cortex during anaphase.
1	Red	36	HGS	Hepatocyte growth factor-regulated tyrosine kinase substrate; Involved in intracellular signal transduction mediated by cytokines and growth factors. When associated with STAM, it suppresses DNA signaling upon stimulation by IL-2 and GM-CSF. Could be a direct effector of PI3-kinase in vesicular pathway via early endosomes and may regulate trafficking to early and late endosomes by recruiting clathrin. May concentrate ubiquitinated receptors within clathrin-coated regions.
1	Red	36	IARS2	Isoleucine-tRNA ligase, mitochondrial; isoleucyl-tRNA synthetase 2, mitochondrial.
1	Red	36	IGF2R	Cation-independent mannose-6-phosphate receptor; Transport of phosphorylated lysosomal enzymes from the Golgi complex and the cell surface to lysosomes. Lysosomal enzymes bearing phosphomannosyl residues bind specifically to mannose-6-phosphate receptors in the Golgi apparatus and the resulting receptor-ligand complex is transported to an acidic prelyosomal compartment where the low pH mediates the dissociation of the complex. This receptor also binds IGF2. Acts as a positive regulator of T-cell coactivation, by binding DPP4.
1	Red	36	IQGAP1	Ras GTPase-activating-like protein IQGAP1; Plays a crucial role in regulating the dynamics and assembly of the actin cytoskeleton. Binds to activated CDC42 but does not stimulate its GTPase activity. It associates with calmodulin. Could serve as an assembly scaffold for the organization of a multimolecular complex that would interface incoming signals to the reorganization of the actin cytoskeleton at the plasma membrane. May promote neurite outgrowth. May play a possible role in cell cycle regulation by contributing to cell cycle progression after DNA replication arrest.
1	Red	36	ITGB1	Integrin beta-1; Integrins alpha-1/beta-1, alpha-2/beta-1, alpha-10/beta-1 and alpha-11/beta-1 are receptors for collagen. Integrins alpha-1/beta-1 and alpha-2/beta-2 recognize the proline-hydroxylated sequence G-F-P-G-E-R in collagen. Integrins alpha-2/beta-1, alpha-3/beta-1, alpha-4/beta-1, alpha-5/beta-1, alpha-8/beta-1, alpha-10/beta-1, alpha-11/beta-1 and alpha-V/beta-1 are receptors for fibronectin. Alpha-4/beta-1 recognizes one or more domains within the alternatively spliced CS-1 and CS-5 regions of fibronectin. Integrin alpha-5/beta-1 is a receptor for fibrinogen.
1	Red	36	JUP	Junction plakoglobin; Common junctional plaque protein. The membrane-associated plaques are architectural elements in an important strategic position to influence the arrangement and function of both the cytoskeleton and the cells within the tissue. The presence of plakoglobin in both the desmosomes and in the intermediate junctions suggests that it plays a central role in the structure and function of submembranous plaques. Acts as a substrate for VE-PTP and is required by it to stimulate VE-cadherin function in endothelial cells.
1	Red	36	LBR	Delta(14)-sterol reductase LBR; Catalyzes the reduction of the C14-unsaturated bond of lanosterol, as part of the metabolic pathway leading to cholesterol biosynthesis. Plays a critical role in myeloid cell cholesterol biosynthesis which is essential to both myeloid cell growth and functional maturation (By similarity). Mediates the activation of NADPH oxidases, perhaps by maintaining critical levels of cholesterol required for membrane lipid raft formation during neutrophil differentiation (By similarity). Anchors the lamina and the heterochromatin to the inner nuclear membrane.
1	Red	36	LEMD3	Inner nuclear membrane protein Man1; Can function as a specific repressor of TGF-beta, activin, and BMP signaling through its interaction with the R-SMAD proteins. Antagonizes TGF-beta-induced cell proliferation arrest.
1	Red	36	LRRC59	Leucine-rich repeat-containing protein 59, N-terminally processed; Required for nuclear import of FGF1, but not that of FGF2. Might regulate nuclear import of exogenous FGF1 by facilitating interaction with the nuclear import machinery and by transporting cytosolic FGF1 to, and possibly through, the nuclear pores.
1	Red	36	MTDH	Protein LYRIC; Downregulates SLC1A2/EAAT2 promoter activity when expressed ectopically. Activates the nuclear factor kappa-B (NF-kappa-B) transcription factor. Promotes anchorage-independent growth of immortalized melanocytes and astrocytes which is a key component in tumor cell expansion. Promotes lung metastasis and also has an effect on bone and brain metastasis, possibly by enhancing the seeding of tumor cells to the target organ endothelium. Induces chemoresistance.
1	Red	36	NOTCH2	Neurogenic locus notch homolog protein 2; Functions as a receptor for membrane-bound ligands Jagged-1 (JAG1), Jagged-2 (JAG2) and Delta-1 (DLL1) to regulate cell-fate determination. Upon ligand activation through the released notch intracellular domain (NICD) it forms a transcriptional activator complex with RBPJ/RBPSUH and activates genes of the enhancer of split locus. Affects the implementation of differentiation, proliferation and apoptotic programs (By similarity). Involved in bone remodeling and homeostasis.
1	Red	36	PGRMC2	Membrane-associated progesterone receptor component 2; Receptor for steroids.
1	Red	36	POR	NADPH--cytochrome P450 reductase; This enzyme is required for electron transfer from NADP to cytochrome P450 in microsomes. It can also provide electron transfer to heme oxygenase and cytochrome B5; Belongs to the NADPH--cytochrome P450 reductase family. In the C-terminal section; belongs to the flavoprotein pyridine nucleotide cytochrome reductase family.
1	Red	36	SCRIB	Protein scribble homolog; Scaffold protein involved in different aspects of polarized cells differentiation regulating epithelial and neuronal morphogenesis. Most probably functions in the establishment of apico-basal cell polarity. May function in cell proliferation regulating progression from G1 to S phase and as a positive regulator of apoptosis for instance during acinar morphogenesis of the mammary epithelium. May also function in cell migration and adhesion and hence regulate cell invasion through MAPK signaling. May play a role in exocytosis and in the targeting synaptic vesicle.
1	Red	36	SLC3A2	4F2 cell-surface antigen heavy chain; Component of several heterodimeric amino acid transporter complexes. The precise substrate specificity depends on the other subunit in the heterodimer. The heterodimer with SLC3A2 functions as sodium-independent, high-affinity transporter that mediates uptake of large neutral amino acids such as phenylalanine, tyrosine, L-DOPA, leucine, histidine, methionine and tryptophan. The complexes with SLC7A6 and SLC7A7 mediate uptake of dibasic amino acids. The complexes function as amino acid exchangers.
1	Red	36	SLK	STE20-like serine/threonine-protein kinase; Mediates apoptosis and actin stress fiber dissolution.
1	Red	36	SPTAN1	Spectrin alpha chain, non-erythrocytic 1; Fodrin, which seems to be involved in secretion, interacts with calmodulin in a calcium-dependent manner and is thus candidate for the calcium-dependent movement of the cytoskeleton at the membrane.
1	Red	36	SPTBN1	Spectrin beta chain, non-erythrocytic 1; Fodrin, which seems to be involved in secretion, interacts with calmodulin in a calcium-dependent manner and is thus candidate for the calcium-dependent movement of the cytoskeleton at the membrane.
1	Red	36	SUCLA2	Succinate--CoA ligase [ADP-forming] subunit beta, mitochondrial; ATP-specific succinyl-CoA synthetase functions in the citric acid cycle (TCA), coupling the hydrolysis of succinyl-CoA to the synthesis of ATP and thus represents the only step of substrate-level phosphorylation in the TCA. The beta subunit provides nucleotide specificity of the enzyme and binds the substrate succinate, while the binding sites for coenzyme A and phosphate are found in the alpha subunit (By similarity).
1	Red	36	TP53BP2	Apoptosis-stimulating of p53 protein 2; Regulator that plays a central role in regulation of apoptosis and cell growth via its interactions with proteins such as TP53. Regulates TP53 by enhancing the DNA binding and transactivation function of TP53 on the promoters of proapoptotic genes in vivo. Inhibits the ability of APPBP1 to conjugate NEDD8 to CUL1, and thereby decreases APPBP1 ability to induce apoptosis. Impedes cell cycle progression at G2/M. Its apoptosis-stimulating activity is inhibited by its interaction with DDX42.
1	Red	36	TSG101	Tumor susceptibility gene 101 protein; Component of the ESCRT-I complex, a regulator of vesicular trafficking process. Binds to ubiquitinated cargo proteins and is required for the sorting of endocytic ubiquitinated cargos into multivesicular bodies (MVBs). Mediates the association between the ESCRT-0 and ESCRT-I complex. Required for completion of cytokinesis; the function requires CEP55. May be involved in cell growth and differentiation. Acts as a negative growth regulator. I
1	Red	36	UTRN	Utrophin; May play a role in anchoring the cytoskeleton to the plasma membrane.
1	Red	36	VEZT	Vezatin; Plays a pivotal role in the establishment of adherens junctions and their maintenance in adult life. Required for morphogenesis of the preimplantation embryo, and for the implantation process; Belongs to the vezatin family.
1	Red	36	ZFYVE16	Zinc finger FYVE domain-containing protein 16; May be involved in regulating membrane trafficking in the endosomal pathway. Overexpression induces endosome aggregation. Required to target TOM1 to endosomes.
2	Yellow	21	CAMK2D	Calcium/calmodulin-dependent protein kinase type II subunit delta; Calcium/calmodulin-dependent protein kinase involved in the regulation of Ca^2+^ homeostatis and excitation-contraction coupling (ECC) in heart by targeting ion channels, transporters and accessory proteins involved in Ca^2+^ influx into the myocyte, Ca^2+^ release from the sarcoplasmic reticulum (SR), SR Ca^2+^ uptake and Na^+^ and K^+^ channel transport. Targets also transcription factors and signaling molecules to regulate heart function. In its activated form, is involved in the pathogenesis of dilated cardiomyopath.
2	Yellow	21	CAMLG	Calcium signal-modulating cyclophilin ligand; Likely involved in the mobilization of calcium as a result of the TCR/CD3 complex interaction. Binds to cyclophilin B.
2	Yellow	21	EIF4H	Eukaryotic translation initiation factor 4H; Stimulates the RNA helicase activity of EIF4A in the translation initiation complex. Binds weakly mRNA.
2	Yellow	21	GOLGA3	Golgin subfamily A member 3; Golgi auto-antigen; probably involved in maintaining Golgi structure.
2	Yellow	21	MIA2	Melanoma inhibitory activity protein 2; Plays a role in the transport of cargos that are too large to fit into COPII-coated vesicles and require specific mechanisms to be incorporated into membrane-bound carriers and exported from the endoplasmic reticulum. Plays a role in the secretion of lipoproteins, pre-chylomicrons and pre-VLDLs, by participating in their export from the endoplasmic reticulum. Thereby, may play a role in cholesterol and triglyceride homeostasis (By similarity).
2	Yellow	21	MIA3	Transport and Golgi organization protein 1 homolog; Plays a role in the transport of cargos that are too large to fit into COPII-coated vesicles and require specific mechanisms to be incorporated into membrane-bound carriers and exported from the endoplasmic reticulum. This protein is required for collagen VII (COL7A1) secretion by loading COL7A1 into transport carriers. It may participate in cargo loading of COL7A1 at endoplasmic reticulum exit sites by binding to COPII coat subunits Sec23/24 and guiding SH3-bound COL7A1 into a growing carrier.
2	Yellow	21	SCFD1	Sec1 family domain-containing protein 1; Plays a role in SNARE-pin assembly and Golgi-to-ER retrograde transport via its interaction with COG4. Involved in vesicular transport between the endoplasmic reticulum and the Golgi (By similarity).
2	Yellow	21	SEC22B	Vesicle-trafficking protein SEC22b; SNARE involved in targeting and fusion of ER-derived transport vesicles with the Golgi complex as well as Golgi-derived retrograde transport vesicles with the ER. Belongs to the synaptobrevin family.
2	Yellow	21	SEC23IP	SEC23-interacting protein; Plays a role in the organization of endoplasmic reticulum exit sites. Specifically binds to phosphatidylinositol 3-phosphate (PI(3)P), phosphatidylinositol 4-phosphate (PI(4)P) and phosphatidylinositol 5-phosphate (PI(5)P). Belongs to the PA-PLA1 family.
2	Yellow	21	SEC24A	Protein transport protein Sec24A; Component of the coat protein complex II (COPII) which promotes the formation of transport vesicles from the endoplasmic reticulum (ER). The coat has two main functions, the physical deformation of the endoplasmic reticulum membrane into vesicles and the selection of cargo molecules for their transport to the Golgi complex. Plays a central role in cargo selection within the COPII complex and together with SEC24B may have a different specificity compared to SEC24C and SEC24D.
2	Yellow	21	SEC24B	Protein transport protein Sec24B; Component of the coat protein complex II (COPII) which promotes the formation of transport vesicles from the endoplasmic reticulum (ER). The coat has two main functions, the physical deformation of the endoplasmic reticulum membrane into vesicles and the selection of cargo molecules for their transport to the Golgi complex. Plays a central role in cargo selection within the COPII complex and together with SEC24A may have a different specificity compared to SEC24C and SEC24D.
2	Yellow	21	SNAP47	Synaptosomal-associated protein 47; Plays a role in intracellular membrane fusion.
2	Yellow	21	STIM1	Stromal interaction molecule 1; Plays a role in mediating store-operated Ca^2+^ entry (SOCE), a Ca^2+^ influx following depletion of intracellular Ca^2+^ stores. Acts as Ca^2+^ sensor in the endoplasmic reticulum via its EF-hand domain. Upon Ca^2+^ depletion, translocates from the endoplasmic reticulum to the plasma membrane where it activates the Ca^2+^ release-activated Ca^2+^ (CRAC) channel subunit ORAI1. Involved in enamel formation. Activated following interaction with STIMATE, leading to promote STIM1 conformational switch.
2	Yellow	21	STIM2	Stromal interaction molecule 2; Plays a role in mediating store-operated Ca^2+^ entry (SOCE), a Ca^2+^ influx following depletion of intracellular Ca^2+^ stores. Functions as a highly sensitive Ca^2+^ sensor in the endoplasmic reticulum which activates both store-operated and store-independent Ca^2+^-influx. Regulates basal cytosolic and endoplasmic reticulum Ca^2+^ concentrations. Upon mild variations in the endoplasmic reticulum Ca^2+^ concentration, translocates from the endoplasmic reticulum to the plasma membrane where it probably activates the Ca^2+^ release-activated Ca^2+^.
2	Yellow	21	STX18	Syntaxin-18; Syntaxin that may be involved in targeting and fusion of Golgi-derived retrograde transport vesicles with the ER.
2	Yellow	21	STX5	Syntaxin-5; Mediates endoplasmic reticulum to Golgi transport. Together with p115/USO1 and GM130/GOLGA2, involved in vesicle tethering and fusion at the cis-Golgi membrane to maintain the stacked and inter-connected structure of the Golgi apparatus.
2	Yellow	21	STXBP3	Syntaxin-binding protein 3; Together with STX4 and VAMP2, may play a role in insulin-dependent movement of GLUT4 and in docking/fusion of intracellular GLUT4-containing vesicles with the cell surface in adipocytes.
2	Yellow	21	TRIP11	Thyroid receptor-interacting protein 11; Is a membrane tether required for vesicle tethering to Golgi. Has an essential role in the maintenance of Golgi structure and function. It is required for efficient anterograde and retrograde trafficking in the early secretory pathway, functioning at both the ER-to-Golgi intermediate compartment (ERGIC) and Golgi complex. Binds the ligand binding domain of the thyroid receptor (THRB) in the presence of triiodothyronine and enhances THRB-modulated transcription.
2	Yellow	21	USE1	Vesicle transport protein USE1; SNARE that may be involved in targeting and fusion of Golgi-derived retrograde transport vesicles with the ER.
2	Yellow	21	XRN1	5′-3′ exoribonuclease 1; Major 5′-3′ exoribonuclease involved in mRNA decay. Required for the 5′-3′-processing of the G4 tetraplex-containing DNA and RNA substrates. The kinetic of hydrolysis is faster for G4 RNA tetraplex than for G4 DNA tetraplex and monomeric RNA tetraplex. Binds to RNA and DNA (By similarity). Plays a role in replication-dependent histone mRNA degradation. May act as a tumor suppressor protein in osteogenic sarcoma (OGS).
2	Yellow	21	ZC3HAV1	Zinc finger CCCH-type antiviral protein 1; Antiviral protein which inhibits the replication of viruses by recruiting the cellular RNA degradation machineries to degrade the viral mRNAs. Binds to a ZAP-responsive element (ZRE) present in the target viral mRNA, recruits cellular poly(A)-specific ribonuclease PARN to remove the poly(A) tail, and the 3′-5′ exoribonuclease complex exosome to degrade the RNA body from the 3′-end. It also recruits the decapping complex DCP1-DCP2 through RNA helicase p72 (DDX17) to remove the cap structure of the viral mRNA to initiate its degradation.
3	Green	16	AP2A2	AP-2 complex subunit alpha-2; Component of the adaptor protein complex 2 (AP-2). Adaptor protein complexes function in protein transport via transport vesicles in different membrane traffic pathways. Adaptor protein complexes are vesicle coat components and appear to be involved in cargo selection and vesicle formation. AP-2 is involved in clathrin-dependent endocytosis in which cargo proteins are incorporated into vesicles surrounded by clathrin (clathrin-coated vesicles, CCVs) which are destined for fusion with the early endosome.
3	Green	16	AP2B1	AP-2 complex subunit beta; Component of the adaptor protein complex 2 (AP-2). Adaptor protein complexes function in protein transport via transport vesicles in different membrane traffic pathways. Adaptor protein complexes are vesicle coat components and appear to be involved in cargo selection and vesicle formation. AP-2 is involved in clathrin-dependent endocytosis in which cargo proteins are incorporated into vesicles surrounded by clathrin (clathrin-coated vesicles, CCVs) which are destined for fusion with the early endosome.
3	Green	16	CISD2	CDGSH iron-sulfur domain-containing protein 2; Regulator of autophagy that contributes to antagonize BECN1-mediated cellular autophagy at the endoplasmic reticulum. Participates in the interaction of BCL2 with BECN1 and is required for BCL2-mediated depression of endoplasmic reticulum Ca^2+^ stores during autophagy. Contributes to BIK-initiated autophagy, while it is not involved in BIK-dependent activation of caspases. Involved in life span control, probably via its function as regulator of autophagy. Belongs to the CISD protein family. CISD2 subfamily.
3	Green	16	ESYT1	Extended synaptotagmin-1; Binds glycerophospholipids in a barrel-like domain and may play a role in cellular lipid transport (By similarity). Binds calcium (via the C2 domains) and translocates to sites of contact between the endoplasmic reticulum and the cell membrane in response to increased cytosolic calcium levels. Helps tether the endoplasmic reticulum to the cell membrane and promotes the formation of appositions between the endoplasmic reticulum and the cell membrane; Belongs to the extended synaptotagmin family.
3	Green	16	FKBP8	Peptidyl-prolyl cis-trans isomerase FKBP8; Constitutively inactive PPiase, which becomes active when bound to calmodulin and calcium. Seems to act as a chaperone for BCL2, targets it to the mitochondria and modulates its phosphorylation state. The BCL2/FKBP8/calmodulin/calcium complex probably interferes with the binding of BCL2 to its targets. The active form of FKBP8 may therefore play a role in the regulation of apoptosis.
3	Green	16	LSG1	Large subunit GTPase 1 homolog; GTPase required for the XPO1/CRM1-mediated nuclear export of the 60S ribosomal subunit. Probably acts by mediating the release of NMD3 from the 60S ribosomal subunit after export into the cytoplasm (Probable).
3	Green	16	OSBP	Oxysterol-binding protein 1; Lipid transporter involved in lipid countertransport between the Golgi complex and membranes of the endoplasmic reticulum: specifically exchanges sterol with phosphatidylinositol 4-phosphate (PI4P), delivering sterol to the Golgi in exchange for PI4P, which is degraded by the SAC1/SACM1L phosphatase in the endoplasmic reticulum. Binds cholesterol and a range of oxysterols including 25-hydroxycholesterol. Cholesterol binding promotes the formation of a complex with PP2A and a tyrosine phosphatase which dephosphorylates ERK1/2.
3	Green	16	PDZD8	PDZ domain-containing protein 8; Molecular tethering protein that connects endoplasmic reticulum and mitochondria membranes. PDZD8-dependent endoplasmic reticulum-mitochondria membrane tethering is essential for endoplasmic reticulum-mitochondria Ca^2+^ transfer. In neurons, involved in the regulation of dendritic Ca^2+^ dynamics by regulating mitochondrial Ca^2+^ uptake in neurons. Plays an indirect role in the regulation of cell morphology and cytoskeletal organization. May inhibit herpes simplex virus 1 infection at an early stage.
3	Green	16	RAB3GAP1	Rab3 GTPase-activating protein catalytic subunit; Probable catalytic subunit of a GTPase activating protein that has specificity for Rab3 subfamily (RAB3A, RAB3B, RAB3C and RAB3D). Rab3 proteins are involved in regulated exocytosis of neurotransmitters and hormones. Specifically converts active Rab3-GTP to the inactive form Rab3-GDP. Required for normal eye and brain development. May participate in neurodevelopmental processes such as proliferation, migration and differentiation before synapse formation, and non-synaptic vesicular release of neurotransmitters.
3	Green	16	RAB3GAP2	Rab3 GTPase-activating protein non-catalytic subunit; Regulatory subunit of a GTPase activating protein that has specificity for Rab3 subfamily (RAB3A, RAB3B, RAB3C and RAB3D). Rab3 proteins are involved in regulated exocytosis of neurotransmitters and hormones. Rab3 GTPase-activating complex specifically converts active Rab3-GTP to the inactive form Rab3-GDP. Required for normal eye and brain development. May participate in neurodevelopmental processes such as proliferation, migration and differentiation before synapse formation, and non-synaptic vesicular release of neurotransmitters.
3	Green	16	SCYL1	N-terminal kinase-like protein; Regulates COPI-mediated retrograde protein traffic at the interface between the Golgi apparatus and the endoplasmic reticulum. Involved in the maintenance of the Golgi apparatus morphology. Has no detectable kinase activity in vitro.
3	Green	16	SCYL2	SCY1-like protein 2; Component of AP2-containing clathrin coated structures at the plasma membrane or of endocytic coated vesicles. According to probable serine/threonine-protein kinase that phosphorylates, in vitro, the beta2-subunit of the plasma membrane adapter complex AP2 and other proteins in presence of poly-L-lysine. According to has no detectable kinase activity in vitro. May regulate clathrin-dependent trafficking between the TGN and/or the endosomal system.
3	Green	16	STK3	Serine/threonine-protein kinase 3 20 kDa subunit; Stress-activated, pro-apoptotic kinase which, following caspase-cleavage, enters the nucleus and induces chromatin condensation followed by internucleosomal DNA fragmentation. Key component of the Hippo signaling pathway which plays a pivotal role in organ size control and tumor suppression by restricting proliferation and promoting apoptosis.
3	Green	16	UBXN4	UBX domain-containing protein 4; Involved in endoplasmic reticulum-associated protein degradation (ERAD). Acts as a platform to recruit both UBQLN1 and VCP to the ER during ERAD.
3	Green	16	VAPA	Vesicle-associated membrane protein-associated protein A; Binds to OSBPL3, which mediates recruitment of VAPA to plasma membrane sites. The ORP3-VAPA complex stimulates RRAS signaling which in turn attenuates integrin beta-1 (ITGB1) activation at the cell surface. With OSBPL3, may regulate ER morphology. May play a role in vesicle trafficking.
3	Green	16	VAPB	Vesicle-associated membrane protein-associated protein B/C; Participates in the endoplasmic reticulum unfolded protein response (UPR) by inducing ERN1/IRE1 activity. Involved in cellular calcium homeostasis regulation.
4	Blue	2	CPSF3	Cleavage and polyadenylation specificity factor subunit 3; Component of the cleavage and polyadenylation specificity factor (CPSF) complex that play a key role in pre-mRNA 3′-end formation, recognizing the AAUAAA signal sequence and interacting with poly(A) polymerase and other factors to bring about cleavage and poly(A) addition. Has endonuclease activity, and functions as mRNA 3′-end-processing endonuclease. Also involved in the histone 3′-end pre-mRNA processing.
4	Blue	2	NDC1	Nucleoporin NDC1; Component of the nuclear pore complex (NPC), which plays a key role in de novo assembly and insertion of NPC in the nuclear envelope. Required for NPC and nuclear envelope assembly, possibly by forming a link between the nuclear envelope membrane and soluble nucleoporins, thereby anchoring the NPC in the membrane.

**Table 3:** STRING-based k-means clustering of the shared interactome between KCa3.1 (*KCNN4*), Kir2.1 (*KCNJ2*), and TASK-1 (*KCNK3*). Protein–protein interaction network clustering was performed using STRING k-means clustering on proteins present in the triple-channel overlap. Clusters represent groups of proteins with coherent interaction patterns and related biological functions. The identified modules are primarily associated with membrane trafficking, junctional organization, cell polarity, and receptor-associated signalling. Compared with pairwise overlaps, the triple-intersection network retains a more restricted set of highly connected organizational nodes, indicating increased functional stringency and selective preservation of shared architectural features. Together, these clusters define a conserved organizational core linking trafficking, scaffold-mediated compartmentalization, and spatial coordination of signalling processes across multiple K^+^ channel families.

Cluster Number	Cluster Color	Gene Count	Protein Name	Protein Description
1	Red	4	SCFD1	Sec1 family domain-containing protein 1; Plays a role in SNARE-pin assembly and Golgi-to-ER retrograde transport via its interaction with COG4. Involved in vesicular transport between the endoplasmic reticulum and the Golgi (By similarity).
1	Red	4	SEC24A	Protein transport protein Sec24A; Component of the coat protein complex II (COPII) which promotes the formation of transport vesicles from the endoplasmic reticulum (ER). The coat has two main functions, the physical deformation of the endoplasmic reticulum membrane into vesicles and the selection of cargo molecules for their transport to the Golgi complex. Plays a central role in cargo selection within the COPII complex and together with SEC24B may have a different specificity compared to SEC24C and SEC24D.
1	Red	4	SEC24B	Protein transport protein Sec24B; Component of the coat protein complex II (COPII) which promotes the formation of transport vesicles from the endoplasmic reticulum (ER). The coat has two main functions, the physical deformation of the endoplasmic reticulum membrane into vesicles and the selection of cargo molecules for their transport to the Golgi complex. Plays a central role in cargo selection within the COPII complex and together with SEC24A may have a different specificity compared to SEC24C and SEC24D.
1	Red	4	TRIP11	Thyroid receptor-interacting protein 11; Is a membrane tether required for vesicle tethering to Golgi. Has an essential role in the maintenance of Golgi structure and function. It is required for efficient anterograde and retrograde trafficking in the early secretory pathway, functioning at both the ER-to-Golgi intermediate compartment (ERGIC) and Golgi complex. Binds the ligand binding domain of the thyroid receptor (THRB) in the presence of triiodothyronine and enhances THRB-modulated transcription.
2	Green	3	DSG2	Desmoglein-2; Component of intercellular desmosome junctions. Involved in the interaction of plaque proteins and intermediate filaments mediating cell–cell adhesion.
2	Green	3	JUP	Junction plakoglobin; Common junctional plaque protein. The membrane-associated plaques are architectural elements in an important strategic position to influence the arrangement and function of both the cytoskeleton and the cells within the tissue. The presence of plakoglobin in both the desmosomes and in the intermediate junctions suggests that it plays a central role in the structure and function of submembranous plaques. Acts as a substrate for VE-PTP and is required by it to stimulate VE-cadherin function in endothelial cells.
2	Green	3	SCRIB	Protein scribble homolog; Scaffold protein involved in different aspects of polarized cells differentiation regulating epithelial and neuronal morphogenesis. Most probably functions in the establishment of apico-basal cell polarity. May function in cell proliferation regulating progression from G1 to S phase and as a positive regulator of apoptosis for instance during acinar morphogenesis of the mammary epithelium. May also function in cell migration and adhesion and hence regulate cell invasion through MAPK signaling. May play a role in exocytosis and in the targeting synaptic vesicle.
3	Blue	3	HGS	Hepatocyte growth factor-regulated tyrosine kinase substrate; Involved in intracellular signal transduction mediated by cytokines and growth factors. When associated with STAM, it suppresses DNA signaling upon stimulation by IL-2 and GM-CSF. Could be a direct effector of PI3-kinase in vesicular pathway via early endosomes and may regulate trafficking to early and late endosomes by recruiting clathrin. May concentrate ubiquitinated receptors within clathrin-coated regions. Involved in down-regulation of receptor tyrosine kinase via multivesicular body (MVBs) when complexed with STAM.
3	Blue	3	IGF2R	Cation-independent mannose-6-phosphate receptor; Transport of phosphorylated lysosomal enzymes from the Golgi complex and the cell surface to lysosomes. Lysosomal enzymes bearing phosphomannosyl residues bind specifically to mannose-6-phosphate receptors in the Golgi apparatus and the resulting receptor-ligand complex is transported to an acidic prelyosomal compartment where the low pH mediates the dissociation of the complex. This receptor also binds IGF2. Acts as a positive regulator of T-cell coactivation, by binding DPP4.
3	Blue	3	ZFYVE16	Zinc finger FYVE domain-containing protein 16; May be involved in regulating membrane trafficking in the endosomal pathway. Overexpression induces endosome aggregation. Required to target TOM1 to endosomes.

## Data Availability

The data that support the findings of this study are available from the corresponding author upon reasonable request.

## Supplementary Materials

The following supporting information can be downloaded at: https://www.mdpi.com/article/10.3390/ijms27135862/s1.
