# Supplementary material for: Comparative Interactome Analysis Reveals Architectural Principles Governing K+ Channel Function in Cancer
Source: Int J Mol Sci. 2026 Jun 29;27(13):5862. doi: 10.3390/ijms27135862 (PMC13362327; doi:10.3390/ijms27135862)
Supplement: Supplementary file 1 [file ijms-27-05862-s001.zip › 100626OK-SupplFigures.pptx]

## Slide 1
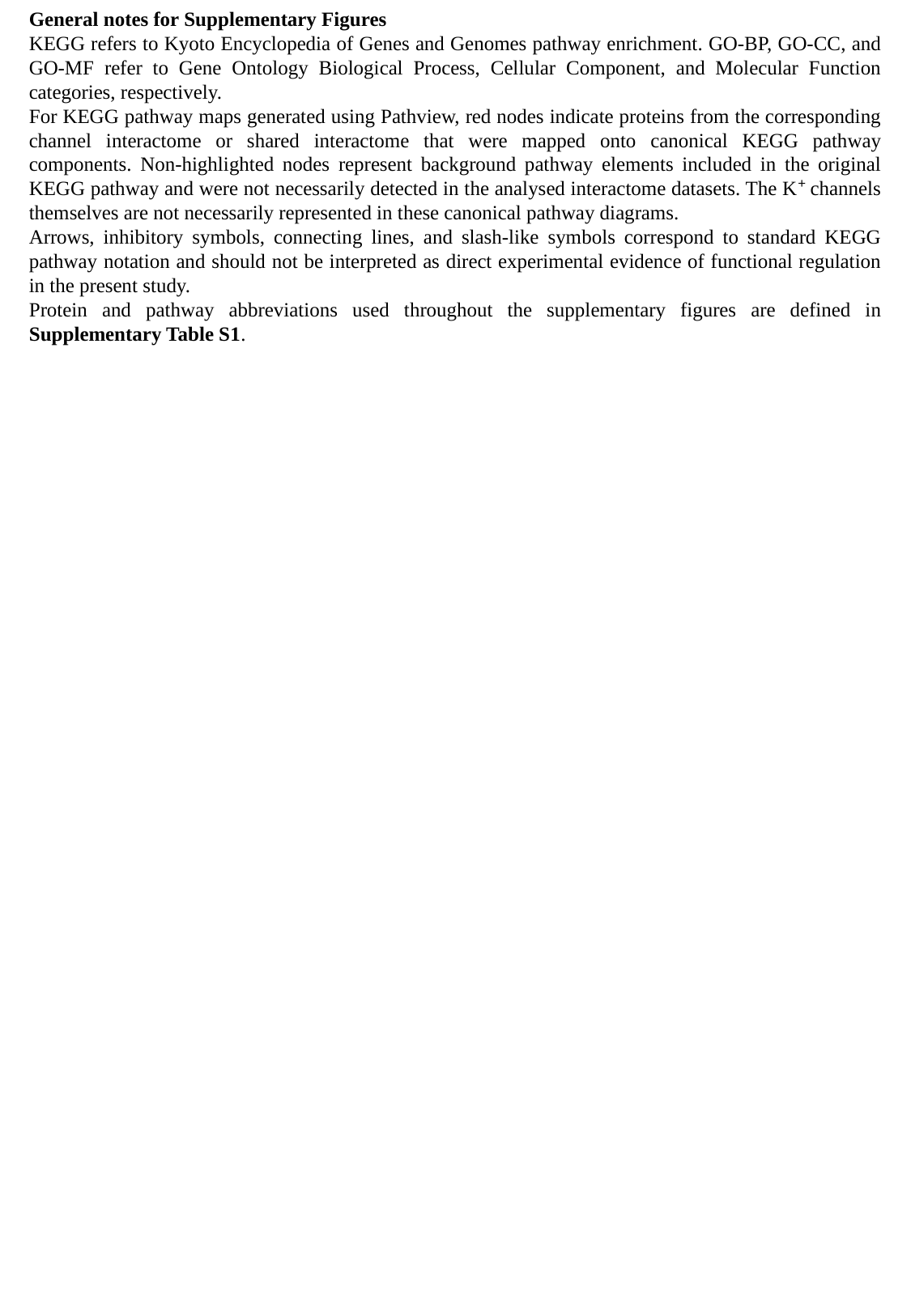

General notes for Supplementary Figures
KEGG refers to Kyoto Encyclopedia of Genes and Genomes pathway enrichment. GO-BP, GO-CC, and GO-MF refer to Gene Ontology Biological Process, Cellular Component, and Molecular Function categories, respectively.
For KEGG pathway maps generated using Pathview, red nodes indicate proteins from the corresponding channel interactome or shared interactome that were mapped onto canonical KEGG pathway components. Non-highlighted nodes represent background pathway elements included in the original KEGG pathway and were not necessarily detected in the analysed interactome datasets. The K⁺ channels themselves are not necessarily represented in these canonical pathway diagrams.
Arrows, inhibitory symbols, connecting lines, and slash-like symbols correspond to standard KEGG pathway notation and should not be interpreted as direct experimental evidence of functional regulation in the present study.
Protein and pathway abbreviations used throughout the supplementary figures are defined in Supplementary Table S1.

## Slide 2
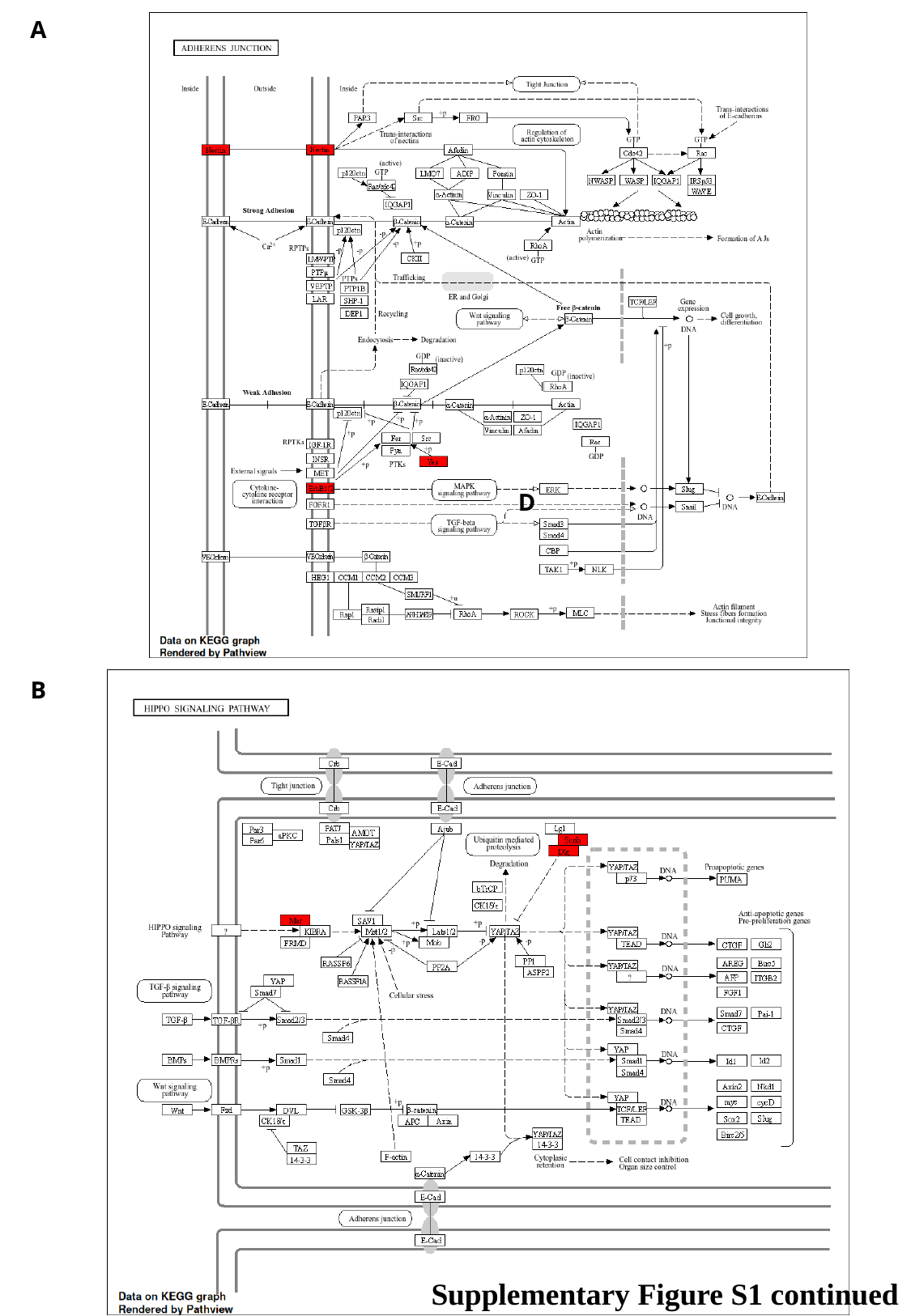

A
D
B
Supplementary Figure S1 continued

## Slide 3
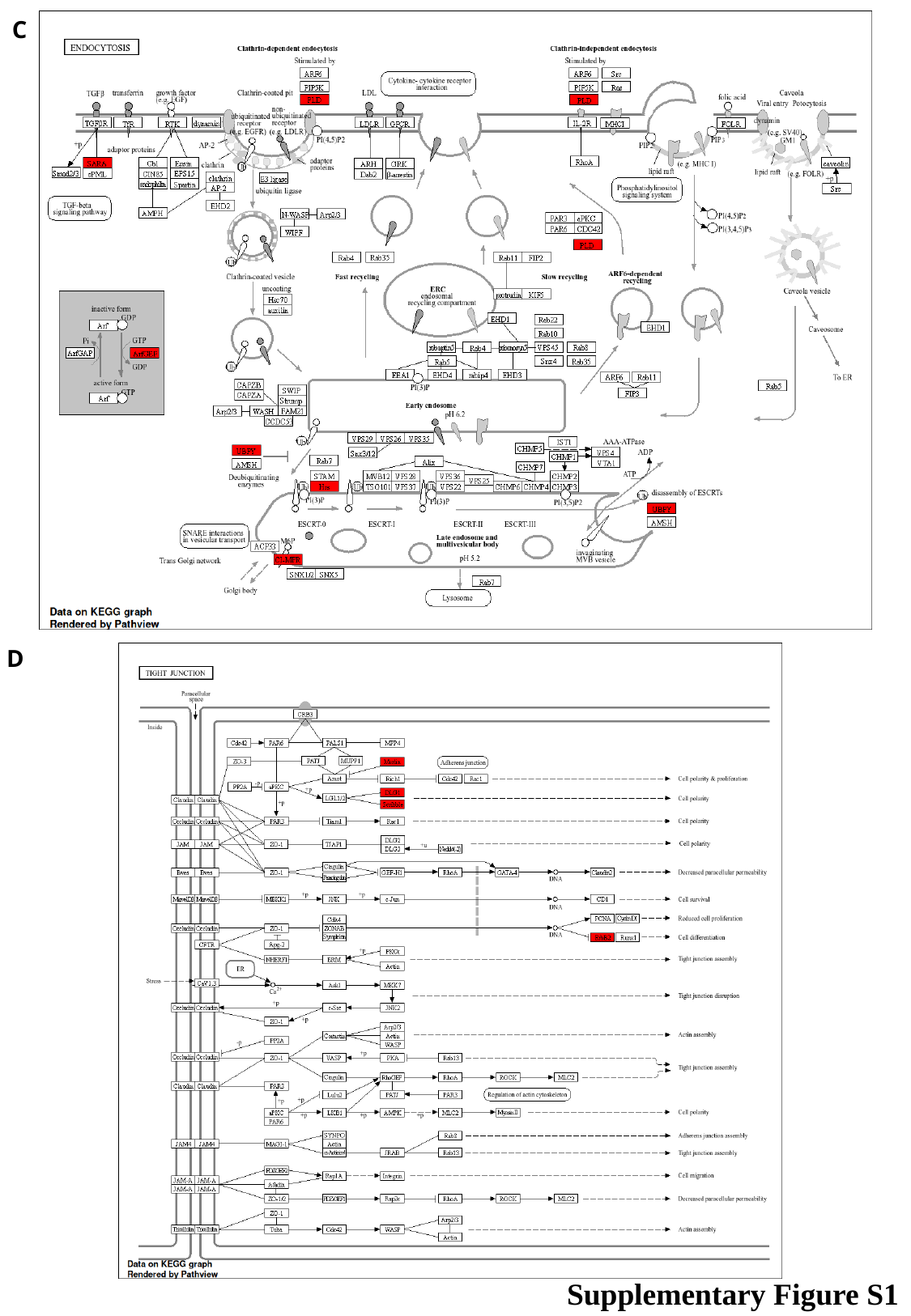

C
D
Supplementary Figure S1

## Slide 4
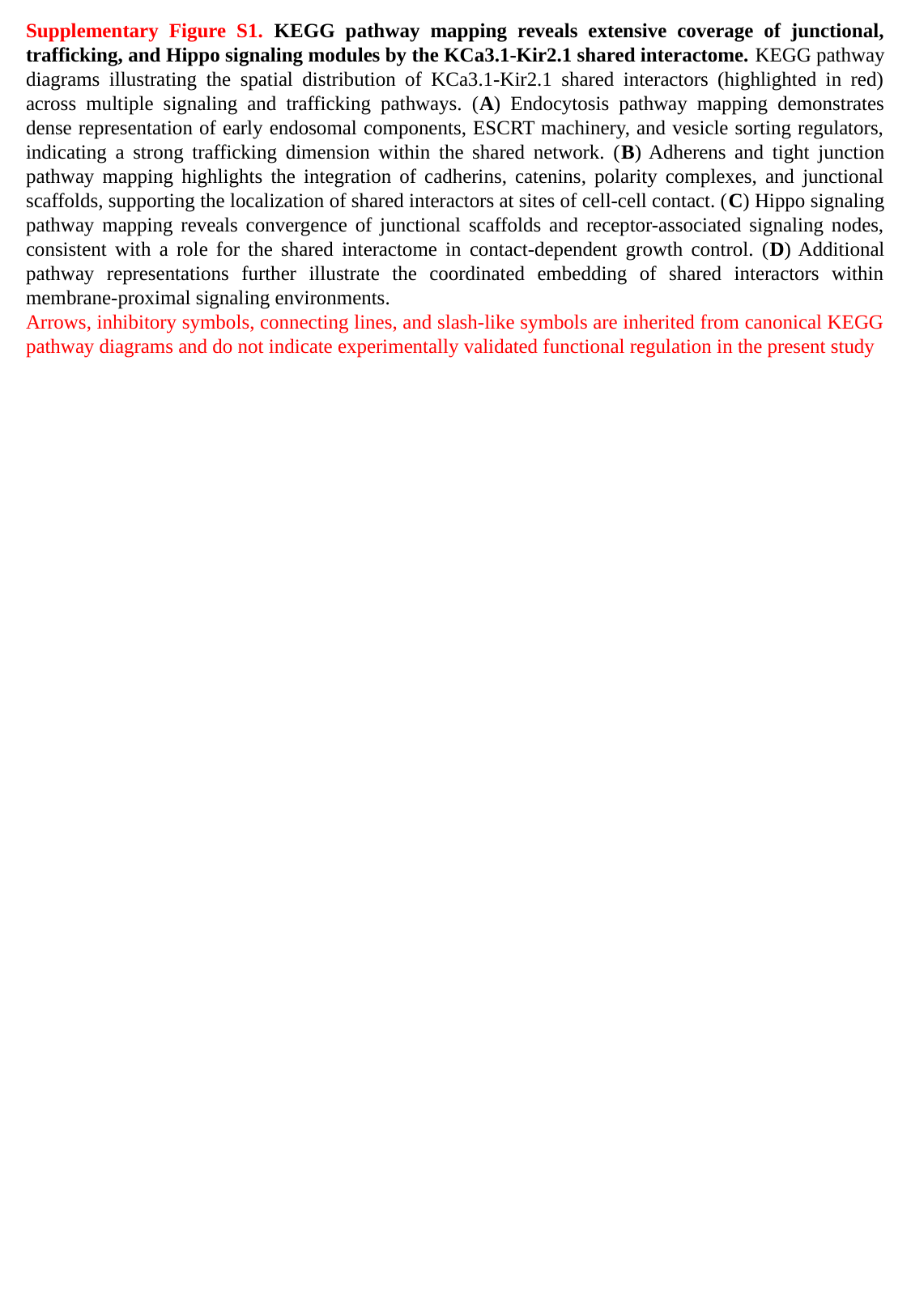

Supplementary Figure S1. KEGG pathway mapping reveals extensive coverage of junctional, trafficking, and Hippo signaling modules by the KCa3.1-Kir2.1 shared interactome. KEGG pathway diagrams illustrating the spatial distribution of KCa3.1-Kir2.1 shared interactors (highlighted in red) across multiple signaling and trafficking pathways. (A) Endocytosis pathway mapping demonstrates dense representation of early endosomal components, ESCRT machinery, and vesicle sorting regulators, indicating a strong trafficking dimension within the shared network. (B) Adherens and tight junction pathway mapping highlights the integration of cadherins, catenins, polarity complexes, and junctional scaffolds, supporting the localization of shared interactors at sites of cell-cell contact. (C) Hippo signaling pathway mapping reveals convergence of junctional scaffolds and receptor-associated signaling nodes, consistent with a role for the shared interactome in contact-dependent growth control. (D) Additional pathway representations further illustrate the coordinated embedding of shared interactors within membrane-proximal signaling environments.
Arrows, inhibitory symbols, connecting lines, and slash-like symbols are inherited from canonical KEGG pathway diagrams and do not indicate experimentally validated functional regulation in the present study

## Slide 5
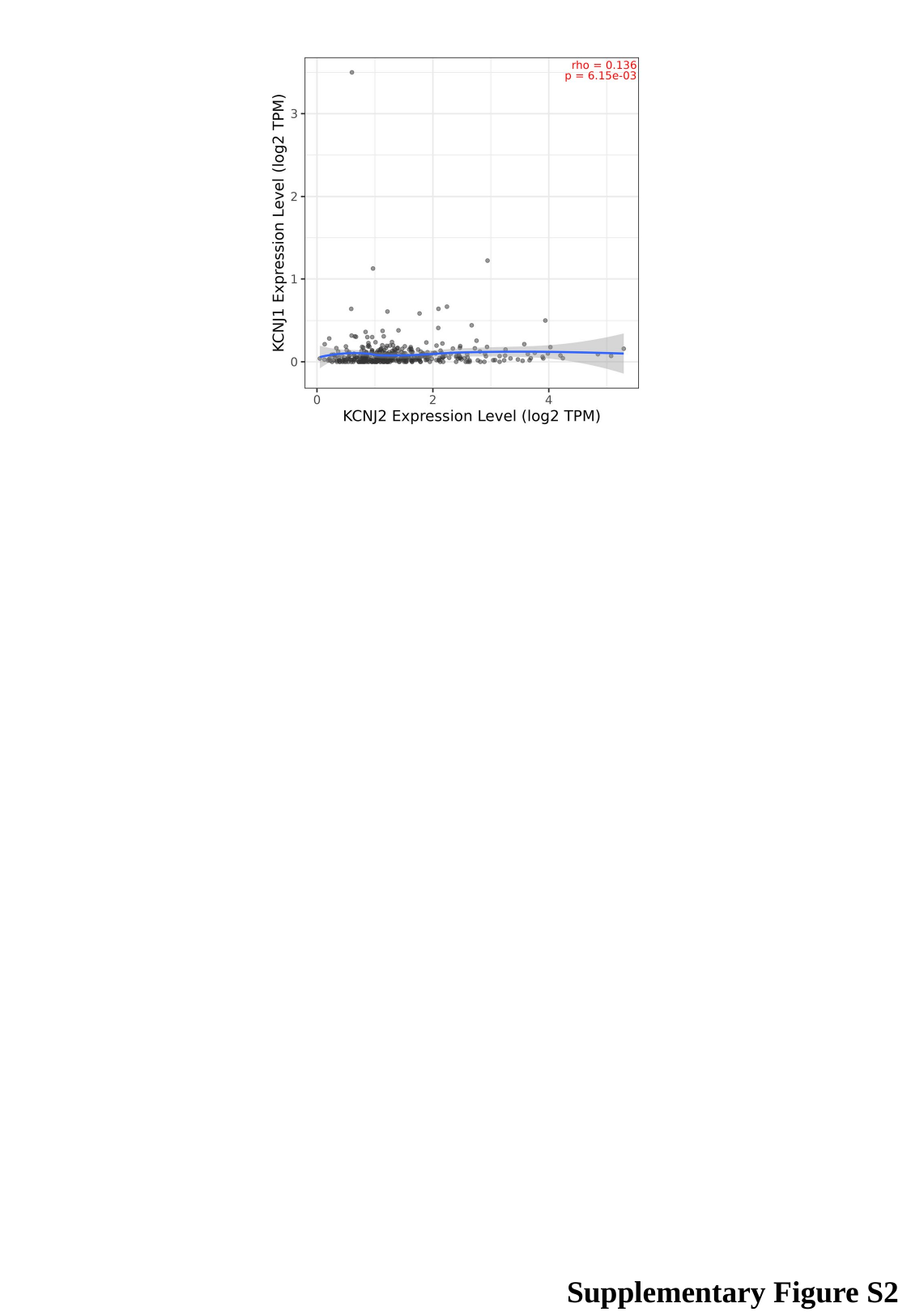

Supplementary Figure S2

## Slide 6
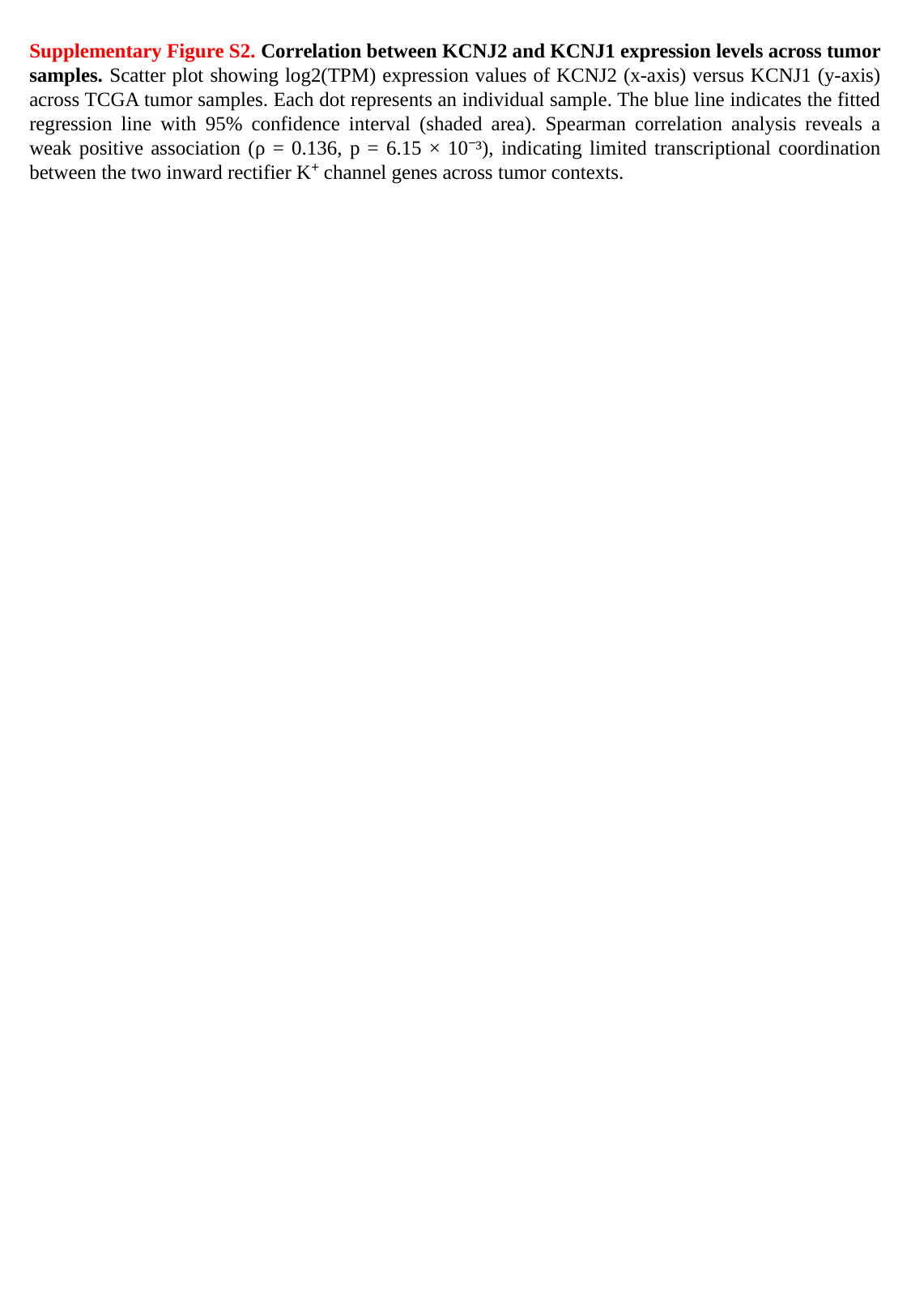

Supplementary Figure S2. Correlation between KCNJ2 and KCNJ1 expression levels across tumor samples. Scatter plot showing log2(TPM) expression values of KCNJ2 (x-axis) versus KCNJ1 (y-axis) across TCGA tumor samples. Each dot represents an individual sample. The blue line indicates the fitted regression line with 95% confidence interval (shaded area). Spearman correlation analysis reveals a weak positive association (ρ = 0.136, p = 6.15 × 10⁻³), indicating limited transcriptional coordination between the two inward rectifier K⁺ channel genes across tumor contexts.

## Slide 7
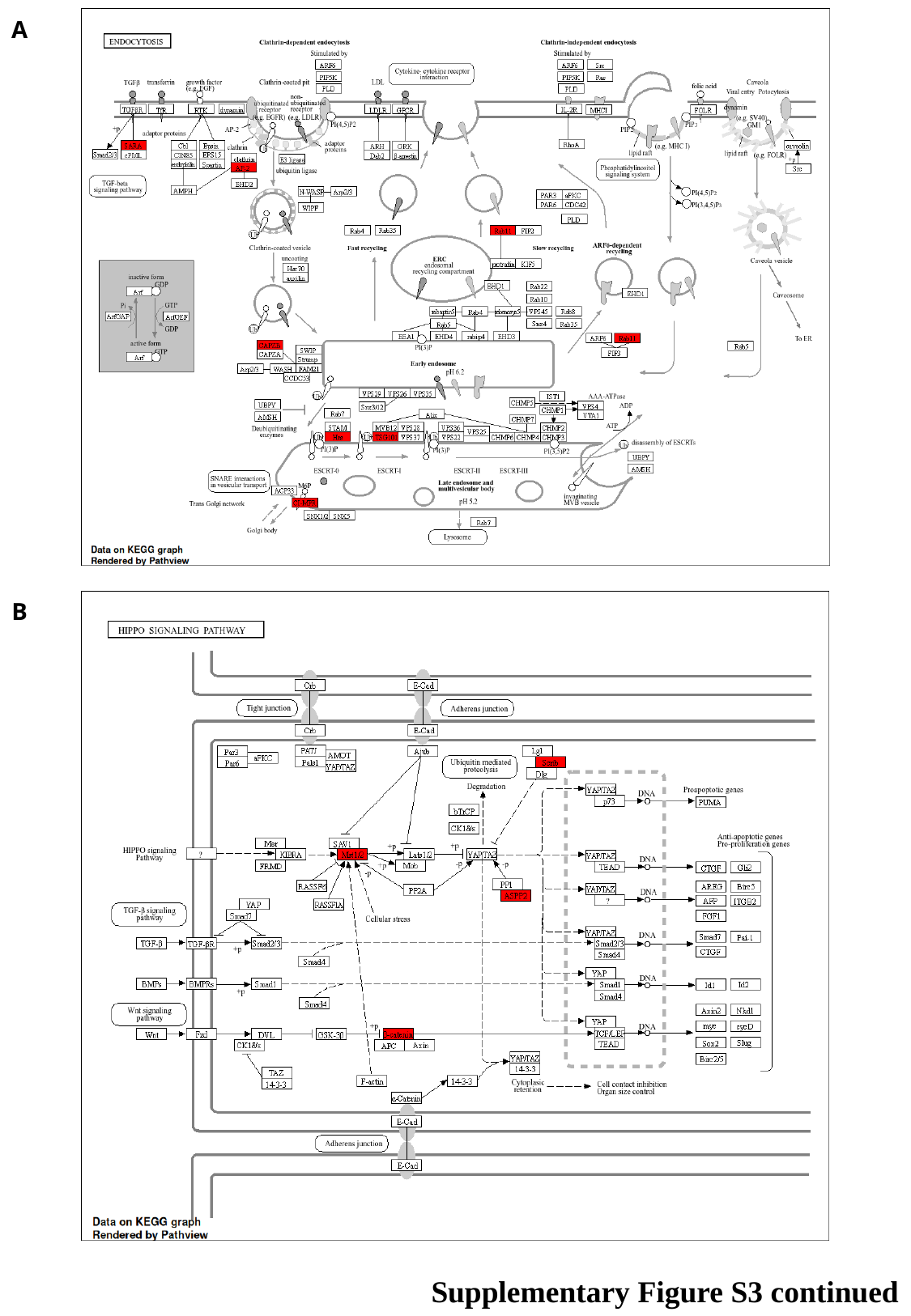

A
B
Supplementary Figure S3 continued

## Slide 8
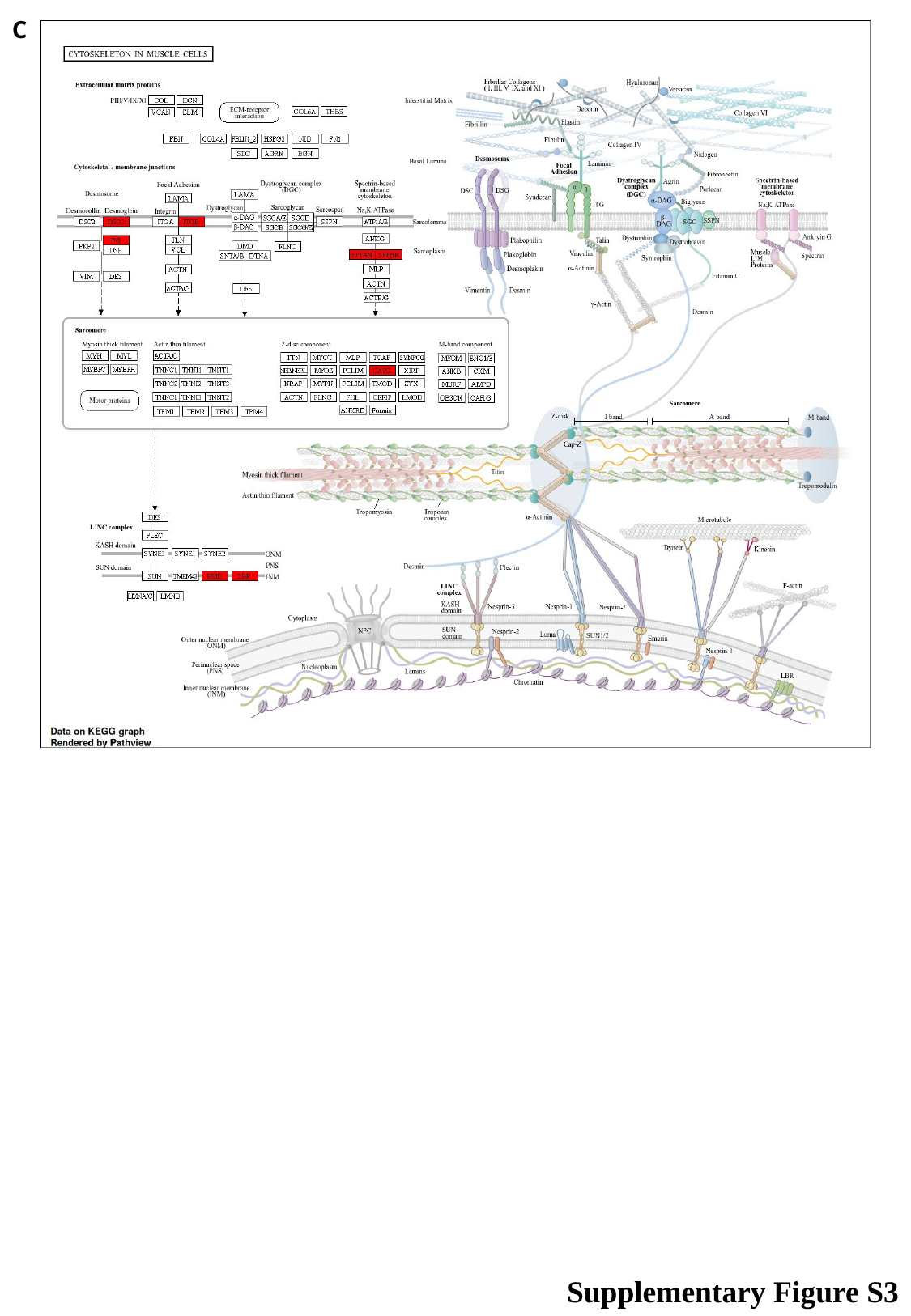

C
Supplementary Figure S3

## Slide 9
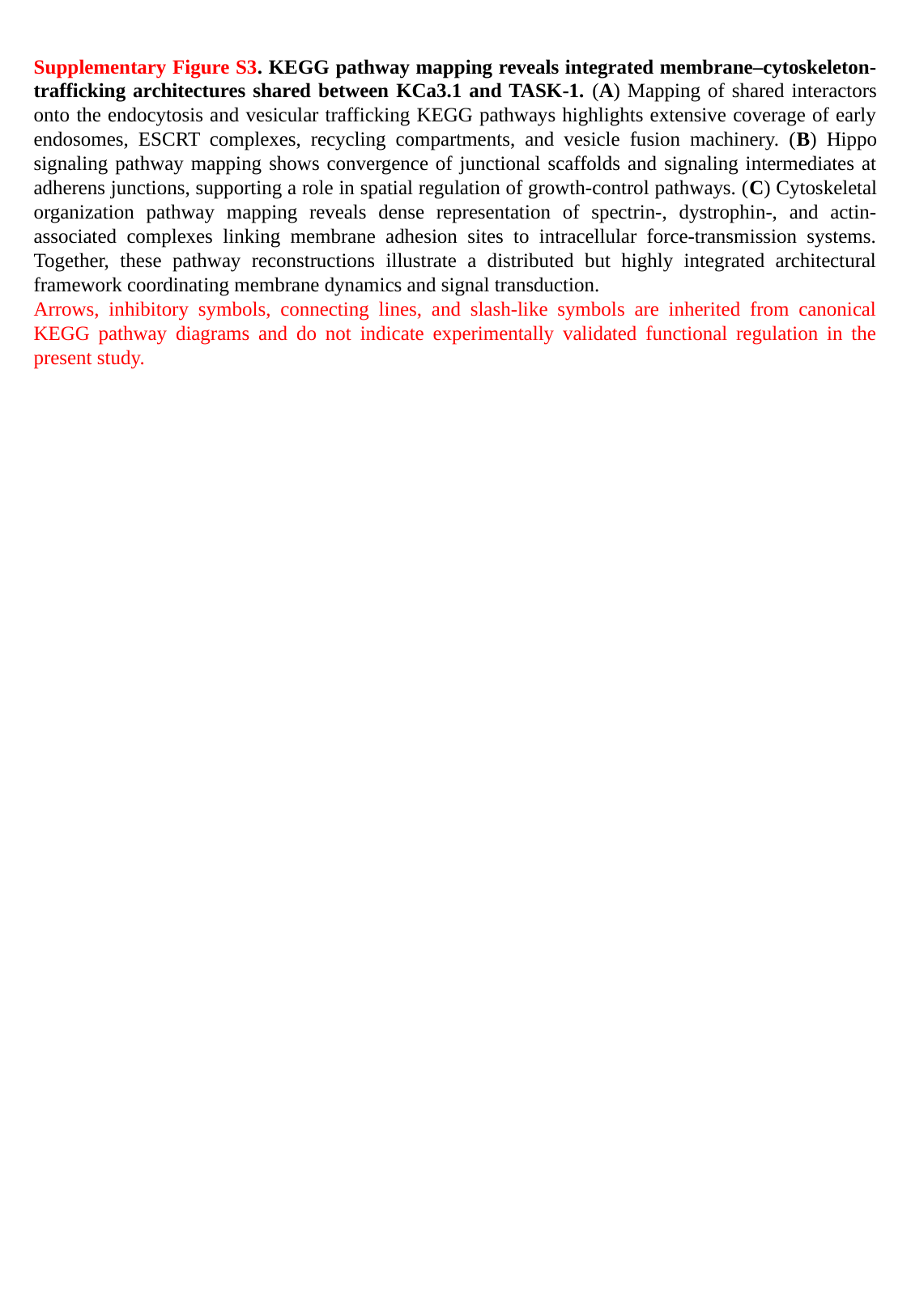

Supplementary Figure S3. KEGG pathway mapping reveals integrated membrane–cytoskeleton-trafficking architectures shared between KCa3.1 and TASK-1. (A) Mapping of shared interactors onto the endocytosis and vesicular trafficking KEGG pathways highlights extensive coverage of early endosomes, ESCRT complexes, recycling compartments, and vesicle fusion machinery. (B) Hippo signaling pathway mapping shows convergence of junctional scaffolds and signaling intermediates at adherens junctions, supporting a role in spatial regulation of growth-control pathways. (C) Cytoskeletal organization pathway mapping reveals dense representation of spectrin-, dystrophin-, and actin-associated complexes linking membrane adhesion sites to intracellular force-transmission systems. Together, these pathway reconstructions illustrate a distributed but highly integrated architectural framework coordinating membrane dynamics and signal transduction.
Arrows, inhibitory symbols, connecting lines, and slash-like symbols are inherited from canonical KEGG pathway diagrams and do not indicate experimentally validated functional regulation in the present study.

## Slide 10
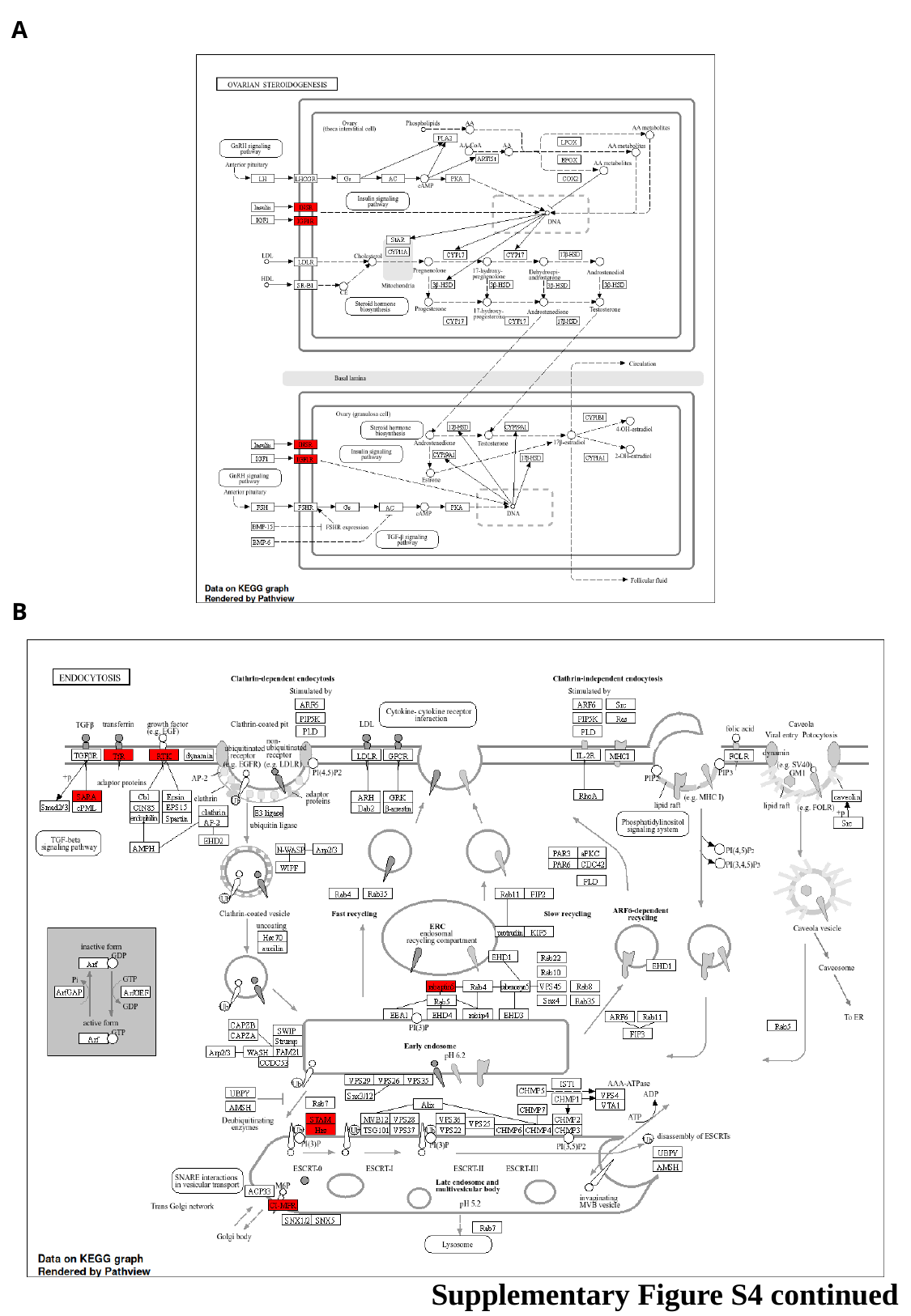

A
B
Supplementary Figure S4 continued

## Slide 11
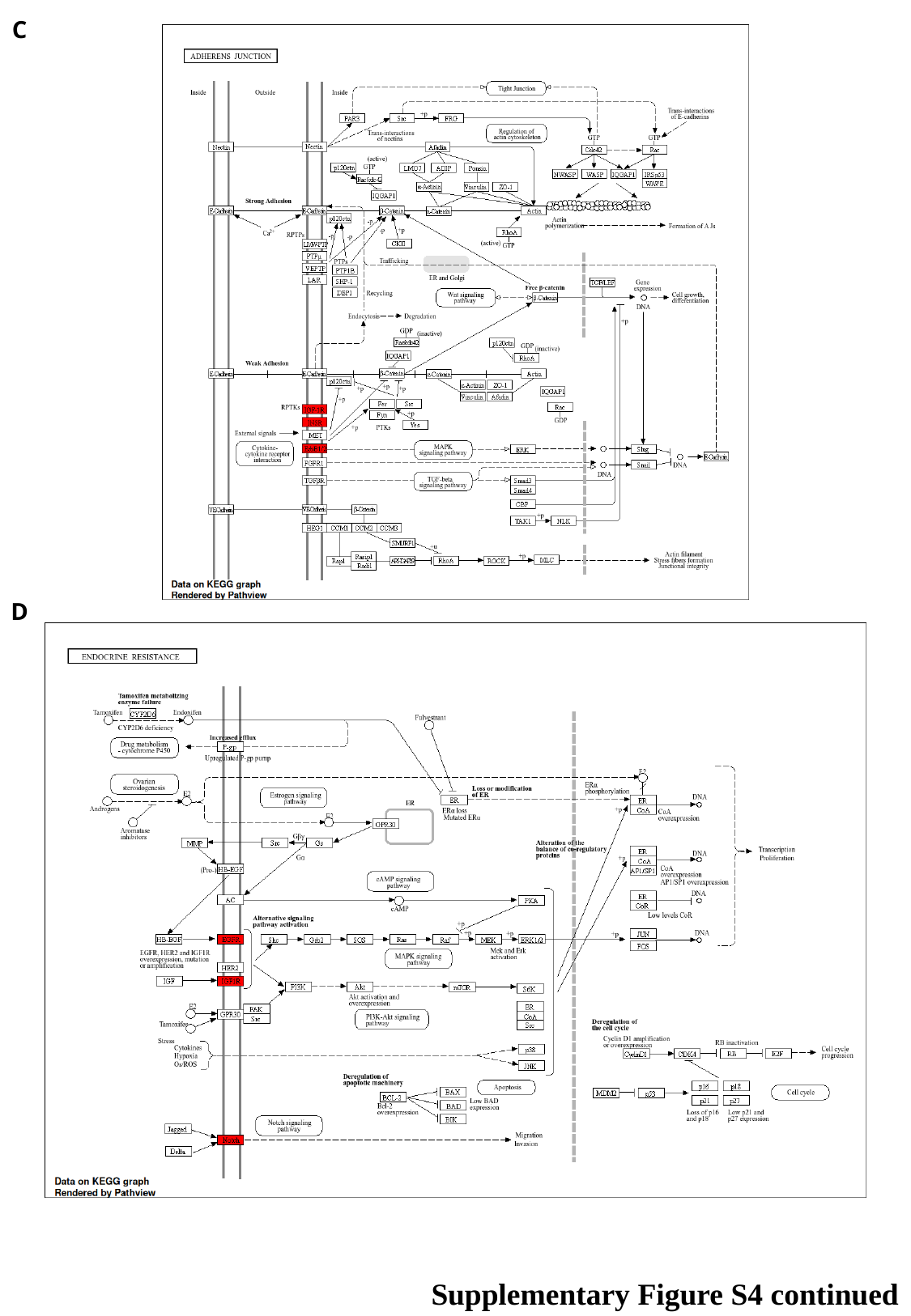

C
D
Supplementary Figure S4 continued

## Slide 12
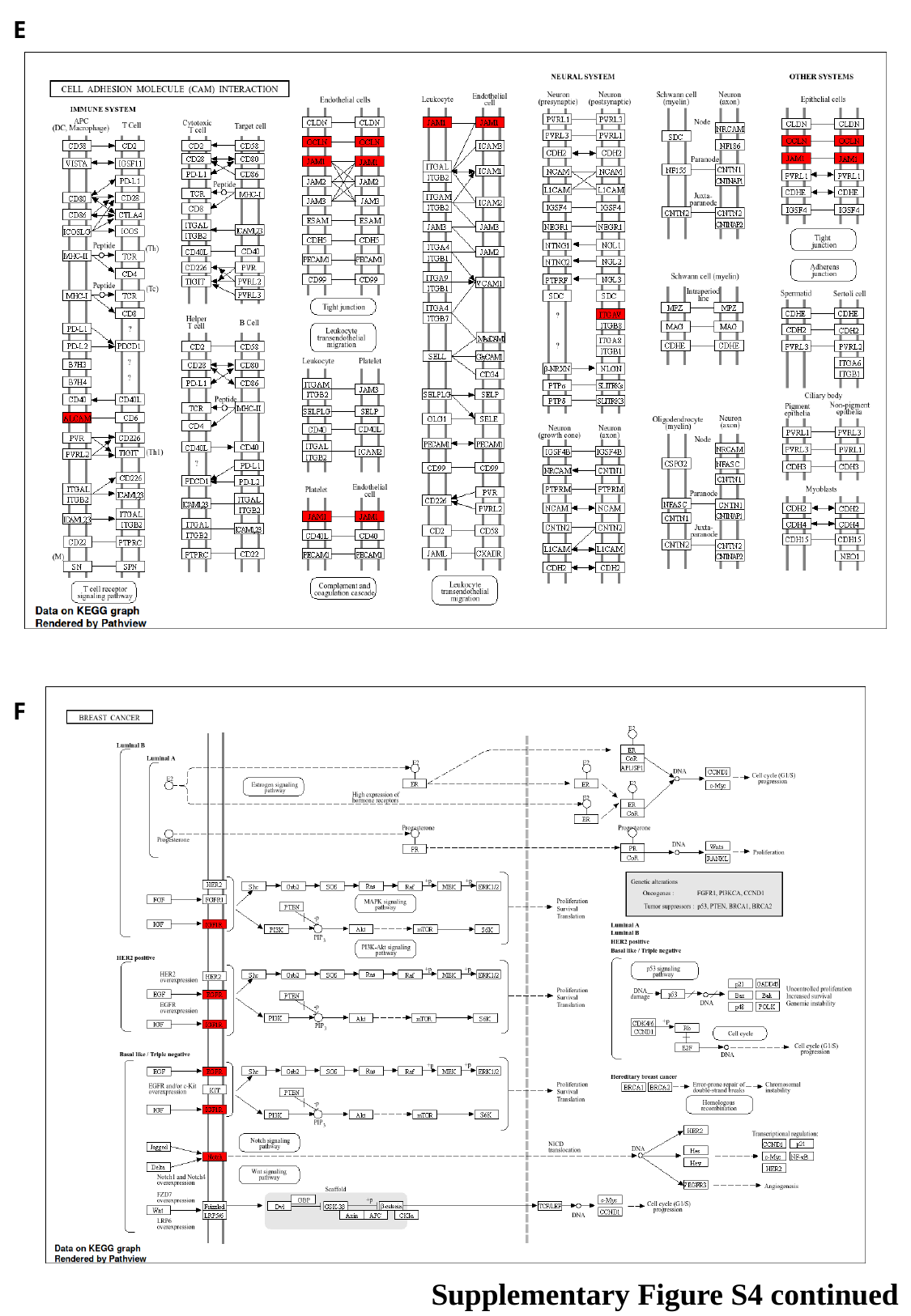

E
F
Supplementary Figure S4 continued

## Slide 13
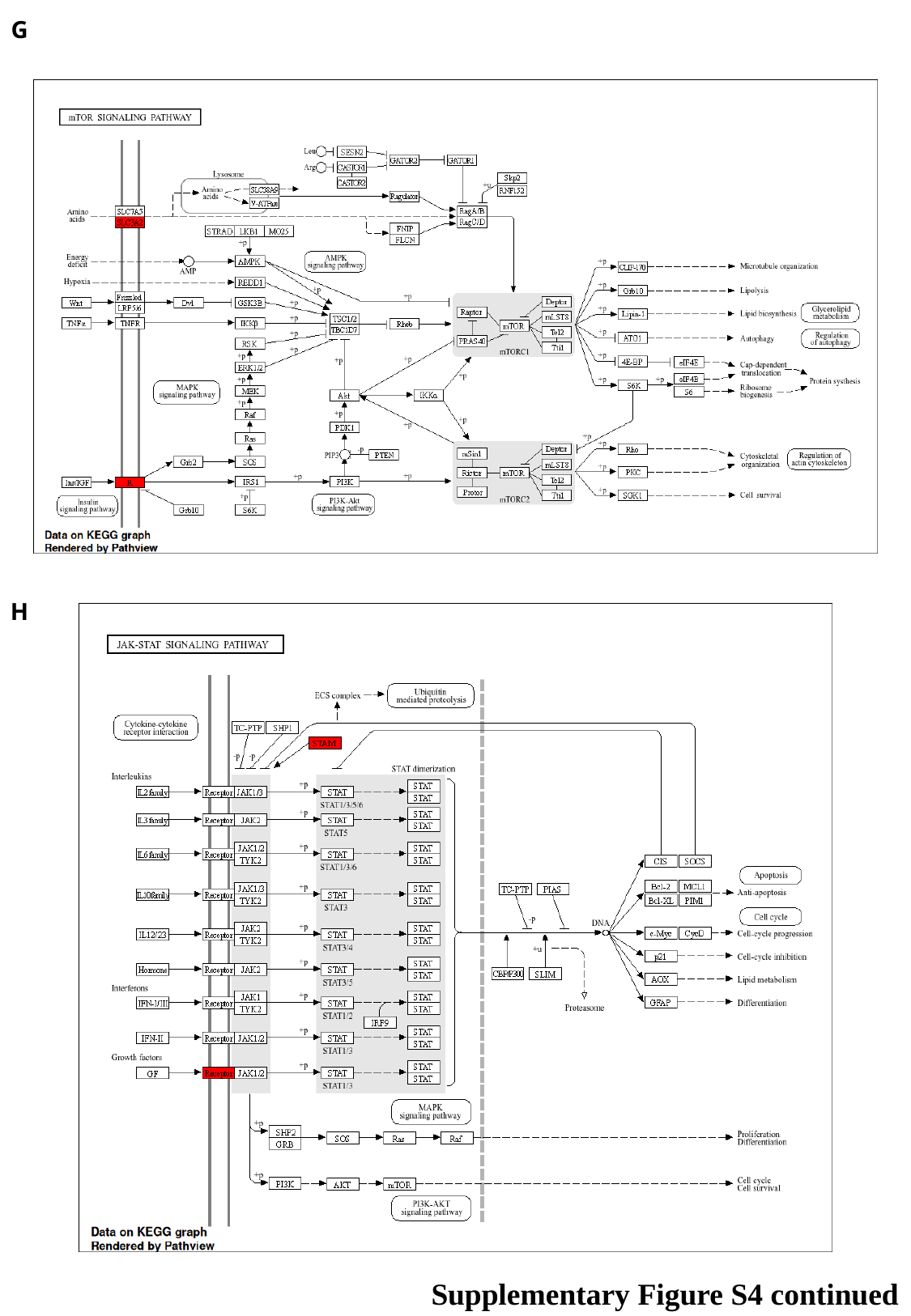

G
H
Supplementary Figure S4 continued

## Slide 14
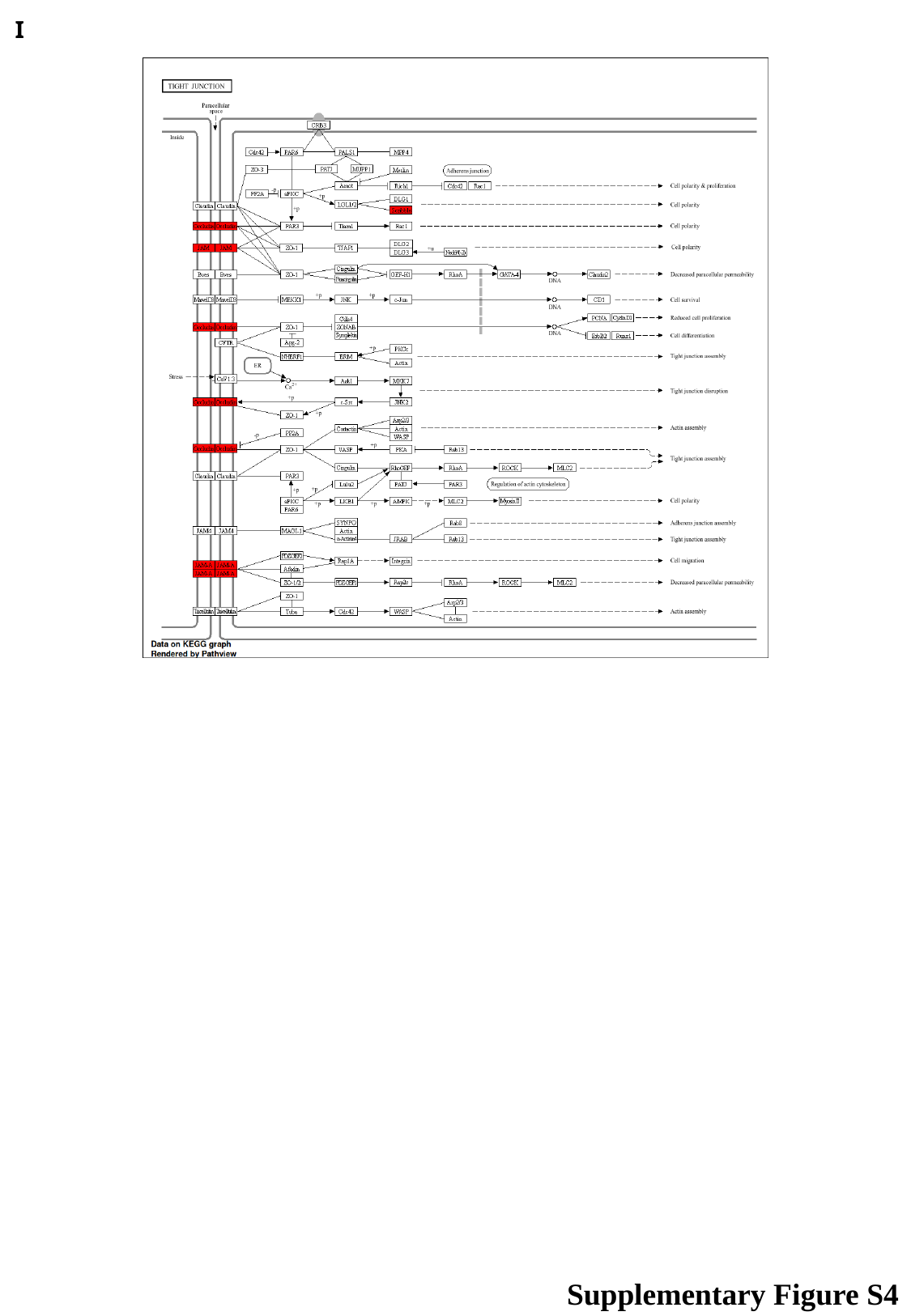

I
Supplementary Figure S4

## Slide 15
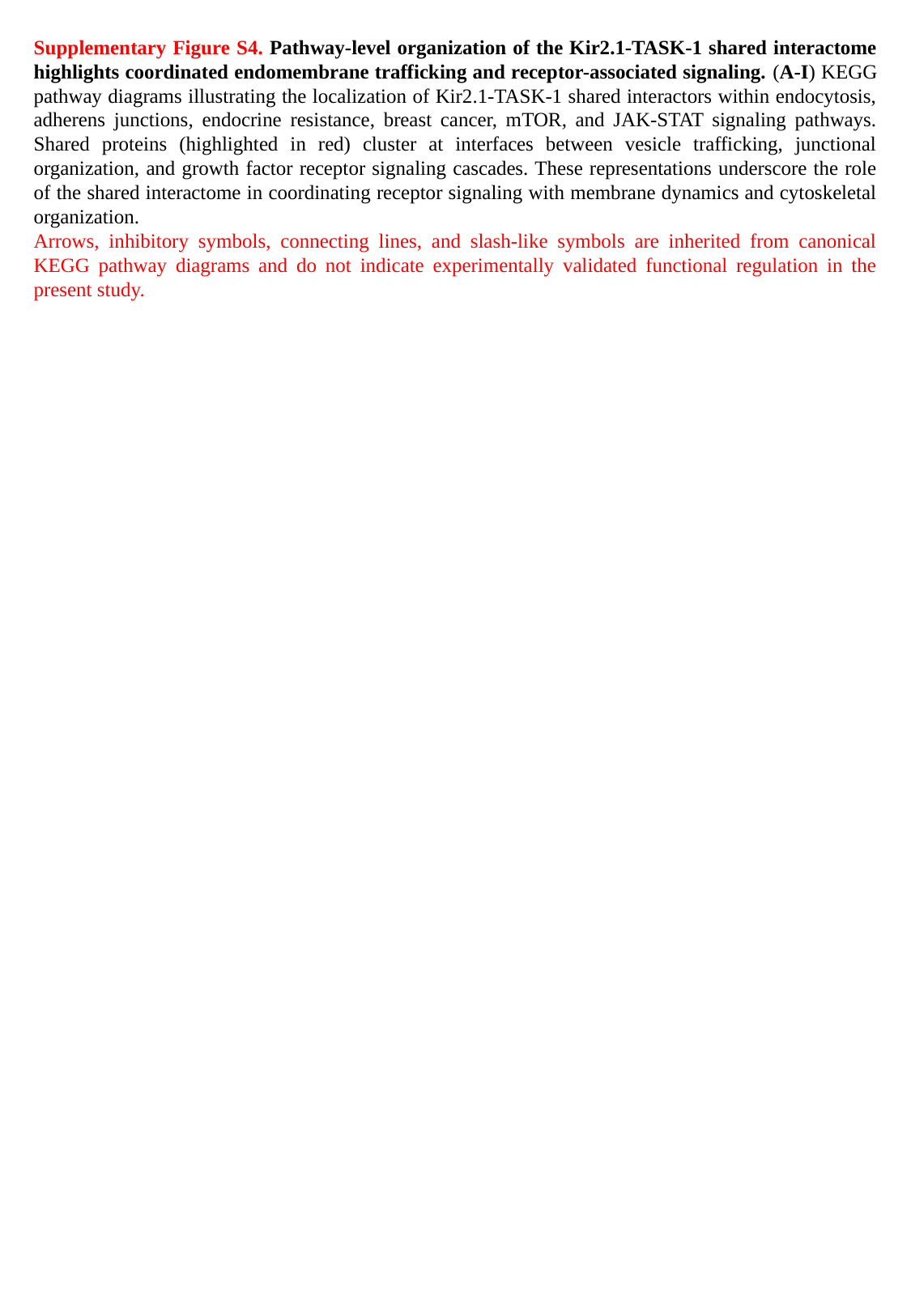

Supplementary Figure S4. Pathway-level organization of the Kir2.1-TASK-1 shared interactome highlights coordinated endomembrane trafficking and receptor-associated signaling. (A-I) KEGG pathway diagrams illustrating the localization of Kir2.1-TASK-1 shared interactors within endocytosis, adherens junctions, endocrine resistance, breast cancer, mTOR, and JAK-STAT signaling pathways. Shared proteins (highlighted in red) cluster at interfaces between vesicle trafficking, junctional organization, and growth factor receptor signaling cascades. These representations underscore the role of the shared interactome in coordinating receptor signaling with membrane dynamics and cytoskeletal organization.
Arrows, inhibitory symbols, connecting lines, and slash-like symbols are inherited from canonical KEGG pathway diagrams and do not indicate experimentally validated functional regulation in the present study.
