# Supplementary material for: Comparative Interactome Analysis Reveals Architectural Principles Governing K+ Channel Function in Cancer
Source: Int J Mol Sci. 2026 Jun 29;27(13):5862. doi: 10.3390/ijms27135862 (PMC13362327; doi:10.3390/ijms27135862)
Supplement: Supplementary file 1 [file ijms-27-05862-s001.zip › new-TableS1.pdf]

Abbreviation / Symbol	Full name	Functional / pathway context
**K^+**	Potassium ion	Ion conducted by potassium channels
**PPI**	Protein–protein interaction	Physical or proximity-based protein association
**GO**	Gene Ontology	Functional annotation system
**GO-BP / BP**	Gene Ontology Biological Process	Biological process enrichment category
**GO-CC / CC**	Gene Ontology Cellular Component	Subcellular localization enrichment category
**GO-MF / MF**	Gene Ontology Molecular Function	Molecular activity enrichment category
**KEGG**	Kyoto Encyclopedia of Genes and Genomes	Pathway database used for enrichment and pathway mapping
**FDR**	False discovery rate	Multiple-testing-adjusted significance value
**STRING**	Search Tool for the Retrieval of Interacting Genes/Proteins	Database used for PPI network reconstruction and enrichment
**BioID**	Proximity-dependent biotin identification	Proximity labelling method using BirA*
**TurboID**	Engineered biotin ligase-based proximity labelling	Rapid proximity labelling method
**APEX2**	Engineered ascorbate peroxidase 2	Enzyme used for proximity labelling
**AP-MS**	Affinity purification–mass spectrometry	Biochemical method for identifying protein associations
**Co-IP**	Co-immunoprecipitation	Biochemical method for recovering protein complexes
**LC-MS/MS**	Liquid chromatography–tandem mass spectrometry	Proteomic identification method
**TCGA**	The Cancer Genome Atlas	Cancer genomic and transcriptomic data source
**TPM**	Transcripts per million	RNA expression normalization unit
**KCa3.1 / KCNN4**	Potassium calcium-activated channel subfamily N member 4	Intermediate-conductance Ca^{2+}-activated K^+ channel
**Kir2.1 / KCNJ2**	Potassium inwardly rectifying channel subfamily J member 2	Inward rectifier K^+ channel
**ROMK / ROMK2 / Kir1.1 / KCNJ1**	Renal outer medullary potassium channel / potassium inwardly rectifying channel subfamily J member 1	Inward rectifier K^+ channel
**TASK-1 / KCNK3**	Potassium two-pore domain channel subfamily K member 3	Two-pore-domain leak K^+ channel
**Kv1.3 / KCNA3**	Potassium voltage-gated channel subfamily A member 3	Voltage-gated K^+ channel
**hERG / Kv11.1 / KCNH2**	Human ether-à-go-go-related gene / potassium voltage-gated channel subfamily H member 2	Voltage-gated K^+ channel
**STIM1**	Stromal interaction molecule 1	ER Ca^{2+} sensor; regulates store-operated Ca^{2+} entry
**STIM2**	Stromal interaction molecule 2	ER Ca^{2+} sensor related to STIM1

**ITGB4**	Integrin subunit beta 4	Adhesion receptor; links signalling to adhesion structures
**ITGA6**	Integrin subunit alpha 6	Adhesion receptor; basement membrane and focal adhesion signalling
**EGFR**	Epidermal growth factor receptor	Receptor tyrosine kinase; growth factor signalling
**ERBB2 / HER2**	Erb-b2 receptor tyrosine kinase 2	Receptor tyrosine kinase involved in cancer signalling
**IGF2R**	Insulin-like growth factor 2 receptor	Receptor-associated trafficking and signalling
**NOTCH2**	Notch receptor 2	Receptor involved in developmental and cancer signalling
**NECTIN2**	Nectin cell adhesion molecule 2	Cell–cell adhesion and junctional organization
**SCRIB**	Scribble planar cell polarity protein	Polarity and junctional scaffold
**DLG**	Discs large family proteins	Junctional scaffold proteins
**MPP7**	Membrane palmitoylated protein 7	Polarity and junctional scaffold
**NF2**	Neurofibromin 2 / Merlin	Cytoskeletal and Hippo pathway-associated scaffold
**JUP**	Junction plakoglobin	Desmosomal/adherens junction component
**DSG2**	Desmoglein 2	Desmosomal cadherin
**ALCAM**	Activated leukocyte cell adhesion molecule	Cell adhesion molecule
**CTNNB1**	Catenin beta 1 / β-catenin	Adherens junction and Wnt signalling component
**CTNND1**	Catenin delta 1 / p120-catenin	Cell–cell junction and adhesion regulator
**HGS**	Hepatocyte growth factor-regulated tyrosine kinase substrate	Endosomal sorting and ESCRT-associated trafficking
**USP8**	Ubiquitin-specific peptidase 8	Endosomal sorting and receptor trafficking
**PSD3**	Pleckstrin and Sec7 domain containing 3	Endosomal trafficking and signalling adaptor
**ZFYVE16**	Zinc finger FYVE-type containing 16	Endosomal membrane-associated protein
**TSG101**	Tumor susceptibility gene 101	ESCRT-I complex component
**STAM**	Signal transducing adaptor molecule	ESCRT-associated endosomal sorting
**GGA1 / GGA2 / GGA3**	Golgi-localized, gamma adaptin ear-containing ARF-binding proteins	Vesicle trafficking adaptors
**RAB proteins**	Ras-related proteins in brain	Small GTPases regulating vesicle trafficking
**SEC23 / SEC24 / SEC31**	COPII coat complex proteins	ER-to-Golgi vesicle transport
**SEC22B**	SEC22 homolog B	SNARE protein involved in vesicular trafficking

**USE1**	Unconventional SNARE in the ER 1	ER/Golgi trafficking SNARE
**SNAP47**	Synaptosome-associated protein 47	SNARE-associated vesicle trafficking protein
**TRIP11**	Thyroid hormone receptor interactor 11 / GMAP-210	Golgi-associated trafficking/tethering protein
**SCFD1**	Sec1 family domain containing 1	Vesicle trafficking regulator
**AP2M1**	Adaptor-related protein complex 2 subunit mu 1	Clathrin-mediated endocytosis
**ESCRT**	Endosomal sorting complexes required for transport	Endosomal sorting and multivesicular body pathway
**COPII**	Coat protein complex II	ER-to-Golgi transport machinery
**SNARE**	Soluble NSF attachment protein receptor	Vesicle fusion machinery
**CAV1**	Caveolin 1	Membrane microdomain/caveolae component
**CD44**	CD44 molecule	Adhesion, migration, and cancer-associated signalling
**PDGFRβ / PDGFRB**	Platelet-derived growth factor receptor beta	Receptor tyrosine kinase signalling
**PI3K–Akt**	Phosphoinositide 3-kinase–Akt pathway	Growth, survival, and oncogenic signalling pathway
**Hippo pathway**	Hippo signalling pathway	Controls growth, polarity, and contact-dependent signalling
**Wnt pathway**	Wingless/Int signalling pathway	Developmental and cancer-associated signalling
**Ras / RAS**	Rat sarcoma GTPase family	Small GTPase oncogenic signalling pathway
**mTOR**	Mechanistic target of rapamycin	Growth, metabolism, and nutrient signalling
**JAK–STAT**	Janus kinase–signal transducer and activator of transcription pathway	Cytokine and growth factor signalling
**STAT3**	Signal transducer and activator of transcription 3	Transcription factor involved in survival and cancer signalling
**TP53 / p53**	Tumor protein p53	Tumor suppressor transcription factor
**AGK**	Acylglycerol kinase	Lipid kinase; regulates phospholipid signalling
**DGKE**	Diacylglycerol kinase epsilon	Lipid kinase; regulates diacylglycerol/phosphoinositide signalling
**PIP4K2A**	Phosphatidylinositol-5-phosphate 4-kinase type 2 alpha	Phosphoinositide metabolism
**VDAC1 / VDAC2**	Voltage-dependent anion channel 1/2	Mitochondrial outer membrane transport
**TOMM20**	Translocase of outer mitochondrial membrane 20	Mitochondrial protein import
**ATP5A1**	ATP synthase F1 subunit alpha	Oxidative phosphorylation
**OPA1**	OPA1 mitochondrial dynamin-like GTPase	Mitochondrial dynamics and cristae organization

**PHB2**	Prohibitin 2	Mitochondrial organization and stress response
**AIFM1**	Apoptosis-inducing factor mitochondria associated 1	Mitochondrial redox/apoptosis-associated protein
**HSPA9**	Heat shock protein family A member 9 / Mortalin	Mitochondrial chaperone
**HSPA5 / BiP**	Heat shock protein family A member 5 / Binding immunoglobulin protein	ER chaperone and unfolded protein response
**CANX**	Calnexin	ER protein folding chaperone
**CALR**	Calreticulin	ER chaperone and Ca^{2+}-binding protein
**DERL proteins**	Derlin family proteins	ER-associated degradation components
**CCT complex**	Chaperonin-containing TCP1 complex	Cytosolic protein folding machinery
**ERAD**	Endoplasmic reticulum-associated degradation	Protein quality-control pathway
**UPR**	Unfolded protein response	ER stress response pathway
**HIF-1α / HIF1A**	Hypoxia-inducible factor 1 alpha	Hypoxia and metabolic adaptation pathway
**PRKDC**	Protein kinase, DNA-activated, catalytic subunit	DNA damage response and signalling
**PARP1**	Poly(ADP-ribose) polymerase 1	DNA repair and stress response
**SDHA**	Succinate dehydrogenase complex flavoprotein subunit A	Mitochondrial electron transport chain
**ACLY**	ATP citrate lyase	Metabolic enzyme linking citrate metabolism to lipid synthesis
**EPB41 / EPB41L2**	Erythrocyte membrane protein band 4.1 / band 4.1-like 2	Membrane–cytoskeleton organization
**IQGAP1**	IQ motif containing GTPase activating protein 1	Cytoskeletal scaffold and signalling regulator
**CAMLG**	Calcium-modulating cyclophilin ligand	Ca^{2+}-associated signalling
**CAPZB**	Capping actin protein of muscle Z-line subunit beta	Actin cytoskeleton regulation
