# Supplementary material for: Comparative Interactome Analysis Reveals Architectural Principles Governing K+ Channel Function in Cancer
Source: Int J Mol Sci. 2026 Jun 29;27(13):5862. doi: 10.3390/ijms27135862 (PMC13362327; doi:10.3390/ijms27135862)
Supplement: Supplementary file 1 [file ijms-27-05862-s001.zip › revised-table-legend.pdf]

Supplementary Table S1. Glossary of abbreviations, pathway labels, and selected protein symbols used throughout the manuscript and supplementary figures.
Definitions of abbreviations, pathway annotations, Gene Ontology categories, KEGG pathway terms, and selected protein symbols used in the main text and supplementary figures. This glossary is provided to facilitate interpretation of enrichment analyses, pathway maps, protein–protein interaction networks, and other graphical representations presented throughout the study.

Supplementary Table S2. STRING enrichment analysis of Biological Process (BP) terms for the shared interactome between KCa3.1 (KCNN4) and Kir2.1 (KCNJ2).
Functional enrichment analysis was performed using the STRING database (version 12.0). The input dataset corresponds to proteins shared between KCa3.1 and Kir2.1 interactomes. Enriched Gene Ontology Biological Process (GO-BP) terms are reported. Statistical significance was assessed using false discovery rate (FDR)-adjusted p-values. Only significantly enriched terms (FDR < 0.05) are shown. STRING-based GO Biological Process enrichment highlights over-representation of terms related to cell–cell junction organization, regulation of cell adhesion, and membrane-associated signalling. These results indicate that the shared interactome is functionally oriented toward the spatial organization of membrane-proximal signalling platforms rather than ion transport.

Supplementary Table S3. STRING enrichment analysis of Cellular Component (CC) terms for the shared interactome between KCa3.1 (KCNN4) and Kir2.1 (KCNJ2).
Enrichment analysis was performed using STRING (v12.0) on the set of proteins shared between KCa3.1 and Kir2.1 interactomes. Enriched Gene Ontology Cellular Component (GO-CC) terms are listed. Statistical significance was determined using FDR-corrected p-values. Only terms with FDR < 0.05 are reported. Enriched GO Cellular Component terms map shared interactors to adherens junctions, lateral plasma membrane, and focal adhesions. This localization bias supports the existence of a membrane-proximal scaffold integrating adhesion and signalling processes.

Supplementary Table S4. STRING enrichment analysis of Molecular Function (MF) terms for the shared interactome between KCa3.1 (KCNN4) and Kir2.1 (KCNJ2).
Gene Ontology Molecular Function (GO-MF) enrichment analysis was conducted using STRING (version 12.0). The input protein set corresponds to the overlap between KCa3.1 and Kir2.1 interactomes. Significance was assessed using FDR-adjusted p-values, and only enriched terms with FDR < 0.05 are shown. Enriched GO Molecular Function categories are dominated by protein binding, adaptor activity, and receptor-associated interactions. This profile indicates that shared interactors primarily function as organizational nodes rather than catalytic effectors.

Supplementary Table S5. STRING enrichment analysis of Biological Process (BP) terms for the shared interactome between KCa3.1 (KCNN4) and TASK-1 (KCNK3).
Gene Ontology Biological Process enrichment analysis was performed using STRING (v12.0). The dataset includes proteins shared between KCa3.1 and TASK-1 interactomes. Statistical significance was determined using FDR-adjusted p-values. Only significantly enriched terms (FDR < 0.05) are reported. GO Biological Process terms are enriched for vesicle-mediated transport, endocytosis, and intracellular protein trafficking, indicating that functional overlap between these channels is embedded within membrane dynamics rather than ion conduction.

Supplementary Table S6. STRING enrichment analysis of Molecular Function (MF) terms for the shared interactome between KCa3.1 (KCNN4) and TASK-1 (KCNK3).
Enrichment analysis of Gene Ontology Molecular Function terms was performed using STRING (version 12.0). The input dataset corresponds to proteins shared between KCa3.1 and TASK-1 interactomes. Only significantly enriched terms (FDR < 0.05) are shown. Enriched Molecular Function terms include cytoskeletal binding, receptor interaction, and adaptor-related activities, consistent with a role in coordinating membrane-associated signalling complexes.

Supplementary Table S7. STRING enrichment analysis of Cellular Component (CC) terms for the shared interactome between KCa3.1 (KCNN4) and TASK-1 (KCNK3).
Cellular Component enrichment was performed using STRING (v12.0) on the shared protein set between KCa3.1 and TASK-1 interactomes. Statistical significance was assessed using FDR-adjusted p-values. Only enriched terms with FDR < 0.05 are reported. Shared interactors are enriched in vesicular compartments, endomembrane systems, and cytoskeleton-associated structures, supporting a multi-compartment organizational framework.

Supplementary Table S8. KEGG pathway enrichment analysis of the shared interactome between Kir2.1 (KCNJ2) and TASK-1 (KCNK3).
KEGG pathway enrichment analysis was performed using the STRING database (version 12.0). The input dataset corresponds to proteins shared between Kir2.1 and TASK-1 interactomes. Pathways are ranked based on statistical significance (FDR-adjusted p-values). Gene count indicates the number of proteins associated with each pathway. Enriched pathways include endocytosis, vesicle-mediated transport, and receptor-associated signalling modules, indicating integration of membrane trafficking with growth factor signalling.

Supplementary Table S9. KEGG pathway enrichment analysis of the shared interactome between Kir2.1 (KCNJ2) and TASK-1 (KCNK3).
KEGG pathway enrichment analysis was conducted on the shared protein set between Kir2.1 and TASK-1 interactomes. Enriched pathways are reported along with statistical significance values (FDR-adjusted p-values) and associated gene counts. Only significantly enriched pathways (FDR < 0.05) are shown. Additional enriched pathways reinforce the involvement of receptor signalling networks and membrane dynamics, highlighting the architectural coupling of signalling and trafficking processes.

Supplementary Table S10. STRING enrichment analysis of Biological Process (BP) terms for the shared interactome between Kir2.1 (KCNJ2) and TASK-1 (KCNK3).
Gene Ontology Biological Process enrichment analysis was performed using STRING (version 12.0). The dataset includes proteins shared between Kir2.1 and TASK-1 interactomes. Statistical significance was evaluated using FDR-adjusted p-values, and only enriched terms with FDR < 0.05 are reported. Dominant enriched processes include COPII-mediated transport, receptor-mediated endocytosis, and intracellular protein trafficking, supporting a central role for endomembrane organization.

Supplementary Table S11. STRING enrichment analysis of Biological Process (BP) terms for the shared interactome between Kir2.1 (KCNJ2) and TASK-1 (KCNK3).

Gene Ontology Biological Process enrichment analysis was performed using STRING (v12.0). The dataset includes proteins shared between Kir2.1 and TASK-1 interactomes. Enriched terms are reported with FDR-adjusted p-values. Only significantly enriched terms (FDR < 0.05) are shown. Additional enriched processes expand the representation of trafficking, protein localization, and vesicle organization pathways, reinforcing the central role of membrane dynamics.

Supplementary Table S12. STRING enrichment analysis of Cellular Component (CC) terms for the shared interactome between Kir2.1 (KCNJ2) and TASK-1 (KCNK3).
Gene Ontology Cellular Component enrichment analysis was performed using the STRING database (version 12.0). The input dataset consists of proteins shared between Kir2.1 and TASK-1 interactomes. Statistical significance was assessed using false discovery rate (FDR)-adjusted p-values. Only enriched terms with FDR < 0.05 are reported. Shared interactors localize to ESCRT complexes, vesicular membranes, and anchoring junctions, indicating integration of trafficking and adhesion-related structures.

Supplementary Table S13. Extended STRING enrichment analysis of Cellular Component (CC) terms for the shared interactome between Kir2.1 (KCNJ2) and TASK-1 (KCNK3).
An extended Cellular Component enrichment analysis was performed using STRING (v12.0), including lower-ranking but statistically significant compartments. This table provides a broader view of subcellular localization patterns beyond the top enriched categories. Only terms with FDR < 0.05 are reported. Beyond core junctional and vesicular compartments, additional enriched terms indicate a wider distribution across membrane-bound organelles, supporting a distributed architectural organization.

Supplementary Table S14. STRING enrichment analysis of Molecular Function (MF) terms for the shared interactome between Kir2.1 (KCNJ2) and TASK-1 (KCNK3).
Gene Ontology Molecular Function enrichment analysis was performed using STRING (v12.0). The dataset corresponds to proteins shared between Kir2.1 and TASK-1 interactomes. Enriched terms are reported with FDR-adjusted p-values, and only statistically significant terms (FDR < 0.05) are shown. Enriched categories include growth factor receptor binding, cadherin binding, and ubiquitin-related interactions, indicating a scaffold-driven signaling architecture.

Supplementary Table S15. Extended STRING enrichment analysis of Molecular Function (MF) terms for the shared interactome between Kir2.1 (KCNJ2) and TASK-1 (KCNK3).
An extended Molecular Function enrichment analysis was performed using STRING (v12.0), including additional lower-ranking but significant functional categories. This table complements Table 14 by providing a more comprehensive overview of functional annotations associated with the shared interactome. Additional categories reinforce the role of shared interactors as signalling adaptors and structural coordinators rather than enzymatic regulators.

Supplementary Table S16. STRING enrichment analysis of Molecular Function (MF) terms for the shared interactome between KCa3.1 (KCNN4), Kir2.1 (KCNJ2), and TASK-1 (KCNK3).

Gene Ontology Molecular Function enrichment analysis was performed using STRING (v12.0) on the protein set shared across all three channel interactomes. Enriched terms are reported with FDR-adjusted p-values. Only statistically significant categories (FDR < 0.05) are shown. The shared core is enriched in binding and adaptor activities, indicating that higher-order overlap selectively retains organizational nodes within signalling networks.

Supplementary Table S17. STRING enrichment analysis of Biological Process (BP) terms for the shared interactome between KCa3.1 (KCNN4), Kir2.1 (KCNJ2), and TASK-1 (KCNK3).
Biological Process enrichment analysis was performed using STRING (v12.0) on the triple-intersection protein set. Enriched processes are reported with FDR-adjusted p-values. Only significantly enriched terms (FDR < 0.05) are included. Enriched processes include vesicle-mediated transport and junction organization, indicating that convergence across channels occurs at the level of shared architectural principles.

Supplementary Table S18. High-confidence Kv1.3 interactome identified by co-immunoprecipitation and LC-MS/MS.
Proteins were identified by co-immunoprecipitation of Kv1.3 followed by LC-MS/MS analysis. Only proteins detected with unique peptides and meeting high-confidence identification criteria are included. This dataset represents experimentally validated Kv1.3-associated proteins. Proteins identified by co-immunoprecipitation and LC-MS/MS include mitochondrial, metabolic, and stress-response components, supporting a mitochondria-centered architectural organization of Kv1.3-associated signalling.

Supplementary Table S19. Functional classification and cluster assignment of the 89 proteins shared between independent Kv1.3 co-immunoprecipitation datasets.
The table reports the 89 proteins identified in both the co-immunoprecipitation dataset generated by Prosdocimi et al. and the independent Kv1.3 co-immunoprecipitation dataset originally generated by Capera et al. Proteins were assigned to functional categories on the basis of Gene Ontology annotations, KEGG pathway associations, STRING network organization, and manual biological curation. Functional groups include mitochondria and bioenergetics, protein quality control, ER-associated processes, vesicle trafficking, RNA metabolism, cytoskeletal organization, DNA repair, lipid metabolism, and signalling-related modules. These classifications were used to evaluate the degree of biological convergence between independent biochemical interactome datasets and to identify organizational themes preserved across experimental contexts.

Supplementary Table S20. Functional classification and cluster assignment of the 104 proteins shared between the Kv1.3 BioID and co-immunoprecipitation interactomes.
The table reports the 104 proteins identified in both the Kv1.3 BioID interactome described by Prosdocimi et al. and the corresponding filtered co-immunoprecipitation dataset. Proteins were grouped into functional categories using Gene Ontology annotations, literature-supported functional information, STRING network organization, and manual biological curation. Assigned categories include protein quality control, RNA processing and gene expression, endocytosis and membrane dynamics, vesicle trafficking, cytoskeletal organization, junctional and polarity-associated proteins, signalling networks, cell cycle and DNA repair pathways, nuclear transport,

and additional regulatory components. The resulting classification provides a framework for identifying technique-independent features of the Kv1.3-associated interaction landscape.

Supplementary Table S21. Comparative analysis of proteins shared between Jurkat and HEK293 Kv1.3 interactomes.
The table reports the proteins identified in both the TurboID-derived Jurkat T-cell interactome and the BioID-derived HEK293 Kv1.3 interactome. Shared proteins were classified using Gene Ontology annotations, KEGG pathway associations, STRING network analysis, and manual biological curation into functional categories including protein quality control, RNA metabolism, endocytosis and membrane dynamics, vesicle trafficking, cytoskeletal organization, junctional and polarity-associated proteins, signalling pathways, cell cycle and DNA repair modules, nuclear transport, metabolic regulation, organelle crosstalk, and additional shared regulators. This classification was used to evaluate the extent to which Kv1.3-associated organizational features are preserved across distinct cellular contexts and proximity-labelling methodologies.
